# A chromosome-level, haplotype-resolved genome assembly for the barn owl, *Tyto alba*

**DOI:** 10.1093/gigascience/giag092

**Published:** 2026-09-08

**Authors:** Hugo Corval, Anne-Lyse Ducrest, Marianne Bachmann Salvy, Allison Burns, Alexandros Topaloudis, Céline Simon, Elisa Cora, Daniel Cavaleri, Bettina Almasi, Alexandre Roulin, Christian Iseli, Nicolas Guex, Tristan Cumer, Jérôme Goudet

**Affiliations:** Department of Ecology and Evolution, University of Lausanne, Biophore, Quartier UNIL-Sorge, CH-1015 Lausanne, Switzerland; Swiss Institute of Bioinformatics, Amphipôle, Quartier UNIL-Sorge, CH-1015 Lausanne, Switzerland; Department of Ecology and Evolution, University of Lausanne, Biophore, Quartier UNIL-Sorge, CH-1015 Lausanne, Switzerland; Department of Ecology and Evolution, University of Lausanne, Biophore, Quartier UNIL-Sorge, CH-1015 Lausanne, Switzerland; Swiss Institute of Bioinformatics, Amphipôle, Quartier UNIL-Sorge, CH-1015 Lausanne, Switzerland; Bioinformatics Competence Center (BICC), École Polytechnique Fédérale de Lausanne, Bâtiment AAB, Station 19, CH-1015 Lausanne, Switzerland; Bioinformatics Competence Center (BICC), University of Lausanne, Génopode, Quartier UNIL-Sorge, CH-1015 Lausanne, Switzerland; Department of Ecology and Evolution, University of Lausanne, Biophore, Quartier UNIL-Sorge, CH-1015 Lausanne, Switzerland; Swiss Institute of Bioinformatics, Amphipôle, Quartier UNIL-Sorge, CH-1015 Lausanne, Switzerland; Department of Ecology and Evolution, University of Lausanne, Biophore, Quartier UNIL-Sorge, CH-1015 Lausanne, Switzerland; Gene Expression Core Facility (GECF), School of Life Sciences, École Polytechnique Fédérale de Lausanne, Bâtiment SV, Station 19, CH-1015 Lausanne, Switzerland; Gene Expression Core Facility (GECF), School of Life Sciences, École Polytechnique Fédérale de Lausanne, Bâtiment SV, Station 19, CH-1015 Lausanne, Switzerland; Swiss Ornithological Institute, Seerose 1, CH-6204 Sempach, Switzerland; Department of Ecology and Evolution, University of Lausanne, Biophore, Quartier UNIL-Sorge, CH-1015 Lausanne, Switzerland; Bioinformatics Competence Center (BICC), École Polytechnique Fédérale de Lausanne, Bâtiment AAB, Station 19, CH-1015 Lausanne, Switzerland; Bioinformatics Competence Center (BICC), University of Lausanne, Génopode, Quartier UNIL-Sorge, CH-1015 Lausanne, Switzerland; Bioinformatics Competence Center (BICC), École Polytechnique Fédérale de Lausanne, Bâtiment AAB, Station 19, CH-1015 Lausanne, Switzerland; Bioinformatics Competence Center (BICC), University of Lausanne, Génopode, Quartier UNIL-Sorge, CH-1015 Lausanne, Switzerland; Department of Ecology and Evolution, University of Lausanne, Biophore, Quartier UNIL-Sorge, CH-1015 Lausanne, Switzerland; Swiss Institute of Bioinformatics, Amphipôle, Quartier UNIL-Sorge, CH-1015 Lausanne, Switzerland; Department of Ecology and Evolution, University of Lausanne, Biophore, Quartier UNIL-Sorge, CH-1015 Lausanne, Switzerland; Swiss Institute of Bioinformatics, Amphipôle, Quartier UNIL-Sorge, CH-1015 Lausanne, Switzerland

**Keywords:** *Tyto alba*, Strigiformes, chromosome-level, haplotype-resolved, genome assembly, microchromosomes, chromosomal inversion

## Abstract

**Background:**

Recent advances in long-read sequencing have enabled near telomere-to-telomere (T2T) assemblies across diverse taxa. However, avian genomes remain challenging due to numerous microchromosomes, small, typically <20Mb, DNA molecules that are gene-, GC-, and repeat-rich. As a consequence, microchromosomes are often missing from genome assemblies.

**Results:**

Here, we present a chromosome-level, haplotype-resolved genome assembly for the Western barn owl (*Tyto alba*). Using a trio-binning strategy with Illumina parental reads combined with PacBio HiFi and Oxford Nanopore Technologies data, we generated 2 phased contig sets. These were scaffolded into 40 linkage groups using a linkage map. Comparative analyses identified unplaced HiFi scaffolds corresponding to microchromosomes, which we integrated into 6 additional microchromosomes using long reads information. The 2 assemblies present 46 chromosomes, matching the karyotype of the species. They exhibit strong synteny between parental haplotypes, except for a ∼38 Mb complex region on chromosome 7 containing nested inversions.

**Conclusions:**

This high-quality reference provides a haplotype-resolved and chromosome-level genome for *Strigiformes*, enabling fine-scale studies of structural variation and avian genome evolution

## Background

In recent years, genomics has entered the telomere-to-telomere (T2T) era. This shift began with the first complete human genome (T2T-CHM13) [1] and quickly expanded to other species, from crops [2, 3] to livestock [4] and primates [5]. These milestones were achieved thanks to the advent of long-read sequencing technologies, mostly led by Pacific Biosciences (PacBio) and Oxford Nanopore Technologies (ONT), combined with chromosome conformation contact Hi-C sequencing [6]. With this resolution, assemblies now expose complex regions, such as the major histocompatibility complex or the killer-cell immunoglobulin receptors locus, that were previously poorly characterized due to extreme polymorphism and repetitive structure [7–11].

Although these recent advances have transformed genome assembly, significant challenges remain. One difficulty of genome assembly is reconstructing both parental haplotypes in diploid species rather than a mosaic consensus. Common strategies include trio binning with parental short reads [12, 13], Hi-C phasing coupled to long, accurate reads [14, 15], and Strand-seq for chromosome length phasing [16, 17]. When successful, haplotype-resolved assemblies can improve the discovery of complex structural variants (SVs) [12, 18–20]. Still, phasing can be limited by repeat- and GC-rich regions that promote switches or mis-joins [21], by genomes with large size, high heterozygosity or polyploidy (common in plants) [22], and by practical constraints such as the need for parental samples (trio binning) and the cost/complexity of specialized libraries. Consequently, fully phased references are still less common than haploid assembly in many taxa [11, 22].

The genome reconstruction also varies widely across taxa. In vertebrates, bird genomes are difficult to assemble due to the presence of numerous microchromosomes [23, 24]. These microchromosomes share features that hamper their reconstruction: they are small, gene-rich, GC-rich and contain a high proportion of repetitive elements [24, 25]. Because of these properties, many avian genome assemblies contain fewer chromosomes than the actual karyotype, with microchromosomes frequently missing or not fully resolved and left as unplaced scaffolds [24]. Recently, the introduction of Oxford Nanopore ultra-long reads has improved their recovery, leading to more complete assemblies in species such as chicken [26], bustard [27], and mallard [25, 28]. However, the adoption of these approaches across avian genomes has been constrained by practical challenges, including difficulties in obtaining high-quality samples, extracting enough ultra-high molecular DNA, and the cost of sequencing. As a result, achieving full and accurate resolution of avian microchromosomes remains a major challenge.

Among owls (*Strigiformes)*, genome assembly poses extra difficulties. Their karyotypes feature many microchromosomes and rearrangements [29], making synteny with well-assembled model species less informative [30]. The common barn owl (*Tyto alba*—NCBI:txid56313) illustrates this difficulty. The species has 46 pairs of chromosomes [31] and a complete genome assembly has yet to be achieved. The current assembly generated using PacBio long reads and Bionano optical mapping consists of 70 “pseudo-haplotypes,” i.e., scaffolds representing a mosaic combination of the 2 haplotypes in which phase information is not conserved (N50 = 36.03 Mb) [32]. This was a major improvement from the previous version, which contained 21,509 contigs with an N50 of 4.62 Mb [33]. Nevertheless, additional work is needed to reach a chromosome-level assembly that matches the 46 pairs observed in the barn owl karyotype.

In this study, we aim to produce a chromosome-level, haplotype-resolved reference genome for the barn owl (*T. alba*). We achieve accurate chromosome phasing through trio-binning using Illumina parental reads. We construct unitigs from HiFi PacBio and ONT data, which we then scaffold into chromosomes using linkage map information [34]. Finally, we leverage the gene-rich nature of microchromosomes to manually curate unplaced unitigs, based on the presence of genes typically found on microchromosomes in other species. With this integrated approach, we deliver a high-quality genomic resource, add a chromosome-level reference of an owl, and resolve previously uncharacterized complex structures.

## Results

### Chromosome-scale, haplotype resolved assemblies

**Figure 1:**
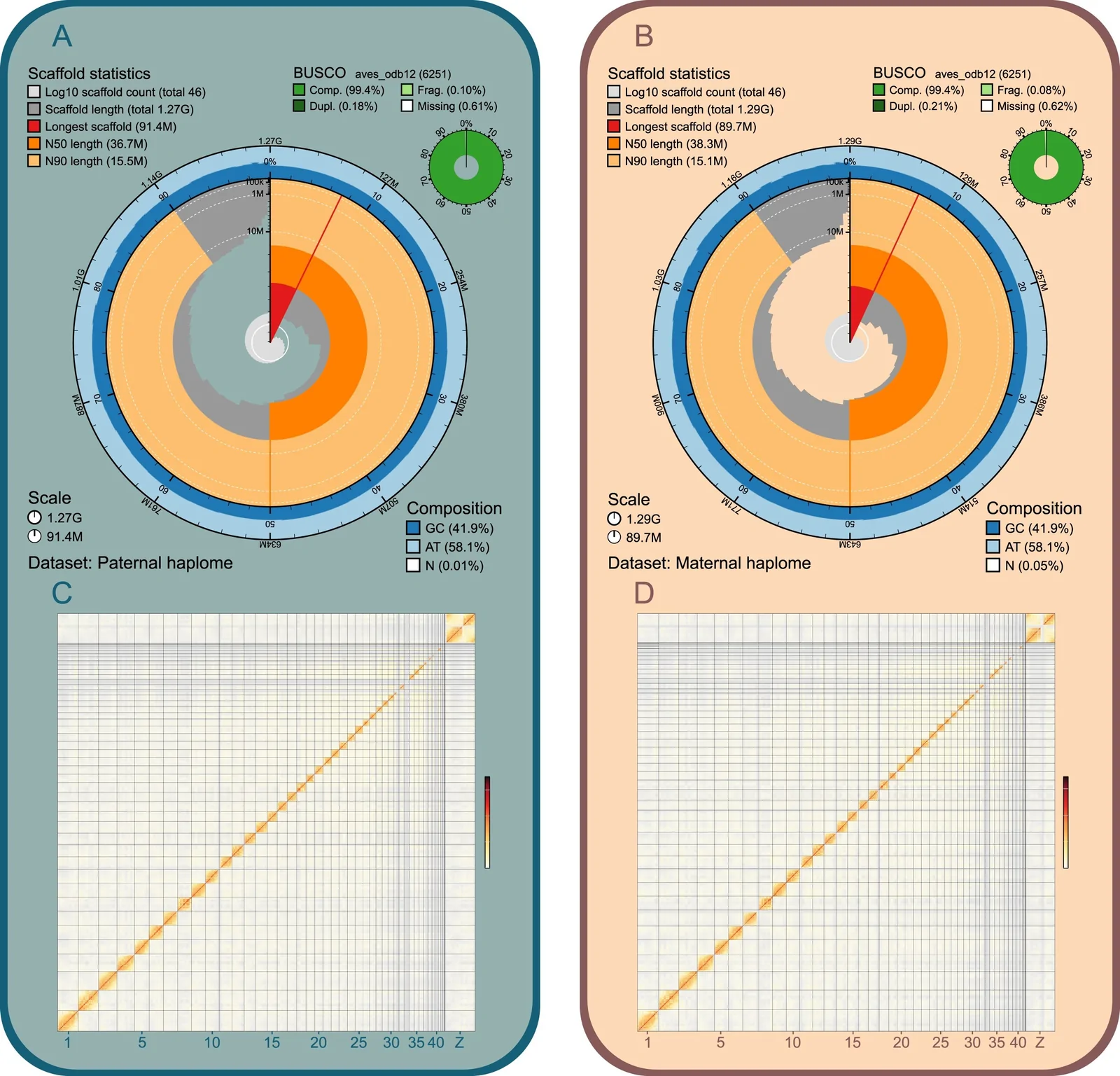
Contiguity, completeness, and chromatin interaction landscape of the paternal and maternal haplotype assemblies. (A–B) Snail plots display the overall structure and composition of the (A) paternal and (B) maternal assemblies. Each circumferential axis is divided into 1,000 bins, each representing 0.1% of the corresponding assembly. Scaffold length distribution is shown in dark grey, with the plot radius scaled to the longest scaffold (highlighted in red). N50 and N90 scaffold length thresholds are indicated in orange and pale orange, respectively. Nucleotide composition is displayed as dark blue for GC, light blue for AT, and white for ambiguous bases (N). A BUSCO completeness summary (Complete, Fragmented, Duplicated, Missing) is provided in the upper-right corner of each panel. (C–D) HiC contact maps for the (C) paternal and (D) maternal assemblies illustrate the genome-wide pattern of spatial interactions. Chromosomes are arranged by size from left to right and from top to bottom, with the Z chromosome positioned last. The main diagonal represents high-frequency intra-chromosomal contacts, while off-diagonal signals correspond to inter-chromosomal interactions. Interaction frequencies are displayed on a logarithmic scale. Heatmaps were generated using HiContacts.

Using PacBio HiFi reads (150.8 Gb, ∼coverage of 116x for a genome of 1.3 Gb, Supplementary Table 1), Oxford Nanopore reads (40.2 Gb, ∼coverage of 31x, Supplementary Table 1), and linkage information, we generated 2 chromosome-level, haplotype-resolved assemblies of the barn owl (*T. alba*). Each haplotype comprised 46 chromosomes, matching the expected karyotype (Fig. 1; Table 1; Supplementary Table 2). The paternal assembly spanned 1.27 Gbp (N50 = 36.7 Mb; N90 = 15.5 Mb, Fig. 1A), and the maternal assembly spanned 1.29 Gbp (N50 = 38.3 Mb; N90 = 15.1 Mb, Fig. 1B). Both haplomes showed a slight increase in size compared to the previous assembly (GCF_018691265.1: 1.25 Gbp, Table 1). Hi-C contact maps showed strong, chromosome-scale diagonals with no spurious trans-chromosomal signals, consistent with high contiguity and correct scaffolding (Fig. 1C, D).

**Table 1:** Comparison of general assembly statistics across barn owl (*Tyto alba*) references

	genome_assembly_l500	T.alba_DEE_v4.0	Paternal		Maternal	
	GCF_902150015.1	GCF_018691265.1	Prior to scaffolding [contigs]	After scaffolding [chromosomes]	Prior to scaffolding [contigs]	After scaffolding [chromosomes]
Assembly size	1,219,191,878	1,249,867,532	1,317,996,626	1,267,676,513	1,313,309,923	1,285,637,069
# sequences	21509	70	287	46	331	46
N50	4,615,526	36,032,128	26,240,345	36,723,072	24,830,383	38,271,680
N90	556,444	15,349,174	5,753,860	15,527,340	4,456,682	15,107,001
% GC	41.5	41.5	41.9	41.9	41.9	41.9
Total Ns	9,406,517	51,418,967	0	110,000	0	600,000
% BUSCO	98.4	98.5		99.4		99.4

Genomes compared include genome_assembly_l500 from Ducrest et al. [33] and T.alba_DEE_v4.0 from Machado et al. [32]. These reference genomes are shown alongside the 2 parental haplomes assembled here. Haplomes are each shown twice: a first column for the unscaffolded contigs, raw output from hifiasm, and a second column for the chromosome-scale assembly after filtering, scaffolding, and curation. Metrics include total assembly size, counts of sequences, N50/N90 (minimum length of sequences that cover a cumulative 50 or 90% of the entire assembled genome), percentage of GC, total Ns, and BUSCO completeness.

We quantified phasing accuracy by leveraging *k*–mers unique to each parental read set. For each haplotype assembly, we computed the switch error rate as the proportion of parental–specific *k*–mers incorrectly assigned to the opposite haplotype relative to the total number of parental–specific *k*–mers detected across both assemblies. This analysis indicated that 7.8% of paternal–specific *k*–mers were misplaced into the maternal haplotype, while 7.5% of maternal–specific *k*–mers were incorrectly assigned to the paternal haplotype.

**Figure 2:**
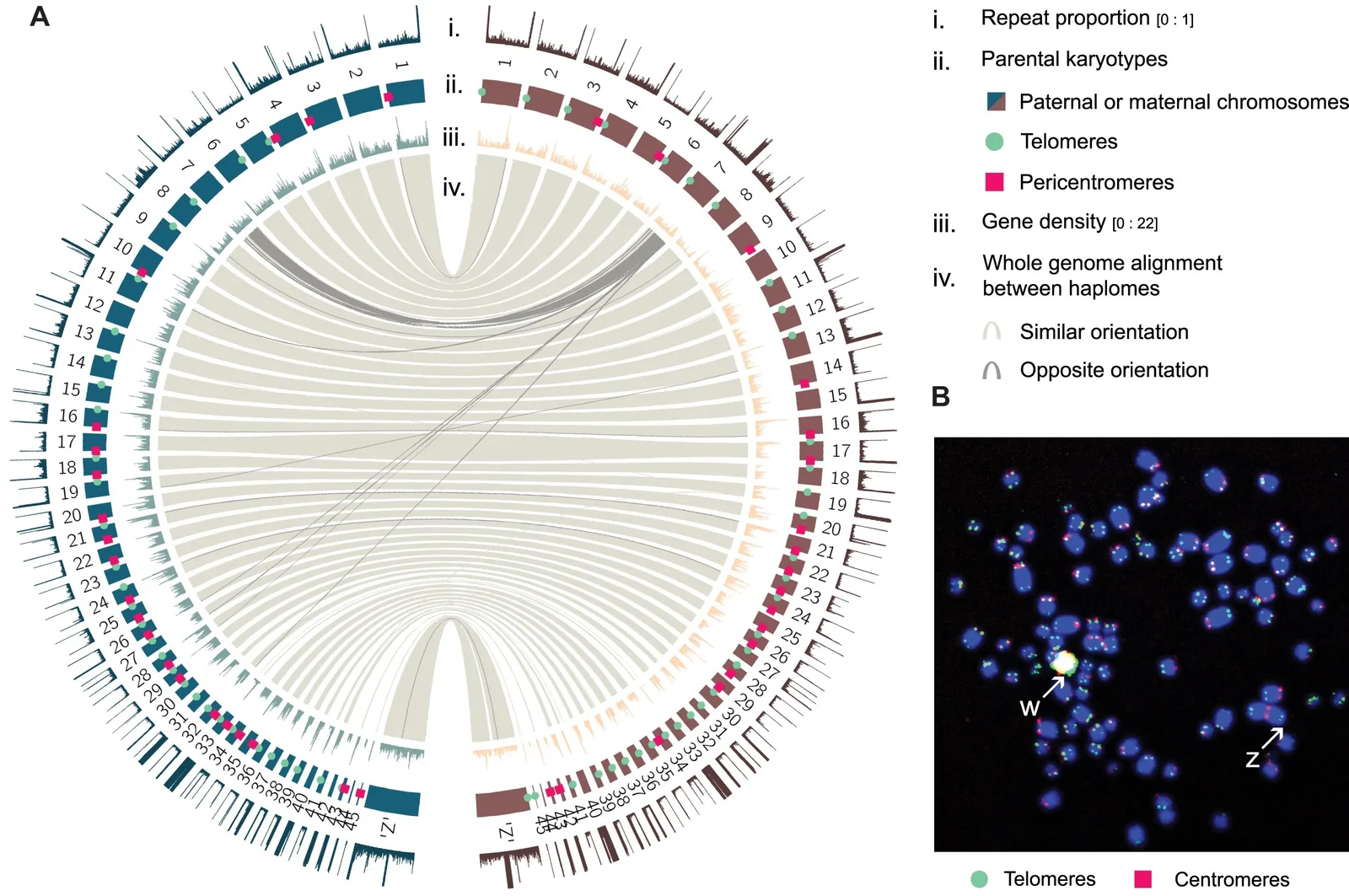
Overview of the paternal and maternal haplomes and their genome features. (A) The Circos plot provides a comparative summary of the genomic characteristics of the paternal haplome (left) and maternal haplome (right). From the outer to the inner track, the plots show the repeat proportion calculated in 100-kbp windows; the 46 chromosomes of each haplome, with regions matching telomeric and pericentromeric sequences indicated by circles and squares, respectively; the distribution of annotated genes, summarized in 100-kbp windows; and the whole-genome alignment between the 2 haplotypes. Because repetitive elements strongly affect alignment, both haplomes were hard-masked with RepeatMasker prior to alignment. Regions aligning in the same orientation are shown in light grey, and regions aligning in opposite orientation are shown in dark grey. (B) Fluorescence *in situ* hybridization (FISH) visualization of the *T. alba* karyotype of a female individual. Chromosomes are counterstained with DAPI. Centromeric regions are highlighted in magenta and telomeric regions in cyan using specific DNA probes (see the Material and methods section for details). Sexual chromosomes Z and W are indicated by arrows. FISH image in higher resolution can be found in Supplementary Fig. 7.

As the assembly was constructed from a homogametic male (ZZ), identification of the sexual chromosome could not be based on identification of a heterochromatide unique in each assembly. Instead, to identify the Z chromosome, we used a protein-based homology with other avian species. Species such as chicken (*Gallus gallus*) and zebra finch (*Taeniopygia guttata*) showed that proteins encoded on their Z chromosome mapped predominantly to our largest assembled chromosome (Supplementary Figs 1 and 2). This largest chromosome was also the only metacentric chromosome in the assembly, while all remaining chromosomes were acrocentric (Fig. 2B).

Relative to prior references, gaps were greatly reduced: 110,000 Ns (0.01%) in the paternal and 600,000 Ns (0.05%) in the maternal haplotype, compared with 51,418,967 Ns (4.11%) in T.alba_DEE_v4.0 (GCF_018691265.1, Table 1). GC content was similar across haplotypes (41.9% each) and only slightly higher than in earlier assemblies (0.1% increase compared to T.alba_DEE_v4.0). GC profiles were broadly homogeneous between chromosomes on average with a modest increase towards smaller chromosomes (Fig. 1A and B, Table 1, Supplementary Table 2).

BUSCO assessments indicated high completeness for both haplotypes (paternal: C = 99.4%, F = 0.10%, D = 0.18%, M = 0.61%; maternal: C = 99.4%, F = 0.08%, D = 0.21%, M = 0.62%), representing ∼1% improvement over T.alba_DEE_v4.0 (C = 98.5%).

To assess the amount of potential contamination, we ran blobplot analyses [35] at the contig level (i.e., prior to scaffolding) for both the maternal and paternal haplomes (Supplementary Figs 3 and 4). Both exhibited no evidence of contamination: most BLAST hits were assigned to *T. alba*, while a minor portion (tiniest contigs) corresponded to other avian species in both haplomes, as expected due to phylogenetic proximity [36].

To evaluate chromosome continuity, we quantified terminal features. In the paternal haplotype, 31 chromosomes contained telomeric sequence in 5p and 21 contained pericentromeric sequence. In the maternal haplotype, 34 and 18 chromosomes contained telomeric and pericentromeric sequence, respectively. Across haplotypes, 24 chromosomes were resolved from telomere to pericentromere, 18 contained only telomeric sequence, 2 centromeric sequence only, and 2 contained neither across haplomes (Fig. 2A, Supplementary Table 3).

### Repeat and gene annotation

Custom repeat annotation revealed that repeats constituted 13.68% (paternal) and 14.83% (maternal) of assembled sequence. LINEs were the predominant class (47.81% paternal and 47.07% maternal of total repeats), followed by LTR elements (32.23% paternal and 32.45% maternal, Supplementary Fig. 5). Repeats were enriched towards chromosomal ends and in the 6 smaller chromosomes. On the metacentric Z chromosome, repeat density peaked around the centromere (Fig. 2A).

To investigate the proportion and types of repeats on the W chromosome, which is absent from the focal male assembly, we performed fluorescence *in situ* hybridization (FISH) on a female individual (Fig. 2B). This technique uses fluorescent probes to localize specific DNA sequences on chromosomes, here targeting telomeric and centromeric repeats (see the Material and method section for details). The W chromosome exhibits strong signals for both probe types, suggesting a higher proportion of repeats in this chromosome compared to the rest of the barn owl genome.

**Figure 3:**
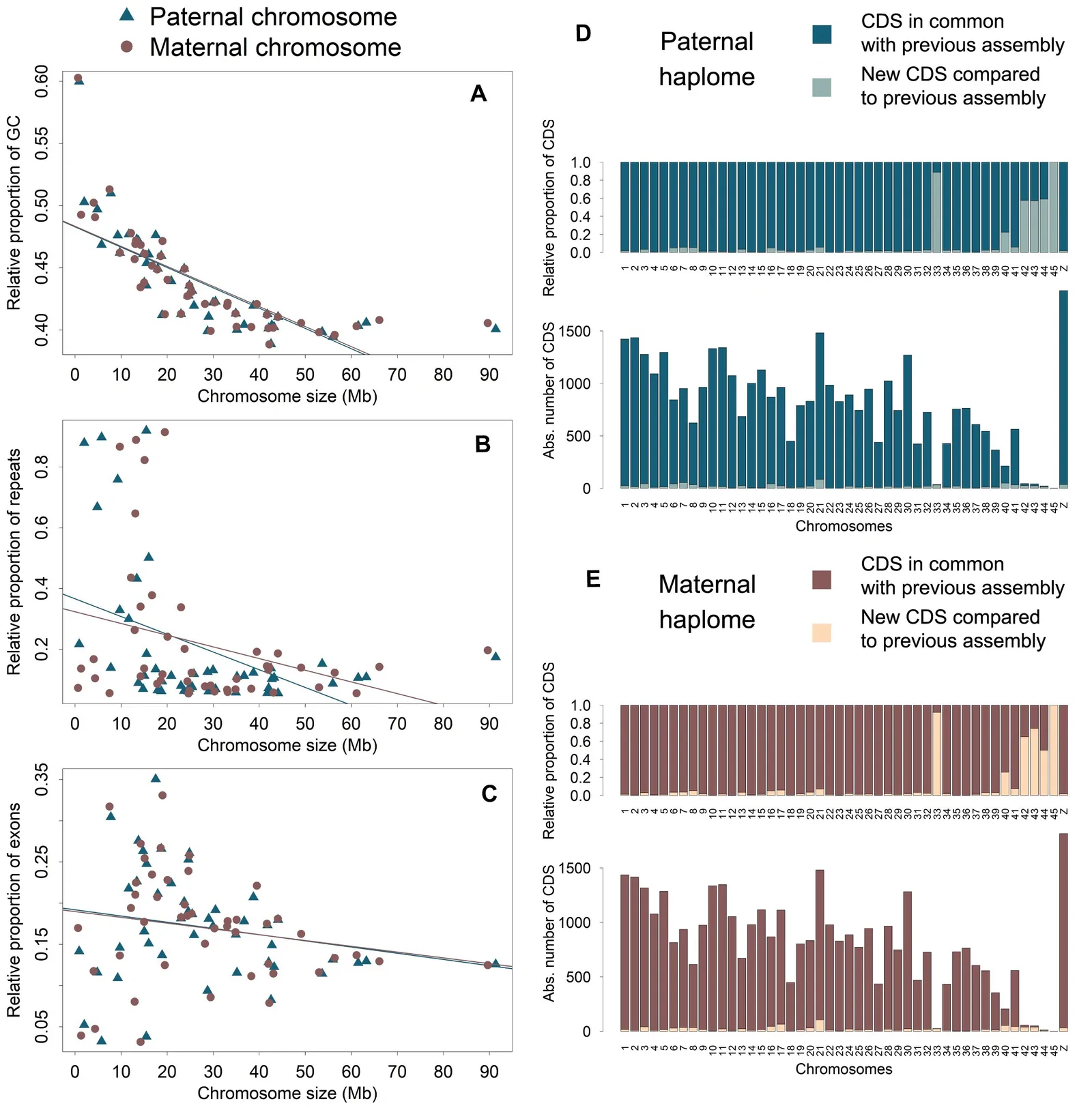
Dot chromosomes and their genomic characteristics. (A–C) Chromosome-wide distributions of GC content (A), repetitive elements (B), and exonic sequence (C) are shown for the paternal (triangle) and the maternal haplome (square). (D–E) Bar plots illustrate the absolute (lower panels) and relative proportion (upper panels) of newly annotated genes on each chromosome of the paternal (D) and maternal (E) haplomes. Newly annotated genes compared with the previous assembly are depicted in lighter colours.

Using the NCBI EGAPx workflow with multi-tissue RNA-seq support [33], we annotated 19,720 genes in the paternal haplotype and 20,039 in the maternal haplotype. These haplotype annotations were compared to the previous reference genome (T.alba_DEE_v4.0-GCF_018691265.1) by mapping back Coding DNA Sequences (CDS) to quantify newly annotated CDS. Newly annotated CDS totalled 908 in the paternal haplotype and 948 in the maternal haplotype and were distributed over the genome, with an enrichment towards the smallest chromosomes (Fig. 3D and E).

### Recovery and comparison of microchromosomes

To recover microchromosomes that were missing from the long-reads-based unitigs, we used MicroFinder [24]. This strategy allowed us to re-assign unplaced long-reads-based unitigs to specific microchromosomes and integrate them into the final haplotype assemblies.

We screened 116 unplaced unitigs in the paternal assembly and 119 in the maternal assembly. Based on conserved gene content, we identified 14 paternal and 18 maternal candidates using MicroFinder. We further assembled them in 6 new microchromosomes that were absent from previous versions (RefSeq: GCF_902150015.1 and GCF_018691265.1).

In contrast to chromosomes already present in the previous assembly, the 6 newly recovered chromosomes are characterized by distinct genomic properties and separate clearly from all remaining chromosomes in the PCA built from chromosomal features (Supplementary Fig. 6). The recovered microchromosomes showed elevated GC content, and most were extremely repeat-rich relative to larger chromosomes (Fig. 3A and B). They contained few genes in absolute terms and remained gene-sparse even after accounting for their size (Fig. 3C and E). Despite their sparse gene content, most of their annotated genes were absent from the prior assembly (GCF_018691265.1, Fig. 3D and E).

### Haplotype synteny and a complex rearrangement on chromosome 7

**Figure 4:**
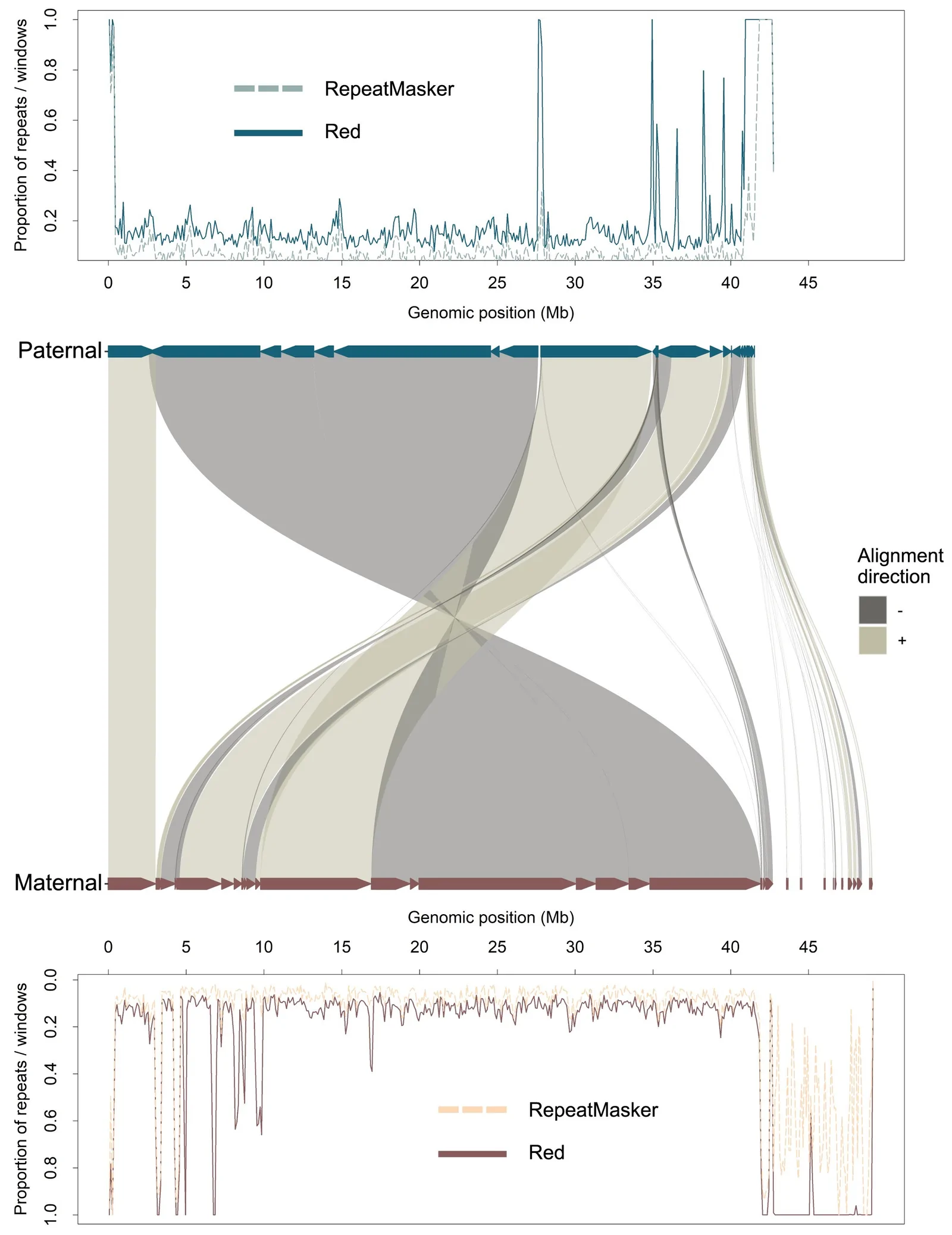
Large complex inversion on chromosome 7. Alignment between the paternal (top) and maternal (bottom) chromosome 7 highlights a major chromosomal inversion. The figure shows an extract from the whole-genome alignment, restricted to alignment blocks (arrows) that are exclusive to chromosome 7 in both haplomes. Apparent gaps, e.g., at the right end of the maternal chromosome 7, correspond to highly repetitive regions (as reflected in the repeat profile below) that align to other parts of the genome and are therefore not shown here. Regions aligning in the same orientation are displayed in light grey, whereas regions aligned in the opposite orientation are shown in dark grey. Repeat proportion, calculated in 100-kbp windows, is plotted along the top and bottom. Two repeat detection methods are shown: RepeatMasker, represented in a lighter colour with a dashed line, and Red, represented in a dark colour with a solid line.

Whole-genome alignment between haplotypes indicated extensive collinearity punctuated by a single, large and complex rearrangement on chromosome 7 (Fig. 2A). Nearly the entire paternal chromosome 7 aligned in the opposite orientation to the maternal chromosome 7, with nested structure: a large (∼20 Mb) inversion interleaved with 2 shorter inverted segments and separated by 2 collinear blocks, all translocated relative to each other (Fig. 4A). Repeat density increased near inferred breakpoints in both haplotypes, supporting the potential evolution of successive rearrangements (Fig. 4A) [37].

## Discussion

Recent advances in long-read sequencing technologies have made it increasingly possible to reconstruct near-complete, telomere-to-telomere chromosomes across an increasing range of species [1–5, 8, 11]. Yet birds remain challenging vertebrates to assemble owing to their numerous, small, GC-rich and repeat–dense microchromosomes, which have historically been under–represented or mis–assembled in reference genomes [7, 23, 24]. Here, we present high-quality haplotype–resolved, chromosome–level assemblies for the Western barn owl (*T. alba*), each comprising all 46 chromosomes expected from the karyotype [31, 38] and substantially improving upon previous barn owl reference genomes [32, 33]. These 2 haplotype-resolved assemblies exhibit high contiguity and completeness (with very few gaps and BUSCO scores above 99%) and recover 6 small chromosomes that were absent from earlier assemblies, providing a near–complete representation of the species’ chromosomal assembly.

### A multi-layered assembly strategy

No single sequencing technology was sufficient to resolve the barn owl genome. Instead, the assembly succeeded because multiple complementary resources were integrated, with each compensating for the others’ blind spots. First, we phased the haplomes using Illumina reads from the parents, partitioning offspring long reads with trio aware *k*-mers before assembly to reduce switches and produce cleaner haplotype-specific contigs for downstream scaffolding. Then, HiFi contigs formed the structural backbone of the assembly, providing the accuracy and read length necessary to produce long, high–confidence unitigs. However, Hi-Fi reads alone cannot reliably span extremely GC–rich or repeat–dense regions, features abundant on avian micro- and dot chromosomes [39]. ONT ultra–long reads filled this gap, traversing repetitive sequences and bridging discontinuities between HiFi contigs, consistent with earlier reports demonstrating their unique value for difficult regions in bird genomes [25, 26].

For chromosomes represented in the linkage map [34], population–based marker order provided a scaffold for organizing HiFi/ONT contigs into linkage groups, after which Hi–C contact data confirmed chromosome–scale structure for the larger elements. However, Hi–C proved uninformative for microchromosomes: their short length and high repeat and GC content result in reduced mappability (Supplementary Fig. 8), which in turn limits the reliable detection of meaningful contact patterns, a difficulty also encountered in other avian assemblies [24]. To identify and assemble these highly challenging chromosomes, we instead relied on conserved microchromosome gene–set signature [24]. This allowed to pinpoint unplaced unitigs enriched for microchromosome–specific genes. These fragments were then manually curated and stitched together, guided by ONT long–read alignments that provided the only long–range continuity through repetitive landscapes.

Altogether, the assembly demonstrates that the integration of all the available information was critical to construct a robust, near–complete chromosomal assembly. Our recommendation to facilitate good future assemblies would be to collect enough material to generate several HiFi libraries of different fragment sizes and collect enough ONT sequences, preferably as long as possible, before starting the assembly. Filtering of sequences containing high density of internal repeats, such as pericentromeric or centromeric reads, is also essential to prevent assembly artefacts. Although these regions are genuine components of the genome, as shown by FISH signals (Fig. 2B), they are difficult to assemble reliably without dedicated approaches and can therefore introduce biases into the reconstruction of the rest of the genome.

### Recovery and characteristics of the missing chromosomes

Bird genomes remain difficult to assemble fully, and only a handful of avian references currently include the entire set of microchromosomes [23, 25, 26, 28, 40, 41]. However, synteny–based scaffolding is often insufficient for microchromosomes because they show rapid evolution and poor conservation of gene order across species [23, 24].

We assembled 6 previously missing chromosomes for the barn owl, displaying hallmark features of micro/dot chromosomes (elevated GC content and enriched repeats) consistent with other avian genomes [25, 26, 28]. However, in contrast with the micro-chromosomes present in the previous assembly, they remain gene–sparse relative to expectations based on the classic view of microchromosomes as strongly gene–dense [23, 24]. This pattern may reflect genuine biological variation in the barn owl lineage but also residual annotation challenges in small repeat–rich elements now referred to as dot chromosomes [42].

Based on our feature–based analysis, the distinction between macro–, micro–, and dot–chromosomes is not straightforward, and no single variable (e.g., length, GC content, or gene content) provides clear boundaries between these chromosomal categories. Future work should examine the functional significance of genes located on these newly identified chromosomes and explore whether particular gene families (such as immunity– or sensory–related genes) exhibit micro–/dot–chromosome–specific organization in owls.

### Phased assemblies reveal complex structural variation

Fully phased haplotypes allow the precise characterization of complex structural variation. SVs, such as large inversions, are increasingly recognized as important drivers of adaptation [43], phenotypic evolution [44], and recombination landscape heterogeneity [45], but their detection and interpretation require high–quality, often phased reference genomes [7, 8, 37, 46]. In barn owls, previous indirect evidence suggested the presence of a large inversion linked to climate [47], but its structure and extent could not be determined with pseudo–haploid or fragmented references [32, 33]. Our phased assemblies reveal a ∼38 Mb complex inversion on chromosome 7, comprising multiple nested inverted and collinear segments. Yet the evolutionary history and potential phenotypic consequences of this inversion remain unknown. An important next step will be to examine its population frequency, heterozygosity patterns, and possible associations with traits such as colouration, dispersal, or behaviour traits that have shown geographic structure in barn owls [32].

### Conclusions and perspectives

Together, our phased, chromosome–level assemblies provide a high-quality genomic resource for the barn owl and contribute to the growing set of genomic resources available for the order Strigiformes [30, 32, 33]. They reveal previously unknown chromosomes, expose the architecture of a complex chromosomal inversion, and shed new light on microchromosome characteristics. These resources create a strong foundation for investigating structural variation, recombination dynamics, and the genetic basis of phenotypic divergence in barn owls. When combined with long-term ecological and population-genomic datasets available for this species, they will enable new levels of biological inference and contribute to a broader understanding of avian genome evolution.

## Material and methods

### Sampling

To build our genome assemblies, we used the same reference individual as in Machado et al. (2022) [32], a Swiss male barn owl (M040663), and its 2 parents (father ring ID: M032330; mother ring ID: M028977). On the 9 September 2019, we collected brachial-vein blood from each bird at a nestbox near Estavayer-le-Lac, Switzerland (46.85 N, 6.86 E). Blood samples were either kept at 4°C and processed rapidly for high molecular weight (HMW) DNA extraction with agarose plugs (see below) or snap-frozen in liquid nitrogen and transferred to −80°C freezer for long-term preservation.

### HMW DNA extraction of the reference individual

HMW DNA for the reference individual was extracted using an agarose plug method based on Zhang et al. [48] and previously described in Machado et al. [32]: fresh phosphate–buffered saline (PBS)-washed red blood cells were embedded in low-melt agarose plugs, digested with proteinase K, washed in EDTA, then in 10 mM Tris pH 8.0, 1 mM EDTA, melted, and digested with β-agarase to release the DNA. The resulting HMW DNA was dialysed in 10 mM Tris pH 8.0, kept at 4°C, gently homogenized, quantified with Qubit (Thermo Scientific), and sized on Femto Pulse and pulsed-field gel electrophoresis. Mean fragment DNA size was around 100–150 kbp. This DNA was used for PacBio HiFi SMRTbell Express 2.0 and for one ONT library.

To increase DNA yield, we extracted new HMW DNA from the −80°C frozen blood using MagAttract kit (Qiagen, elution repeated up to 4x to get enough DNA) for future PacBio library preparation. Frozen blood was also used to prepare HMW DNA with the Monarch® HMW DNA Extraction for Tissue kit (New England Biolabs) for future ONT libraries.

### Library preparation and sequencing

#### PacBio HiFi

The PacBio HiFi SMRTbell libraries were prepared and sequenced at the Lausanne Genome Technologies Facility (GTF, University of Lausanne). Briefly, HMW DNA was sheared (Megaruptor; Diagenode, Denville, NJ, USA) to ∼20 kbp fragments and checked on a Fragment Analyser (Advanced Analytical Technologies, Ames, IA, USA). We prepared 3 SMRTbell libraries, 2 with SMRTbell Express 3.0 (10 µg DNA each) and 1 with Express 2.0 (5 µg), in accordance with the manufacturer’s recommendations. After BluePippin size selection (>15 kbp; Sage Science, Inc., Beverly, MA, USA), libraries were sequenced on one and 2 SMRT 8M cells, respectively, using v2.2/v2.0 chemistry on a PacBio Sequel II instrument (Pacific Biosciences, Menlo Park, CA, USA) for a film run time of 30 h and a pre-extension time of 120 min.

#### Oxford Nanopore Technology

Libraries were prepared from Monarch-HMW and plug-derived DNA using the ligation sequencing kit (SQK-LSK114). Sequencing was performed on PromethION flow cells R10 (FLO-PRO114M) using a PromethION 2 Solo instrument (Oxford Nanopore Technologies, Oxford, UK) at the Gene Expression Core Facility (GECF, EPFL, Switzerland).

For Monarch-HMW DNA, sequencing and base calling were carried out using MinKNOW software (v23.07.5) with the integrated Guppy base caller (v7.0.9) employing the super-accuracy (SUP) model. Plug-derived DNA libraries were sequenced using MinKNOW software (v25.03.9), and base calling was performed with Dorado (v1.0.0) using the SUP model, including duplex read analysis.

### Hi-C crosslinking

Without thawing, we diluted 10 µl of −80°C frozen blood from the focal individual in 926.9 µl of PBS and mixed gently. We then added 63.1 µl of 10% formaldehyde and incubated for 20 min at room temperature (mixing periodically). We quenched with glycine (final concentration of 125 mM), followed by a 15–min incubation at room temperature. We stored the sample at −80°C and sent frozen to Phase Genomics (USA) for Hi–C library preparation. Sequencing was performed at the GTF (University of Lausanne, Switzerland).

### DNA extraction and sequencing of the parents

Following Cumer et al. [49], we extracted genomic DNA from whole blood with the DNeasy Blood & Tissue kit (Qiagen, Hilden, Germany). We prepared Nextera DNA libraries (Illumina) and sequenced multiplexed libraries on a NovaSeq 6000 (PE high throughput) at the GTF (University of Lausanne, Switzerland). This sequencing of the parents yielded to a total of 172,731,951 and 93,360,939 read pairs for the father and the mother, respectively.

### Initial trio assembly

Taking advantage of trio information, we used hifiasm v0.19.8-r603 in trio mode to generate phased haplotype assemblies. Parental *k*-mers were first computed using yak (version 0.1-r93-dirty). Because parental sequencing depth was uneven (∼38× for the father and ∼21× for the mother, for an estimated genome size of ∼1.3 Gb), we followed developer recommendations and relaxed the lower count threshold for the maternal dataset (yak option -l2) to retain informative low-frequency *k*-mers.

We then ran hifiasm using all PacBio HiFi reads, together with Oxford Nanopore long reads (>25 kbp), and combined with the 2 parental *k*-mer databases. To improve the resolution of repetitive regions, we increased the *k*-mer frequency cutoff parameter to -D 10. This more permissive threshold prevents over-filtering of moderately repetitive but informative sequences and improving graph connectivity during assembly.

This procedure resulted in a highly unbalanced phased assembly, with a paternal assembly of 1,755,937,883 bp (GC = 46.3%) and a maternal assembly of 1,207,411,275 bp (GC = 42.7%).

Unequal haplotype representation in hifiasm assemblies is a known issue that can arise when the homozygous coverage is incorrectly inferred, in which case the –hom-cov parameter can be adjusted to better separate homozygous and heterozygous *k*-mer peaks. However, in our dataset, the inferred homozygous coverage (∼119×) closely matches the expected value based on the *k*-mer spectrum, with a clear and well-separated heterozygous peak (∼58×), indicating that coverage misestimation is unlikely to explain the observed imbalance.

We therefore hypothesized that the imbalance instead originates from an excess of highly repetitive reads, which can bias overlap selection and graph construction, ultimately leading to uneven haplotype resolution. To address this, we implemented an additional filtering step in which reads were stratified based on repetitiveness and highly repetitive reads were selectively removed prior to reassembly.

### Identification of repeated sequences and HiFi read classification

To reduce assembly artefacts from extreme repeats, i.e., reads composed almost entirely of highly repetitive sequences, we first counted 64–mers in HiFi reads with Jellyfish v2.3.0 (-m 64 -C -c 3 -t 32 -L 50 -s 9,717,986). There were 1,793,627 64–mers occurring >1,000x. These 64-mers were offsets of the same patterns and carried several instances of smaller sub-motifs. For example, the 6 most frequent *k*–mers (each, >1.5 M) carried several instances of the sub–motif GTCACGTGGTC. The next 4 (each >1.3 M) carried TTAGGGTTAGGGTTAGGG (further categorized as telomeric repeats). We further inspected reads containing highly repeated motifs and noticed some were covered by multiple instances of identical sub-motifs, which we decided to filter prior to the assembly. Based on these observations, we placed HiFi reads into 5 categories based on the presence of the following motifs scanned in forward and reverse complement orientation:

- *Centromere–like*, characterized by at least 1 occurrence of the sequence.

CAGCACTGTTTCCTCCAAACTACCATGCTGCGCATAGCAATCCCTGTCTAGCTGCTTCCTTTCACTGGGAACACA, and labelled as *centromeric-like* due to the high conservation of a 162pb long repeat, compatible with a centromeric sequence [50]. This classification was further confirmed by the FISH experiment; see the *Karyotyping and FISH of telomeric and centromeric sequences* section below.

- Kinetochore–like/pericentromeric repeat, characterized by at least 3 occurrences of the sequence of the motif GTCACGTGGTC, labelled as such since these repeats are frequently found at extremities of reads composed of centromere-like repeats.
- Telomere–like, characterized by at least 3 occurrences of the motif TTAGGGTTAGGGTTAGGG, composed of multiple repeats of the vertebrate telomeric motif (TTAGGG) [51].
- Microsatellite, characterized by at least 1 occurrence of the sequence.

ACCTTCCCCCCACCCCTCCCTTCTTCCCGGGCTCAGCTCTGCTCCCGATATCTCTACCTCCTCC and labelled as *microsatellite* since this sequence has been previously described and was used to genotype microsatellite markers in the barn owl [52].

- Other (none of the above).

We excluded centromere–like and kinetochore–like reads since they contained repeated motifs spanning entire reads of length over 20 kbp but retained telomere–like (since they are at the end of chromosomes and less likely to confuse the assembler), and microsatellite–like whose repeated motif was present only in parts of the reads. This yielded a total of 7,826,051 retained HiFi reads that were subsequently sorted by parental allele (see next section).

### Trio assembly excluding centromeric-like and kinetochore-like sequences

Based on this classification, we performed a second assembly using hifiasm v0.19.8-r603 in trio mode with the same parameters as described above in “*Initial trio assembly*,” but excluding reads classified as centromeric-like and kinetochore-like. The assembly was conducted using the filtered set of HiFi reads, filtered Oxford Nanopore long reads (>25 kbp), and parental *k*-mer databases.

This procedure resulted in a smaller yet still unbalanced phased assembly, with a paternal assembly of 1,474,597,196 bp (GC = 41.7%) and a maternal assembly of 1,196,923,645 bp (GC = 42.1%). Notably, the GC content between haplotypes became more consistent, suggesting that the removal of highly repetitive reads reduced sequence composition biases associated with repeat-rich regions.

In hifiasm trio mode, reads are internally partitioned into parental haplotypes based on *k*-mer matches derived from parental sequencing data. While this approach is generally effective, its accuracy may remain limited in moderately repetitive sequence and low-divergence allelic regions that can still share *k*-mers between haplotypes, reducing the discriminative power of *k*-mer–based assignment.

In addition, the unequal sequencing depth between parental datasets (∼38× vs. ∼21×) may lead to asymmetries in parental *k*-mer representation, potentially biasing read assignment towards the more deeply sequenced parent despite the use of relaxed filtering parameters.

Thus, given the persistence of haplotype imbalance after repeat filtering, we hypothesized that residual misassignment or ambiguity in read partitioning affect assembly quality. To further improve haplotype resolution, we therefore explicitly partitioned HiFi reads by parental origin prior to assembly and performed independent assemblies for each haplotype.

### Allele sorting of HiFi reads

With the final intention to build haplome-specific assembly, we sorted HiFi reads according to the parent of origin using a *k*-mer–based approach that identifies parent-specific kmers in each read.

We first build parent-specific sets of *k*-mers from parental Illumina reads. To balance uneven parental sequencing depths (Father 172′731′951 reads vs. Mother 93′360′939 reads) and sampling bias of kmers during sequencing, we randomly drew non-overlapping subsets of 46,680,469 Illumina paired-end reads, 3 for the Father and 2 for the Mother. In each of these 5 sets, we counted 49-mers (length chosen to avoid palindromes and reduce homopolymer errors; Supplementary Table 4), free of Ns. Finally, we defined 9 parent-specific *k*-mer libraries containing 49-mers present ≥2x within a parental subset and absent from pairs of subsets of the other parent (see Supplementary Table 5). These parent-specific *k*-mer libraries allowed to account for sampling bias and statistical testing during the read classification procedure described in the next paragraph. This comparison between parental sets was done using a modified jellyfish v2.3.0 to emit *k*-mer “bin” files unique to one library and absent from a given set of other libraries (patch provided on the GitHub, see the Availability of Source Code and Requirements section for details.

We sorted the HiFi reads of the reference individual into 2 parental sets. Each HiFi read was scanned with a sliding window of 49–mers, using a 7–nt step to keep the computational burden manageable. For each read, we counted the occurrences of parent–specific *k*–mers. This counting procedure was performed for every pairwise combination of the parent–specific *k*–mer libraries, generating a distribution of *k*–mer counts for each parent for each read. Based on these distributions of counts, we assigned reads to parental origin using paired *t*–tests with Benjamini–Hochberg FDR control implemented in R. Reads with FDR ≤ 0.001 were assigned to the parent with the highest number of matching *k*–mers. Unassigned reads were rescanned using a 1–nt step sliding window, and the assignment test was repeated using an FDR threshold of ≤0.05. FDR control was performed separately within each read class (telomere–like, microsatellite–like, other) to account for compositional biases associated with these sequence categories (Supplementary Table 6).

### ONT reads filtering, classification, and allele sorting

For ONT reads, we selected all reads generated by the ONT caller that were 25 kbp nucleotides or longer. ONT reads were then attributed to the 5 categories described earlier (*Centromere–like, Kinetochore–like/pericentromeric repeat, Telomere–like, Microsatellite, other*) using the motifs identified with HiFi reads. The allelic sorting process explained in the previous section was applied to the ONT runs 1 and 2 (≥25-kbp long) using a sliding window offset of 7 nt and an adjusted *P*-value ≤ 0.05 to assign predicted parental origin. Detailed results after the filtering, classification and sorting are presented in Supplementary Table 7.

### Haplotype-specific assembly and scaffolding

#### HiFi-based assemblies

We ran hifiasm 0.19.8-r603 to build separate maternal and paternal haplotype using (i) HiFi reads assigned to that parent, (ii) all HiFi reads from uncertain origins, and (iii) ONT from run 1 (ONT run 2 not available at that stage). After the initial assemblies and to detect regions with abnormally high parental coverage, we realigned HiFi reads to the draft assemblies with minimap2 2.26-r1175. After realigning the reads to both parental haplotypes, we assigned each read to the parent with the highest alignment score. After that step, we identified regions where the coverage was ≤20 and ≥80% to the expected coverage. Reads contributing to these imbalances were identified, sorted by increasing adjusted *P*-value from the read sorting step (see above for details) and the bottom half of them (i.e., worse adjusted *P*-values) re-assigned to the other parental allele, implementing the idea that the increased coverage is due to wrongly assigned reads. After this final reclassification of 14,717 reads, we generated 2 final haplotype assemblies using hifiasm 0.19.8-r603. The final drafts comprised 287 paternal and 331 maternal unitigs.

#### ONT assemblies

Using hifiasm 0.25.0-r726, we independently assembled ONT reads into parental haplotype (inputs: ONT runs 1 and 2 assigned to that parent + uncertain-origin ONT reads from runs 1 and 2), yielding 1,030 paternal and 985 maternal unitigs.

#### Final scaffolding with linkage groups and ONT-based assemblies

We first scaffolded the HiFi-based contigs into 40 linkage groups using the linkage map produced by Topaloudis et al. [34]: we extracted ±100 bp around each marker from the old assembly to create pseudo-reads and mapped them to the new haplotype assemblies, thus ordering unitigs by marker order and coordinates.

Then, we mapped all HiFi reads to their HiFi unitigs to flag low-coverage regions of interest (ROI). Using ad hoc scripts and aligners (minimap2, LASTZ, MAFFT), we stitched HiFi and ONT unitigs across ROIs and unitig ends guided by the linkage map, to produce the final scaffolds for both haplotypes. Stitched scaffolds were spaced by stretches of 10′000 Ns. Because owl chromosomes are acrocentric (Fig. 2B) [31, 53], and we excluded centromere-rich reads, we oriented all scaffolds with a 5′ telomere (e.g., 5p-CCCTAA-3p repeat) at the p arm so that adding centromeres later will not alter the coordinate system.

### Recovery of micro-chromosomes

#### Identification of the microchromosomes

To recover the unscaffolded microchromosomes in both assemblies, we used MicroFinder [24]. This tool compares the gene content of unplaced scaffolds against a curated set of genes known to be enriched on microchromosomes across avian species, flagging candidates showing significant enrichment. Following the authors’ recommendations, we set a maximum scaffold size cutoff of 5 Mb to focus the analysis on short, genuinely unplaced sequences and retrieved 14 and 18 micro-chromosome contigs for the paternal and the maternal assemblies, respectively.

#### Assembly of the microchromosomes

Candidate unitigs identified in the *Recovery of microchromosomes* section were then manually assembled into 6 additional microchromosomes. This manual curation was done using ad hoc scripts and aligners (minimap2, LASTZ, MAFFT) and supported by sequence alignment comparisons between the maternal and paternal haplotypes as well as using the unitigs produced by HiFi and ONT assemblies. Stitched unitigs were spaced by stretches of at least 10 kbp of Ns.

### Detection of terminal features of the chromosomes

We assessed whether chromosomes were assembled completely (i.e., spanning telomere–to–centromere for autosomes or telomere–to–telomere for the Z chromosome). Because centromeric and pericentromeric reads were excluded during assembly, we realigned the raw long-read data to both the maternal and paternal assemblies to identify reads mapping to chromosome ends and containing telomeric or centromeric/pericentromeric repeats. Both PacBio HiFi (Sequel IIe, chemistry M64) and ONT long reads were mapped against each reference. Each read was then assigned to a genomic feature class (telomere, centromere, kinetochore, microsatellite repeat, or “other”) based on the presence of class–specific sequence motifs (see Identification of repeated sequences and HiFi reads classification). Only primary alignments were retained (filtering on tp:A:P), and a per–read mapping proportion was computed as the number of matched bases divided by the total read length.

Reads mapping near chromosome termini (where telomeric and centromeric repeats are expected) were identified by selecting those with alignment start positions within 10 kbp of the chromosome beginning or alignment end positions within 10 kbp of the chromosome end. Terminal–proximal reads were further filtered to retain only those with mapping quality >10 and mapping proportion >5%. Finally, reads not classified as telomeric, centromeric, or kinetochore-like were removed.

Finally, reads were grouped by parental haplotype, chromosome, terminus (start or end), and feature class. Clusters supported by fewer than 15 reads were discarded. Because all scaffolds were oriented based on the presence of telomere–specific repeats (e.g., 5′–CCCTAA–3′), we then examined the distribution of read clusters along each chromosome. Specifically, we inspected autosomes for clusters of telomeric reads at their chromosome starts, and the Z chromosome for telomeric clusters at both termini. Conversely, we assessed chromosome ends for the presence of centromeric or pericentromeric read clusters, consistent with expectations for scaffold boundaries corresponding to centromeric regions.

### Protein-based synteny across avian species

To confirm the identification of the Z chromosome, we aligned chicken (GCF_016699485.2) and zebra finch (GCF_048771995.1) proteins to both haplotype assemblies using miniprot (v0.18-r281) [54]. We kept alignments with ≥70% sequence identity and ≥90% target coverage, then merged pairwise mapping formats (PAFs) with reference feature tables to count matches per reference chromosome vs. barn owl chromosome. Sankey diagrams summarized chromosome-level correspondences (Supplementary Figs 1 and 2).

### Evaluation of the phasing of the assemblies

To avoid biases caused by differences in parental sequencing depth, we down-sampled parental Illumina reads to 80,000,000 per parent using seqtk (v1.4) with a fixed seed (-s 100) for reproducibility and counted 49–mers with meryl *count* (v1.4.1). We derived parental–private *k*–mers (meryl *difference*), counted 49-mers in each assembly, and computed a phasing error as the number of misplaced parental-private *k*-mer in the wrong haplotype divided by the total number of parental-private *k*-mers across both haplotypes.

### Genome quality control

Quality and completeness were evaluated at 2 stages of the assembly process: the contig level (prior to scaffolding) and at the chromosome level of the final parental assemblies. At both stages, we assessed completeness with BUSCO v6.0.0 (aves_odb12) [55] and used Quast v5.3.0 [56] to compute the general assembly statistics (sequences counts, N50/N90, GC, gaps). Assembly completeness and contiguity were visualized with BlobTools2 snailplots [35].

Genome contamination was also evaluated at the contig level for the parental assemblies using BlobToolKit v4.5.3 [35]. HiFi reads were aligned to the maternal and paternal assembly FASTA sequences using minimap2 [57] (v2.24-r1122) to generate BAM files for coverage estimation. Taxonomic assignments were obtained by aligning assembly FASTA sequences against the NCBI nucleotide (nt) database (July 2021 release) using BLASTN [58] (v2.14.0; parameters: -outfmt '6 qseqid staxids bitscore std' -max_target_seqs 10 -max_hsps 1 -evalue 1e-25). Blob plots were generated using species-level taxonomic assignments (bestsumorder_species) and sequence coverage information. The resulting plots were visually inspected for contigs with taxonomic assignments inconsistent with birds, which could indicate potential contamination. Finally, whole-genome synteny was assessed by aligning the maternal haplome to the paternal haplome using minimap2 (-x asm10) [57].

### Repeat annotation

We identified the repeats with 2 complementary approaches: (i) Red (de novo, library free) [59] and (ii) RepeatModeler v2.0.4 [60] with default parameters to build a custom repeat library, followed by RepeatMasker v4.1.5 [61] with default parameters for classification and masking of the repeats.

### Gene annotation

We annotated both haplomes with NCBI Eukaryotic Genome Annotation Pipeline (EGAPx) [62]. In brief, EGAPx takes a genome assembly in FASTA format, a taxonomy ID, and RNA-seq data as input. First, it uses protein homology by aligning protein sequences from closely related species with prosplign. Second, it uses transcriptome information from the focal species. We included RNA-seq short reads from 6 tissues (liver, heart, kidney, testis, growing back feathers, and thalamus) previously published in Ducrest et al. [33], which EGAPx aligns with STAR [63] and minimap2 [57]. Third, EGAPx runs Gnomon to generate *ab initio* predictions [64]. These different hits are then integrated by Gnomon to produce a final consensus annotation.

We realigned CDS from the EGAPx annotation to their respective haplome with minimap2 in split mode [57] and kept only full-length mapping, MAPQ = 60. These quality-filtered CDS were then mapped back to the previous reference genome (T.alba_DEE_v4.0–GCF_018691265.1) to classify CDS as previously present (full-length, MAPQ = 60) or new. For multi-mapped CDS, we retained the alignment with the longest aligned span and summarized counts per chromosome.

### Chromosome characteristics and classification

To distinguish macro-, micro-, and dot chromosomes, we ran a PCA on *z*-scored, chromosome-level features: (i) length, (ii) total exonic length (relative), (iii) GC, (iv) gene density, (v) exon density, (vi) mean exon length; (vii) fraction of simple/low complexity repeats; and (viii) fraction of other RepeatMasker classes (resolving overlaps by the larger element’s class).

### Genome mappability masking

Genome-wide mappability was quantified using the seqbility/SNPable pipeline (see the Availability of Source Code and Requirements section for details). The reference genome was first decomposed into all possible *k*-mers using the splitfa program, with the *k*-mer size of 150 bp, matching common read length. These *k*-mers were then aligned back to the reference using BWA in aln mode [65]. To match the SNPable specification, we used several non–default BWA parameters to allow permissive and sensitive mapping: the seed length was reduced (-l 32), up to 2 seed mismatches were permitted (-k 2), and both the gap–open (-O 3) and gap–extension (-E 3) penalties were increased relative to standard BWA defaults.

The resulting SAM file was processed with gen_raw_mask.pl, which extracts, for each *k*-mer, the number of perfect and near–perfect alignments it produces across the genome. This raw mask was then summarized into position–wise mappability tracks using the gen_mask script. For this step several non–default stringency thresholds were applied (-r 0.50, 0.90, 0.95, and 0.99). These thresholds represent the minimum fraction of overlapping *k*-mers that must map uniquely for a genomic position to be considered mappable. Lower thresholds generate more permissive callable regions, whereas higher thresholds identify only the most confidently unique regions.

Finally, we quantified the proportion of mappable regions per chromosome at different stringency thresholds (0.50, 0.90, 0.95, and 0.99), calculated as the ratio of the cumulative length of mappable regions to the total chromosome length.

### Recombination map

We lifted the coordinates of the linkage markers from [34] by extracting 200-bp around each SNP with bedtools *getfasta* (v2.x) and mapping these sequences to each haplome (bwa–mem v0.7.x, sorted with samtools v1.x). We kept only SNPs with unique and perfect alignments (MAPQ = 60), and for each combined the new physical coordinates with the original linkage–map metadata (linkage group and genetic position) to lift the recombination maps coordinates in each haplotype coordinate system.

### Hi-C contact maps

We sequenced 338,385,433 Hi-C pairs. After enzyme-site filtering for GATC at R1 ends and R2 starts, 112,239,344 paired reads (33.2%) remained. We aligned reads with FetchGWI [66], converted alignments to genomic pairs, and built .cool files with cooler v0.9.3 (cooler *cload pairs* c1 1 -p1 2 -c2 3 -p2 4), followed by cooler *zoomify*. Genome-wide maps were generated at 16-kbp wide bins.

### Karyotyping and FISH of telomeric and centromeric sequences

#### Cell culture and chromosome preparation

On the 10 July 2025, we collected 3 growing breast feathers from a 28-day-old female nestling (M046151). After surface sterilization (70% ethanol) and PBS rinses, we dissected the feather basal parts, scraped inner tissues using tweezers, added it to a 24-well plate containing Dulbecco’s Modified Eagle’s Medium high glucose (DMEM, Avantor) with 20% foetal bovine serum (FBS, Sigma), 1% penicillin–streptomycin (Thermo Scientific), 5% AmnioMax II (Gibco, Thermo Scientific) and the MycoGenie MycoPlasma Elimination Kit (1:2000; Assay Genie, Chemie Brunschwig). Cultures were maintained at 37°C with 5% CO_2_ for ≥3 weeks to obtain enough cells.

To arrest mitosis, we treated cells with KaryoMax Colcemid (0.5 µg/ml, 1 h; Thermo Scientific), followed by 0.075 M KCl hypotonic shock (20 min, 37°C) and gentle fixation in cold freshly prepared methanol/acetic acid (3:1) with 3–4 wash/fix cycles. The metaphase chromosome suspensions were stored at −20°C.

#### Hybridization

We spread 10 µl of metaphase suspension per slide. After aging (1 h, 65°C) and a 2x SSC wash (10 min, 300 mM sodium chloride, 30 mM sodium citrate, pH 7.0) at room temperature, slides were incubated with 100 µg/ml RNAse A (Qiagen) in 2x SSC for 45 min at 37°C. The slides were then washed in 2x SSC for 5 min at room temperature and treated with 0.005% pepsin for 3 min in 0.01 N HCl heated to 37°C. We then washed in PBS with 50 mM MgCl₂, denatured them in 1% paraformaldehyde (Thermo Scientific) for 10 min at room temperature, washed with 2x SSC, and dehydrated using a series of 70%, 90%, and 100% ethanol and let them dry for 20 min at room temperature. We hybridized the slides with 1 pmol of CEN3.2_ATTO590 centromeric probe (5′-TTTCCTCCAAACTACCATGCTGCG-3′ labelled with ATTO590 at the 5′ end, see the *Centromere–like* section above for the identification of this sequence) in 20 μl containing the PNA telomere hybridization buffer with 10 μl of Telomere PNA FITC probe (Telomere PNA Kit/FITC for Flow Cytometry, PNA FISH Kit/FITC, Agilent Technologies). Coverslips were sealed on the slides, the slides denatured (92°C, 3 min) and hybridized overnight (37°C). The slides were then washed twice in 2x SSC + 0.1% Tween 20 (60°C, 15 min), and once in 0.2x SSC (room temperature), then mounted with Vectashield mounting medium + DAPI (500 ng/ml; Sigma). Imaging used a Leica Stellaris5 confocal microscope equipped with a 63xPlanApo oil immersion objective (CIF platform, University of Lausanne). All acquisitions were produced by superimposing focal planes. Post-processing, including pseudo colouring, was performed using Fiji [67].

## Additional files

**Supplementary Figure 1**. Miniprot alignments for the paternal proteome. Protein alignments of the chicken and zebra finch proteome against the paternal haplome (only matches with ≥70% sequence identity and ≥90% target coverage retained). Each block represents a sequence in the assembly, and connecting lines indicate protein matches.

**Supplementary Figure 2**. Miniprot alignments for the maternal proteome. Protein alignments of the chicken and zebra finch proteome against the maternal haplome (only matches with ≥ 70% sequence identity and ≥ 90% target coverage retained). Each block represents a sequence in the assembly, and connecting lines indicate protein matches.

**Supplementary Figure 3**. Contamination analysis of the paternal contigs. Each circle corresponds to a single paternal contig, plotted according to its GC content (*x*-axis), and HiFi sequencing coverage (*y*-axis). Circle size is proportional to the true contig length (bp), with larger circles indicating longer contigs. Colours denote the taxonomic origin of the contig based on the most frequent BLAST hit, as specified in the *species* legend. Contigs labelled as “no-hit” correspond to sequences without significant BLAST matches.

**Supplementary Figure 4**. Contamination analysis of the maternal contigs. Each circle corresponds to a single maternal contig, plotted according to its GC content (*x*-axis), and HiFi sequencing coverage (*y*-axis). Circle size is proportional to the true contig length (bp), with larger circles indicating longer contigs. Colours denote the taxonomic origin of the contig based on the most frequent BLAST hit, as specified in the *species* legend. Contigs labelled as “no-hit” correspond to sequences without significant BLAST matches.

**Supplementary Figure 5**. Repeat landscape of the paternal and maternal haplomes. Proportions of repeat classes annotated with RepeatModeler and classified with RepeatMasker are shown for each parental haplome. Percentages are expressed relative to the entire repeat content of the corresponding haplome. It should be noted that the centromeric and pericentromeric repeats were not analysed here.

**Supplementary Figure 6**. PCA of chromosome-level features (*z*-scored). Principal component analysis was performed on the following features: (i) length, (ii) total exonic length (relative), (iii) GC content, (iv) gene density, (v) exon density, (vi) mean exon length, (vii) fraction of simple/low complexity repeats, and (viii) fraction of other RepeatMasker classes (overlaps resolved by the larger element’s class). Paternal chromosomes are shown in the left panel and maternal chromosomes in the right panel; chromosomes absent from the previous reference (GCF_018691265.1) are highlighted in lighter colours in both panels.

**Supplementary Figure 7**. Fluorescence *in situ* hybridization (FISH) visualization of the *Tyto alba* karyotype of a female individual. Chromosomes are counterstained with DAPI (blue). Centromeric regions are highlighted in magenta and telomeric regions in cyan using specific DNA probes (see the Material and methods section for details). Sexual chromosomes Z and W are indicated by white arrows.

**Supplementary Figure 8**. Mappability of the different chromosomes for the 2 parental haplomes. Paternal haplome is displayed on the upper panel, and the maternal haplome on the lower panel. Different colours correspond to different stringency thresholds used while measuring mappability.

**Supplementary Table 1**. Summary of sequencing libraries. PacBio HiFi and ONT libraries received from the Lausanne Genomic Technologies Facility (GTF) and the Gene Expression Core Facility (GECF), respectively, with the corresponding read count and mean read length (bp) for each library. In this table, the 5 HiFi libraries are listed first, followed by the 2 ONT libraries.

**Supplementary Table 2**. Chromosome-level features of both haplomes. Are listed (i) length, (ii) total exonic length (relative), (iii) GC, (iv) gene density, (v) exon density, (vi) mean exon length, (vii) fraction of simple/low complexity repeats, and (viii) fraction of other RepeatMasker classes (resolving overlaps by the larger element’s class).

**Supplementary Table 3**. Terminal features per chromosome. For each parental chromosome, presence/absence of telomeric and pericentromeric sequences and the corresponding span (bp).

**Supplementary Table 4**. Counts and composition of parental 49-mer libraries. For each parental 49-mer library (3 paternal, 2 maternal), we report the number of distinct 49-mers, the number of unique 49-mers, the total 49-mer observations, and the maximum per-49-mer copy count.

**Supplementary Table 5**. Parent-private 49-mers. Paternal and maternal 49-mer sets used identify parent-specific sequences. 49mers contain no Ns and are considered parent-private when present ≥2× in a subset of one parent and absent from subsets of the other parent.

**Supplementary Table 6**. HiFi reads parental assignment by category. The number of paternal, maternal, and unclassified reads for each of the read categories determined by their repeat profile (see the *Identification of repeated sequences and read filtering* section for details).

**Supplementary Table 7**. ONT (≥25 kbp) reads parental assignment by category. The number of paternal, maternal, and unclassified reads for each of the read categories determined by their repeat profile (see the *Identification of repeated sequences and read filtering* section for details).

## Availability of source code and requirements

Project name: TytoAlba_NewReferenceGenome

Project home page: https://github.com/hugocrvl/TytoAlba_NewReferenceGenome

License: GNU GPL 3 license

Operating system(s): Linux

Programming language: Perl, Shell, R, Jupyter Notebook, Python

## List of abbreviations

b: bases; BAM: Binary Alignment Map; bp: base pair; BLAST: Basic Local Alignment Search Tool; BUSCO: Benchmarking Universal Single-Copy Orthologs; CDS: Coding DNA Sequences; FDR: false discovery rate; FISH: fluorescence *in situ* hybridization; Gb: gigabases; Hi-C: high-throughput chromosome conformation capture; HiFi: high-fidelity; HMW: high molecular weight; k: kilo; LINE: long interspersed nuclear element; LTR: long terminal repeat transposable element; NCBI EGAP: National Center for Biotechnology Information Eukaryotic Genome Annotation Pipeline; ONT: Oxford Nanopore Technologies; PCA: principal component analysis; T2T: telomere-to-telomere.

## Supplementary Material

giag092_Supplemental_Files

giag092_Authors_Response_To_Reviewer_Comments_original_submission

giag092_GIGA-D-26-00095_original_submission

giag092_GIGA-D-26-00095_revision_1

giag092_Reviewer_1_Report_original_submission Reviewer 1 -- 4/9/2026

giag092_Reviewer_1_Report_revision_1 Reviewer 1 -- 7/28/2026

giag092_Reviewer_2_Report_original_submission Reviewer 2 -- 5/4/2026

giag092_Reviewer_2_Report_revision_1 Reviewer 2 -- 7/20/2026

## Data Availability

Assemblies are available under the Umbrella BioProject PRJNA1444161 (paternal assembly: PRJNA1440222, maternal assembly: PRJNA1440227). The data underlying this article are available under the BioProject PRJNA1444114. FASTA sequences, repeat annotations, gene annotations, recombination maps, and mappability masks for both haplotypes are available on Zenodo [68]. The source data underlying Figs 1 –4 and Supplementary Figs S1–S8 have also been deposited on Zenodo [69].
