# Supplementary material for: A chromosome-level, haplotype-resolved genome assembly for the barn owl, Tyto alba
Source: Gigascience. 2026 Sep 8;15:giag092. doi: 10.1093/gigascience/giag092 (PMC13615541; doi:10.1093/gigascience/giag092)
Supplement: Supplementary file 1 [file giag092_authors_response_to_reviewer_comments_original_submission.pdf]

Author's Response To Reviewer Comments

Reviewer #1: General comments
It is always good that high quality genome assemblies are created and made available to the community. However, I have some comments below.

[R] We thank the reviewer for this positive feedback and constructive comments on the manuscript. Please find below a detailed answer for each of the comments and suggestions you raised.

The authors have used a complicated assembly without justifying why this was done. One of the consequences of this was that the reads coming from the centromeres were excluded from the assembly, in addition to other reads. Instead of assembling the (HiFi) reads with hifiasm (or Verkko) and supplying k-mers from parents as is usually done for trio-assemblies, the authors separated reads before assembly. Both hifiasm versions used by the authors have had the ability to utilize HiFi and ONT reads at the same time (https://github.com/chhylp123/hifiasm?tab=readme-ov-file#ultra-long-ont-integration, the last example is directly applicable here). Why that approach, which has been used multiple times in the literature, was not chosen, and a custom approach was used instead is not clear to me. The authors should at least have demonstrated that the custom approach was superior to the "standard" approach, thereby justifying why it was done.

[R] We thank the reviewer for raising this important point.
We agree that hifiasm natively supports the joint integration of HiFi reads, ONT ultra-long reads, and parental k-mer information, and that this “standard” approach has been successfully used in multiple studies.
In our study, we initially followed this standard strategy by running hifiasm in trio mode with all HiFi and ONT reads simultaneously, using parental k-mer databases as recommended. However, this approach consistently led to highly unbalanced phased assemblies, with a large discrepancy in haplotype sizes (1.76 Gb for the paternal haplotype versus 1.21 Gb for the maternal haplotype), which deviates substantially from the expected genome size (~1.3 Gb) and from each other. In addition, we observed inconsistencies in assembly quality metrics (e.g., BUSCO completeness and duplication rates) and suboptimal phasing performance.
We first investigated whether this imbalance could be attributed to incorrect estimation of homozygous coverage, a known issue in hifiasm that can be mitigated using the --hom-cov parameter. However, the k-mer spectrum of our dataset showed a well-defined heterozygous peak (~58×) and homozygous peak (~119×) (see attached log file), consistent with expectations and indicating that coverage inference was accurate.
Inspection of the k-mer spectrum and read content revealed a substantial fraction of extremely repetitive reads, including centromeric and pericentromeric sequences spanning entire long reads (>20 kb). Such reads are known to complicate overlap-based assembly by generating large numbers of ambiguous overlaps and by disproportionately influencing graph construction. Based on these observations, we hypothesised that these sequences were biasing the assembly process and contributing to haplotype imbalance.
To address this issue, we implemented a targeted read classification and filtering strategy, in which HiFi reads were stratified according to the presence of highly repeated motifs. Reads dominated by centromeric-like and kinetochore-like repeats were excluded, while other categories with repeated motifs present only in a fraction of the reads (e.g., telomeric and microsatellite-containing reads), less likely to confuse the assembler, were retained. Re-running the assembly with this filtered dataset resulted in improved GC consistency between haplotypes and a reduction in haplotype size discrepancy, although some imbalance persisted.

We then considered the limitations of k-mer–based read partitioning in the presence of moderate repeats, low-divergence allelic regions, and unequal parental sequencing depth (~38× for the male vs ~21× for the female). Even after removing the most extreme repetitive reads, these factors may still affect phasing and assembly performance, leading to the persistent haplotype imbalance.

To overcome these limitations, we developed a custom allele-sorting strategy prior to assembly, based on identification of parent-specific k-mers across multiple subsampled parental datasets to mitigate the large parental sequencing depth imbalance. This approach allowed us to explicitly partition reads by parental origin before assembly and to perform independent haplotype-specific assemblies, thereby reducing ambiguity in read assignment and improving phasing accuracy.
Importantly, this strategy was not used as a replacement for standard hifiasm functionality, but rather as a data-driven refinement motivated by observed assembly artefacts that could not be resolved using existing parameters alone. The full rationale and intermediate results supporting this approach have now been clarified in the revised Materials and Methods section, see line 412 to 417 of the Tracked Changes version of the manuscript.

Finally, we acknowledge that the exclusion of centromeric-like reads results in the absence of complete centromeric sequences in the present assembly. This was a deliberate trade-off to improve overall assembly contiguity and haplotype balance. We are currently developing a complementary analysis specifically aimed at resolving chromosome-level centromeric sequences and architecture, which will be presented in a dedicated future study.

The manuscript does not report contig statistics. As far as I can see, all references to N50 are to scaffolds, and not contigs. The only ones referring to N50 contigs are to an old assembly. Knowing the N50 contig at different stages in the complicated assembly process would help us to understand how the process worked and how much it (potentially) improved the assemblies.

[R] The values reported in the “Hifiasm” columns of Table 1 corresponded to the non-scaffolded contigs immediately with hifiasm. The columns named “Final” corresponded to the number of sequences obtained at the end of the scaffolding procedure. To avoid confusion and improve clarity, the mentioned columns have been renamed “Prior to scaffolding [contigs]”, and “After scaffolding [chromosomes]” respectively.

Several places in the manuscript it is stated that this is the "first haplotype-resolved and chromosome-level genome for Strigiformes". This is not justified well enough, and I point to several other assemblies that might fit this definition.

[R] We agree with the reviewer that the claim of a “first haplotype-resolved and chromosome-level genome for Strigiformes” may be open to interpretation depending on how these criteria are defined. While other assemblies are phased and include scaffolding to the chromosome-level for some chromosomes, in the assemblies we examined, not all chromosomes are consistently resolved. To our knowledge, the assembly presented in this manuscript is the first to also include all micro-chromosomes at this level of completeness. However, we acknowledge that such claims depend on a comprehensive evaluation of all existing assemblies and on the specific definition applied. To avoid potential ambiguity and overstatement, we have therefore revised the manuscript to moderate this claim accordingly.

See Lines 53, 231, 236 and 308

Specific comments:

Line 53: I find it a bit strange that you state that this is "first haplotype-resolved and chromosome-level genome for Strigiformes". Searching at GoaT (https://goat.genomehubs.org/) lists 5 Strigiformes with chromosome level assemblies (Athene noctua, Bubo scandiacus, Strix aluco, Strix nebulosa and Strix uralensis), where Athene noctua, Bubo scandiacus, and Strix aluco are all haplotype resolved. You even refer to the publication of one of these (Baalsrud HT et al 2025). Strix aluco has been on NCBI since September 2023, Athene noctua since February 2025 and Bubo scandiacus since March 2025. I guess you imply that these assemblies are only pseudo-haplotypes, but it is not clear to me.

[R] As previously mentioned, we agree that the claim of a “first haplotype-resolved and chromosome-level genome for Strigiformes” can be subject to interpretation depending on the criteria applied. We thank the reviewer for highlighting these additional assemblies. However, while these genomes are indeed phased and scaffolded to the chromosome level, they do not appear to include all the chromosome. In particular, they retain a substantial number of unplaced scaffolds (e.g., 675 for Athene noctua, 1,070 for Bubo

scandiacus or 161 for Strix aluco), which likely correspond in part to unresolved micro-chromosome sequences.

Nevertheless, we acknowledge that such comparisons depend on methodological differences and definitions of completeness. To avoid ambiguity and potential overstatement, we have therefore removed the claim of being the “first” from the manuscript and revised the text accordingly.

Line 86: It is important to note that the sequence in the micro-chromosomes might be in the assembly, but it is difficult or impossible to distinguish from so-called shrapnel (unplaced sequence, often repeats).

[R] Indeed, micro-chromosomes sequences are often contained in so-called shrapnel, left as unplaced scaffolds in the assembly. We revised the text to add this nuance, see Line 84:

“Because of these properties, many avian genome assemblies contain fewer chromosomes than the actual karyotype, with microchromosomes frequently missing or not fully resolved and left as unplaced scaffolds”

Line 89: I don't understand your comment here "...adoption of these approaches has been uneven across birds…" Firstly, getting the correct data require proper handling of samples (flash-frozen ideally, etc), and successful DNA isolation before sequencing. These steps might be expensive, and not all labs/projects might afford this. You write this as it is a conscious choice to not use ONT data, and I think it is mostly cost and other restrictions (maybe PacBio might be the only choice they have).

[R] We agree that the adoption of these approaches is not a matter of deliberate choice but rather reflects practical constraints. We have revised the text to better reflect these considerations and avoid implying that the uneven adoption is intentional, see Line 89:

“However, the adoption of these approaches across avian genomics has been constrained by practical challenges, including difficulties in obtaining high-quality samples, extracting enough ultra-high molecular DNA and the cost of sequencing. As a result, achieving full and accurate resolution of avian microchromosomes remains a major challenge.”

Figure 1: The resolution of this was a bit low, so hard to read the numbers.

[R] We acknowledge that the resolution of the figures in the submitted PDF was suboptimal. The initial submission was made via bioRxiv and subsequently transferred to GigaScience, without the possibility to include additional high-resolution figure files. We expect this issue to be resolved in the resubmitted version. In the meantime, we have reworked the figures to further improve their clarity. The updated figures are provided as attachments.

Table 1: The N50 and N90 here, what do these refer to? It looks like scaffold metrics, but good to be explicit especially when "# contigs" is right above. How many contigs do you have in the final assemblies? It is written as 46 here, but you also have gaps, so I guess the number is higher. You have 46 chromosomes I guess. You cannot state "# contigs" and report scaffolds/chromosomes. Have a separate line for that or something (or just state it somewhere).

[R] Indeed, N50 and N90 are classical assembly metrics that represent the minimum length of sequences that cover a cumulative 50% or 90% of the entire assembled genome. We clarified these definitions in Table 1. As mentioned in a previous answer, we also revised the table structure. In addition, we replaced the term “# contigs” with “# sequences” to encompass contigs, scaffolds and chromosomes.

Figure 2: This figure is difficult to interpret. Colours are unclear, symbols are unclear (difficult to distinguish Similar and Opposite orientation). Text is diffuse. The green and red circles are really hard to see.

[R] See previous answers to comments on Figure 1 for details. In the meantime, Figure 2 has also been re-worked for clarity and interpretability. More specifically, we changed the colour palette to distinguish between the two haplomes, increased the size of the chromosomes and corresponding telomeres and pericentromeres glyphs. We also modified the legend to make it easier to understand the different

elements of the A panel.

Figure 3: Again, this is hard to see. You need higher resolution. I don't know if this is the fault of the authors or the journal.

[R] See previous answers to comments on Figure 1 for details. In the meantime, Figure 3 has also been re-worked for clarity and readability.

Figure 4: This is really nice. Where are the gaps in these two chromosomes? Does each "arrow" represent a contig?

[R] As indicated in the figure legend, Figure 4 shows an extract of the whole-genome alignment between the paternal and maternal haplotypes, focusing on chromosome 7. Only alignment blocks that are unique to this chromosome in both haplotypes are displayed. The apparent gaps, such as those at the right end of the maternal chromosome 7, correspond to highly repetitive regions (as reflected in the repeat profile below) that align to other parts of the genome and are therefore not shown here. Each arrow represents an alignment block generated by minimap2. All these clarifications have been added to Figure 4 legend.

Line 207: I understand what you want to say, but the T2T era is a consequence of better/longer reads, and not something that in itself has changed genome assembly.

[R] We agree that the T2T era is a consequence rather than a cause. We have revised the text, accordingly, see Line 225:

“Recent advances in long-read sequencing technologies have made it increasingly possible to reconstruct near-complete or telomere-to-telomere chromosomes across an increasing range of species.”

Line 209: "among the most challenging" just depends on what you compare to, and what kind of tools you have available. In my experience, codfishes are awful to assemble with PacBio HiFi, but works excellent with ONT. Likewise here, HiFi leads to incomplete assemblies for birds, but ONT have much better results. For other species/groups, ONT might work really poorly and HiFi much better.

[R] We agree that the difficulty of genome assembly is context-dependent and can vary substantially with the sequencing technology and assembly methods used, as well as across taxa. We have revised the text to avoid overgeneralisation, see Line 228:

“Yet birds remain challenging vertebrates to assemble owing to their numerous, small, GC-rich and repeat dense microchromosomes, which have historically been under represented or mis assembled in reference genomes”

Line 212: I have already protested against the use of "first haplotype resolved, chromosome level assemblies for an owl". You have to define this better, that is, define this assembly such that the species mentioned above is not part of this definition.

[R] We thank the reviewer for this comment and have revised the manuscript accordingly, see previous comments for detailed answer and line 53, 231, 236 and 308 for examples of in text modifications.

Line 234: What do you mean by poor mappability here? We have seen good signals for microchromosomes. See for instance Supplementary Figure 1 here:
https://www.biorxiv.org/content/10.64898/2026.03.26.714473v1.full

[R] Indeed, in the case of Luscinia s. svecica, Hi-C signals performed well, even along microchromosomes. However, our statement refers to a more general context in which microchromosomes are known to be difficult to map due to their high repeat and GC content.

To support this point, we now quantify mappability for each chromosome as the ratio of the cumulative length of mappable regions to the total chromosome length (see M&M for details, Line 678) and included

this analysis as Supplementary Figure 5. While some microchromosomes do not exbibit a pronounced decrease in mappability, we nevertheless observed an overall reduction for the ten smallest chromosomes compared to the rest of the karyotype.

We have revised the text to clarity the underlying causes of this reduced mappability and added the supplementary analysis to support our claim, see Line 255:

“However, Hi-C proved uninformative for microchromosomes: their short length and high repeat and GC content result in reduced mappability (Supplementary Figure 5), which in turn limits the reliable detection of meaningful contact patterns, a difficulty also encountered in other avian assemblies.”

Line 244: Or you could just generate enough ONT reads and skip HiFi altogether. See the paper linked above. While long is maybe good, you could just create a "normal" library with a high N50 read length (40 kb+), and 50-60x coverage and get almost T2T from only ONT.

[R] We acknowledge the strong potential of ONT sequencing for resolving genome assemblies. However, our recommendation to use multiple HiFi libraries was motived by the practical difficulty of obtaining sufficient amounts of high-molecular-weight DNA required for long-read ONT sequencing. In our experience, generating an ONT library with an N50 of ~38 kbp required 40 µg of input DNA, which may not be feasible for all samples or laboratories, both in terms of time and cost.

Line 246: I guess you go into this in more detail in the methods, but as it is, I don't understand how and what you are filtering. Are you removing sequences from the assembly? Which you don't know for certain are not biological part of the genome?

[R] We indeed filter sequences from the alignment. As detailed in the Materials and Methods section, the filtered regions correspond to specific repetitive sequences. While these regions are biologically part of the genome, as supported by telomeric and centromeric FISH signals, we considered them difficult to assemble reliably without dedicated approaches. Therefore, they were excluded to ensure robustness. We have clarified this point in the manuscript, see Line 266.

“Filtering of sequences containing high density of internal repeats, such as pericentromeric or centromeric reads, is also essential to prevent assembly artefacts. Although these regions are genuine components of the genome, as shown by FISH signals (Figure 2B), they are difficult to assemble reliably without dedicated approaches and can therefore introduce biases into the reconstruction of the rest of the genome.”

Line 250: I don't think this list is complete. You seem to be lacking https://www.biorxiv.org/content/10.1101/2025.10.14.682431v1 at least, and the paper linked above. Maybe many others also.

[R] We thank the reviewer for this comment. We acknowledge that additional relevant works may exist. While this list is not intended to be exhaustive, we have added the two referenced papers.

Lines 281-282: I am skeptical about your claim "more broadly, for the order Strigiformes" as mentioned a couple of times above. I guess it also depends on the definitions of "complete" and "accurate".

[R] We agree that the level of completeness of an assembly is subject to interpretation. For this reason, we revised the text, accordingly, see Line 308:

“Together, our phased, chromosome level assemblies provide a high-quality genomic resources genomic resource for the barn owl and contribute to the growing set of genomic resources available for the order Strigiformes”

Line 323: Which chemistry was used? R10 yields superior results compared to R9 usually.

[R] Indeed, R10 was used for the ONT sequencing. We specified it in the manuscript, see Line 359:

“Sequencing was performed on PromethION flow cells R10 (FLO-PRO114M)”

Line 347: What is the definition of "extreme repeats"? I have not seen this approach earlier, and wonder about the benefits of doing it. Did this mean that you did not try to assemble the centromere?

[R] This comment relates to their previous points on Line 323. In this context, “extreme repeats” refers to reads composed almost entirely of highly repetitive sequences: this definition has now been clarified in the revised manuscript, see Line 414:

“To reduce assembly artefacts from extreme repeats, i.e., reads composed almost entirely of highly repetitive sequences, we first counted 64 mers in HiFi reads with Jellyfish v2.3.0”

Due to their repetitive nature, these reads can produce misleading but apparently valid pairwise alignments. For example, truncated versions of the same centromeric read (missing sequence at the 5’ and/or 3’ ends) can align with high similarity, as illustrated in the attached incorrectAlignment.txt example, where two 4 kbp truncations of a 19 kbp read result in an apparent 10 kbp perfect match. More generally, determining whether distinct reads should be assembled into a contig is highly unreliable in such regions (see the attached incompatibleReads.txt example), as it would require exhaustive comparisons and multiple sequence alignments beyond the capabilities of standard assemblers relying on relatively short k-mers.

Given the high risk of generating incorrect assemblies, we excluded these reads from the assembly process and did not attempt to automatically assemble centromeric regions. Our observations indicate that barn owl centromeric repeats require specialized approaches, which will be addressed in a dedicated future study.

Line 382: Multiple assemblers, such as Verkko and hifiasm, do this internally. It is not clear to me why you do this manually.

[R] We did attempt to use the trio-based capabilities of hifiasm. However, this approach resulted in imbalances between haplomes, and BUSCO analyses showed more than 10% duplicated gene content. We therefore implemented the manual strategy described in the manuscript, which yielded more balanced haplotypes and substantially reduced duplication levels according to BUSCO.

Line 413: There is a lot of information in reads shorter than 25k.

[R] While shorter ONT reads can be informative, their lower accuracy compared to HiFi reads led us to apply a length threshold. Given that our HiFi reads already reached an average length of ~ 25 kbp (Supplementary Table 1), we aimed to avoid incorporating ONT reads of similar length but lower quality. Instead, we retained only longer ONT reads, which provide additional long-range information that compensate for their higher error rate.

Line 440: Maybe I misunderstand something here. You orient the chromosomes, so they start with p arm? (As recommended by EBP: https://www.earthbiogenome.org/report-on-assembly-standards [point l]). If you then add the centromeres (which can be of varying size), you will change all coordinates downstream, which means most of the chromosome since the q arm is longer. Is that correct? Since the centromere is not a point, you will most likely change the coordinates anyhow. I don't understand why you removed centromeric reads…

[R] In barn owl, all chromosomes except the Z chromosome are acrocentric. Using the convention where T denotes a telomere, t its reverse complement, and @ the centromere, a full chromosome sequenced can theoretically be represented as either:

(a) ttt---------------@-TTT or (b) ttt-@---------------TTT

Because we excluded centromeric reads (@), which are highly repetitive and cannot be reliably assembled

(see answers to previous comments), we obtained truncated representations:

(a’) ttt--------------- or (b’) ---------------TTT

We chose to orient all chromosomes according to (a’), i.e. starting from the telomeric end of the p arm. This orientation allows centromeric and downstream sequences to be added in the future without altering the existing coordinate system.

Line 446: How many contigs did you end up with? In table 1 it looks like you have just 46 contigs, but that's the number of chromosomes. You list the number of Ns in these assemblies also, so the amount of contigs is most likely many more than 46. You could mention how large your gaps are.

[R] The number of contigs is now reported in the “Prior to scaffolding [contigs]” column, for each haplome. Regarding the gaps, they have been inserted during the scaffolding (see Line 543 and 563 of M&Ms), from contigs to chromosomes, and their total size is reported in Table 1 for each haplome.

Line 501: You are replicating behaviour implemented in Merqury (https://github.com/marbl/merqury/wiki/3.-Phasing-assessment-with-hap-mers), which seems a bit strange to me. Meryl was published in the Merqury paper, so you could have seen this.

[R] We agree that Meryl is part of the Merqury toolkit. Our objective, however, was to address a very specific and transparent question: to quantify the extent to which k-mers from one parent are misassigned to the other haplome. To this end, we chose to implement a simple and explicit approach (14 lines of code, available in the manuscript GitHub repository under 4_PhasingEvaluation).

This choice allowed us to fully control and document each step of the procedure, rather than relying on an additional tool whose internal behaviour may not be immediately transparent without in-depth inspection. Overall, we acknowledge that similar functionality is implemented in Merqury but opted for this lightweight implementation to ensure clarity and reproducibility tailored to our specific question.

Reviewer #2: In this manuscript, the authors presented a chromosome-level, highly contiguous haplotype-resolved genome assembly of the barn owl. The manuscript is very well written and very detailed. I was, however, a bit confused by the duplicated manuscript in the file provided to me for review. My comments are based on the first version (pages 1-39 in the PDF).

[R] We thank the reviewer for their positive feedback on our manuscript and constructive comments. Please find below a detailed answer for each of the comments and suggestions you raised.

General comments:

Throughout the manuscript "-" are not shown but instead.

[R] We carefully checked the manuscript and did not identify any missing hyphens (“-”). This issue may have arisen from formatting during the bioRxiv submission or file conversion prior to transfer to GigaScience, which could also explain the presence of different versions too.

Abstract: L42: Calling microchromosomes "elements" seems very unspecific. I would maybe call them "DNA molecules"

[R] We agree and modified the abstract, opting for “DNA molecules” when referring to microchromosomes, as suggested by the reviewer, see Line 42 of the Tracked Changes version of the manuscript:

“However, avian genomes remain challenging due to numerous microchromosomes, small, typically < 20Mb, DNA molecules that are gene-, GC-, and repeat-rich.”

Introduction:

L96: "70 "pseudo-haplotypes”- pseudo haploid scaffolds or what does this exactly refer to?

[R] We have revised the manuscript to define more clearly what is meant by “pseudo-haplotypes” in this context and added a precise explanation, see Line 99:

“The current assembly generated using PacBio long reads and Bionano optical mapping consists of 70 “pseudo-haplotypes”, i.e., scaffolds representing a mosaic combination of the two haplotypes in which phase information is not preserved (N50 = 36.03 Mb)”

Results:

L130: Why was a homogametic male chosen and not a heterogametic female? I know sometimes this is the only sample available but the authors went a great length to produce a very high-quality genome assembly but it is incomplete with the W chromosome missing.

[R] The assembly was generated using material from the same individual as in Machado et al. (2021), namely a male, for which sequencing data were already available. We also chose not to tackle the W chromosome, as its highly repetitive structure makes it particularly challenging to assemble with comparable contiguity and completeness as the rest of the karyotype. Achieving this would have required substantial additional effort and could be addressed in a dedicated future study.

L161: It is not clear to the reader here where this W chromosome now comes from and what FISH signals are. As this is the first mention of it, please have a short explanation of what FISH means in this context.

[R] We have revised the manuscript accordingly by clarifying the origin of the W chromosome data and by providing a brief description of the FISH approach, see Line 173:

“To investigate the proportion and types of repeats on the W chromosome, which is absent from the focal male assembly, we performed Fluorescence In Situ Hybridisation (FISH) on a female individual (Figure 2B). This technique uses fluorescent probes to localise specific DNA sequences on chromosomes, here targeting telomeric and centromeric repeats (see Material and Method section for details). The W chromosome exhibits strong signals for both probe types, suggesting a higher proportion of repeats in this chromosome compared to the rest of the barn owl genome. ”

Figures and Tables:

Figure 1: Very nice design but the component and especially the text are too small to read without zooming in. Also, in my version the resolution is poor, making it even more difficult to read and look for details in the contact maps.

[R] We acknowledge that the resolution of the figures in the submitted PDF was suboptimal. The initial submission was made via bioRxiv and subsequently transferred to GigaScience, without the possibility to include additional high-resolution figure files. We expect this issue to be resolved in the resubmitted version. In the meantime, we have reworked the figures to further improve their clarity. The updated figures are provided as attachments.

Figure 2: Also, too small and low resolution in the review copy. Please make sure the text is readable, and the figures are large enough and high-resolution.

[R] See the previous answer for details.

Figure 3: Text is also too small I cannot read the axis labels in my A4 printed version, especially in D and E.

[R] See the previous answers for details.

Table 1: Tables should have a title on top, as it is a scientific standard, not a caption below.

[R] We added a title to Table 1 accordingly.

Material and Methods:

L295: processed

[R] Fixed.

L508 and following: I am missing more detailed QC steps. Like blobplots to identify potential contamination, Merqury QV and completeness values etc.

[R] We thank the reviewer for this comment. As described in the dedicated M&Ms section, we already assessed genome completeness using BUSCO and evaluated its contiguity with QUAST, with results reported in Table 1. For clarity, we have revised and expanded the corresponding M&Ms section. In addition, we had already assessed the phasing accuracy of the two haplotypes, as described in a dedicated M&M section.

Following the reviewer's recommendation, we have now added an explicit assessment of potential contamination. We performed blobplot analyses at the contig level (i.e., prior to scaffolding) for both the maternal and paternal haplome. The methodological details have been added to the Genome quality control section of the M&Ms, see Line 618.

"Genome contamination was also evaluated at the contig level for the parental assemblies using BlobToolKit v4.5.3 [55]. HiFi reads were aligned to the maternal and paternal assembly FASTA sequences using minimap2 (v2.24-r1122) to generate BAM files for coverage estimation. Taxonomic assignments were obtained by aligning assembly FASTA sequences against the NCBI nucleotide (nt) database (July 2021 release) using BLASTN (BLAST+ v2.14.0; parameters: -outfmt '6 qseqid staxids bitscore std' -max_target_seqs 10 -max_hsps 1 -evalue 1e-25). Blob plots were generated using species-level taxonomic assignments (bestsumorder_species) and sequence coverage information. The resulting plots were visually inspected for contigs with taxonomic assignments inconsistent with birds, which could indicate potential contamination."

Briefly, most BLAST hits were assigned to Tyto alba, while a minor portion (tiny contigs) corresponded to other avian species in both haplomes. We did not interpret these minor assignments as evidence of significant contamination, but rather as expected due to phylogenetic proximity. These results are now reported in the revised manuscript, see Line 152:

"To assess the amount of potential contamination, we ran a blobplot analyses at the contig level (i.e., prior to scaffolding) for both the maternal and paternal haplome (Supplementary Figure 3 and 4). Both did not exhibit strong evidence of contamination: most BLAST hits were assigned to Tyto alba, while a minor portion (tiniest contigs) corresponded to other avian species in both haplomes, as expected due to phylogenetic proximity."

L511: Why was Busco 6 used but the old aves_odb10 not odb12?

[R] We agree with the reviewer's comment, and we re-ran the BUSCO analysis using the aves_odb12 dataset instead of aves_odb10. We've updated the Results and Materials and Methods sections accordingly. Notably, the overall completeness metrics remained highly consistent with our previous results, and no substantial differences were observed.

L572 and 585: subheaders are difficult to identify as they are. Typesetting with likely makes them more visible, but I would at least add an empty line between sections for the next submission.

[R] We added empty lines between every section throughout the entire manuscript to improve readability.

Cookie Preference Center

We use cookies which are necessary to make our site work. We may also use additional cookies to analyze, improve and personalize our content and your digital experience. For more information, see our Cookie Policy.

You may choose not to allow some types of cookies. However, blocking some types may impact your experience of our site and the services we are able to offer. See the different category headings below to find out more or change your settings.

Allow all

Manage Consent Preferences

Strictly Necessary Cookies

Always active

These cookies are necessary for the website to function and cannot be switched off in our systems. They are usually only set in response to actions made by you which amount to a request for services, such as setting your privacy preferences, logging in or filling in forms. You can set your browser to block or alert you about these cookies, but some parts of the site will not then work.

Functional Cookies

These cookies enable the website to provide enhanced functionality and personalisation. They may be set by us or by third party providers whose services we have added to our pages. If you do not allow these cookies then some or all of these services may not function properly.

Performance Cookies

These cookies allow us to count visits and traffic sources so we can measure and improve the performance of our site. They help us to know which pages are the most and least popular and see how visitors move around the site.

Targeting Cookies

These cookies may be set through our site by our advertising partners. They may be used by those companies to build a profile of your interests and show you relevant adverts on other sites. If you do not allow these cookies, you will experience less targeted advertising.

Cookie List

Clear

Apply Cancel

Consent Leg.Interest

Confirm my choices

Powe
