# Supplementary material for: A chromosome-level, haplotype-resolved genome assembly for the barn owl, Tyto alba
Source: Gigascience. 2026 Sep 8;15:giag092. doi: 10.1093/gigascience/giag092 (PMC13615541; doi:10.1093/gigascience/giag092)
Supplement: Supplementary file 3 [file giag092_giga-d-26-00095_revision_1.pdf]

GigaScience

A chromosome-level, haplotype-resolved genome assembly for the barn owl, Tyto alba

--Manuscript Draft--

**Manuscript Number:**	GIGA-D-26-00095R1
**Full Title:**	A chromosome-level, haplotype-resolved genome assembly for the barn owl, Tyto alba
**Article Type:**	Research
**Funding Information:**	Swiss National Science Foundation (310030_215709) — Not applicable
**Abstract:**	Recent advances in long-read sequencing have enabled near telomere-to-telomere (T2T) assemblies across diverse taxa. However, avian genomes remain challenging due to numerous microchromosomes, small, typically < 20Mb, DNA molecules that are gene-, GC-, and repeat-rich. As a consequence, microchromosomes are often missing from genome assemblies. Here, we present a chromosome-level, haplotype-resolved genome assembly for the Western barn owl (Tyto alba). Using a trio-binning strategy with Illumina parental reads combined with PacBio HiFi and Oxford Nanopore Technologies data, we generated two phased contig sets. These were scaffolded into 40 linkage groups using a linkage map. Comparative analyses identified unplaced HiFi scaffolds corresponding to microchromosomes, which we integrated into six additional microchromosomes using long reads information. The two assemblies present 46 chromosomes, matching the karyotype of the species. They exhibit strong synteny between parental haplotypes, except for a ~38 Mb complex region on chromosome 7 containing nested inversions. This high-quality reference provides a haplotype-resolved and chromosome-level genome for Strigiformes, enabling fine-scale studies of structural variation and avian genome evolution.
**Corresponding Author:**	Tristan Cumer<br>University of Lausanne<br>Lausanne, SWITZERLAND
**Corresponding Author Secondary Information:**	
**Corresponding Author's Institution:**	University of Lausanne
**Corresponding Author's Secondary Institution:**	
**First Author:**	Hugo Corval
**First Author Secondary Information:**	
**Order of Authors:**	Hugo Corval
	Anne-Lyse Ducrest
	Marianne Bachmann Salvy
	Allison Burns
	Alexandros Topaloudis
	Celine Simon
	Elisa Cora
	Daniel Cavaleri
	Bettina Almasi
	Alexandre Roulin
	Christian Iseli
	Nicolas Guex
	Tristan Cumer
	Jerome Goudet

Powered by Editorial Manager® and ProduXion Manager® from Aries Systems Corporation

Order of Authors Secondary Information:	
Response to Reviewers:	Reviewer #1: General comments<br>It is always good that high quality genome assemblies are created and made available to the community. However, I have some comments below.<br><br>[R] We thank the reviewer for this positive feedback and constructive comments on the manuscript. Please find below a detailed answer for each of the comments and suggestions you raised.<br><br>The authors have used a complicated assembly without justifying why this was done. One of the consequences of this was that the reads coming from the centromeres were excluded from the assembly, in addition to other reads. Instead of assembling the (HiFi) reads with hifiasm (or Verkko) and supplying k-mers from parents as is usually done for trio-assemblies, the authors separated reads before assembly. Both hifiasm versions used by the authors have had the ability to utilize HiFi and ONT reads at the same time (https://github.com/chhylp123/hifiasm?tab=readme-ov-file#ultra-long-ont-integration, the last example is directly applicable here). Why that approach, which has been used multiple times in the literature, was not chosen, and a custom approach was used instead is not clear to me. The authors should at least have demonstrated that the custom approach was superior to the "standard" approach, thereby justifying why it was done.<br><br>[R] We thank the reviewer for raising this important point.<br>We agree that hifiasm natively supports the joint integration of HiFi reads, ONT ultra-long reads, and parental k-mer information, and that this “standard” approach has been successfully used in multiple studies.<br>In our study, we initially followed this standard strategy by running hifiasm in trio mode with all HiFi and ONT reads simultaneously, using parental k-mer databases as recommended. However, this approach consistently led to highly unbalanced phased assemblies, with a large discrepancy in haplotype sizes (1.76 Gb for the paternal haplotype versus 1.21 Gb for the maternal haplotype), which deviates substantially from the expected genome size (~1.3 Gb) and from each other. In addition, we observed inconsistencies in assembly quality metrics (e.g., BUSCO completeness and duplication rates) and suboptimal phasing performance.<br>We first investigated whether this imbalance could be attributed to incorrect estimation of homozygous coverage, a known issue in hifiasm that can be mitigated using the --hom-cov parameter. However, the k-mer spectrum of our dataset showed a well-defined heterozygous peak (~58×) and homozygous peak (~119×) (see attached log file), consistent with expectations and indicating that coverage inference was accurate. Inspection of the k-mer spectrum and read content revealed a substantial fraction of extremely repetitive reads, including centromeric and pericentromeric sequences spanning entire long reads (>20 kb). Such reads are known to complicate overlap-based assembly by generating large numbers of ambiguous overlaps and by disproportionately influencing graph construction. Based on these observations, we hypothesised that these sequences were biasing the assembly process and contributing to haplotype imbalance.<br>To address this issue, we implemented a targeted read classification and filtering strategy, in which HiFi reads were stratified according to the presence of highly repeated motifs. Reads dominated by centromeric-like and kinetochore-like repeats were excluded, while other categories with repeated motifs present only in a fraction of the reads (e.g., telomeric and microsatellite-containing reads), less likely to confuse the assembler, were retained. Re-running the assembly with this filtered dataset resulted in improved GC consistency between haplotypes and a reduction in haplotype size discrepancy, although some imbalance persisted.<br><br>We then considered the limitations of k-mer–based read partitioning in the presence of moderate repeats, low-divergence allelic regions, and unequal parental sequencing depth (~38× for the male vs ~21× for the female). Even after removing the most extreme repetitive reads, these factors may still affect phasing and assembly performance, leading to the persistent haplotype imbalance.<br><br>To overcome these limitations, we developed a custom allele-sorting strategy prior to assembly, based on identification of parent-specific k-mers across multiple subsampled parental datasets to mitigate the large parental sequencing depth

Powered by Editorial Manager® and ProduXion Manager® from Aries Systems Corporation

imbalance. This approach allowed us to explicitly partition reads by parental origin before assembly and to perform independent haplotype-specific assemblies, thereby reducing ambiguity in read assignment and improving phasing accuracy. Importantly, this strategy was not used as a replacement for standard hifiasm functionality, but rather as a data-driven refinement motivated by observed assembly artefacts that could not be resolved using existing parameters alone. The full rationale and intermediate results supporting this approach have now been clarified in the revised Materials and Methods section, see line 412 to 417 of the Tracked Changes version of the manuscript.

Finally, we acknowledge that the exclusion of centromeric-like reads results in the absence of complete centromeric sequences in the present assembly. This was a deliberate trade-off to improve overall assembly contiguity and haplotype balance. We are currently developing a complementary analysis specifically aimed at resolving chromosome-level centromeric sequences and architecture, which will be presented in a dedicated future study.

The manuscript does not report contig statistics. As far as I can see, all references to N50 are to scaffolds, and not contigs. The only ones referring to N50 contigs are to an old assembly. Knowing the N50 contig at different stages in the complicated assembly process would help us to understand how the process worked and how much it (potentially) improved the assemblies.

[R] The values reported in the “Hifiasm” columns of Table 1 corresponded to the non-scaffolded contigs immediately with hifiasm. The columns named “Final” corresponded to the number of sequences obtained at the end of the scaffolding procedure. To avoid confusion and improve clarity, the mentioned columns have been renamed “Prior to scaffolding [contigs]”, and “After scaffolding [chromosomes]” respectively.

Several places in the manuscript it is stated that this is the "first haplotype-resolved and chromosome-level genome for Strigiformes". This is not justified well enough, and I point to several other assemblies that might fit this definition.

[R] We agree with the reviewer that the claim of a “first haplotype-resolved and chromosome-level genome for Strigiformes” may be open to interpretation depending on how these criteria are defined. While other assemblies are phased and include scaffolding to the chromosome-level for some chromosomes, in the assemblies we examined, not all chromosomes are consistently resolved. To our knowledge, the assembly presented in this manuscript is the first to also include all micro-chromosomes at this level of completeness. However, we acknowledge that such claims depend on a comprehensive evaluation of all existing assemblies and on the specific definition applied. To avoid potential ambiguity and overstatement, we have therefore revised the manuscript to moderate this claim accordingly.

See Lines 53, 231, 236 and 308

Specific comments:

Line 53: I find it a bit strange that you state that this is "first haplotype-resolved and chromosome-level genome for Strigiformes". Searching at GoaT (https://goat.genomehubs.org/) lists 5 Strigiformes with chromosome level assemblies (Athene noctua, Bubo scandiacus, Strix aluco, Strix nebulosa and Strix uralensis), where Athene noctua, Bubo scandiacus, and Strix aluco are all haplotype resolved. You even refer to the publication of one of these (Baalsrud HT et al 2025). Strix aluco has been on NCBI since September 2023, Athene noctua since February 2025 and Bubo scandiacus since March 2025. I guess you imply that these assemblies are only pseudo-haplotypes, but it is not clear to me.

[R] As previously mentioned, we agree that the claim of a “first haplotype-resolved and chromosome-level genome for Strigiformes” can be subject to interpretation depending on the criteria applied. We thank the reviewer for highlighting these additional assemblies. However, while these genomes are indeed phased and scaffolded to the chromosome level, they do not appear to include all the chromosome. In particular, they retain a substantial number of unplaced scaffolds (e.g., 675 for Athene noctua,

Powered by Editorial Manager® and ProduXion Manager® from Aries Systems Corporation

1,070 for Bubo scandiacus or 161 for Strix aluco), which likely correspond in part to unresolved micro-chromosome sequences.

Nevertheless, we acknowledge that such comparisons depend on methodological differences and definitions of completeness. To avoid ambiguity and potential overstatement, we have therefore removed the claim of being the “first” from the manuscript and revised the text accordingly.

Line 86: It is important to note that the sequence in the micro-chromosomes might be in the assembly, but it is difficult or impossible to distinguish from so-called shrapnel (unplaced sequence, often repeats).

[R] Indeed, micro-chromosomes sequences are often contained in so-called shrapnel, left as unplaced scaffolds in the assembly. We revised the text to add this nuance, see Line 84:

“Because of these properties, many avian genome assemblies contain fewer chromosomes than the actual karyotype, with microchromosomes frequently missing or not fully resolved and left as unplaced scaffolds”

Line 89: I don't understand your comment here "...adoption of these approaches has been uneven across birds…" Firstly, getting the correct data require proper handling of samples (flash-frozen ideally, etc), and successful DNA isolation before sequencing. These steps might be expensive, and not all labs/projects might afford this. You write this as it is a conscious choice to not use ONT data, and I think it is mostly cost and other restrictions (maybe PacBio might be the only choice they have).

[R] We agree that the adoption of these approaches is not a matter of deliberate choice but rather reflects practical constraints. We have revised the text to better reflect these considerations and avoid implying that the uneven adoption is intentional, see Line 89:

“However, the adoption of these approaches across avian genomics has been constrained by practical challenges, including difficulties in obtaining high-quality samples, extracting enough ultra-high molecular DNA and the cost of sequencing. As a result, achieving full and accurate resolution of avian microchromosomes remains a major challenge.”

Figure 1: The resolution of this was a bit low, so hard to read the numbers.

[R] We acknowledge that the resolution of the figures in the submitted PDF was suboptimal. The initial submission was made via bioRxiv and subsequently transferred to GigaScience, without the possibility to include additional high-resolution figure files. We expect this issue to be resolved in the resubmitted version. In the meantime, we have reworked the figures to further improve their clarity. The updated figures are provided as attachments.

Table 1: The N50 and N90 here, what do these refer to? It looks like scaffold metrics, but good to be explicit especially when "# contigs" is right above. How many contigs do you have in the final assemblies? It is written as 46 here, but you also have gaps, so I guess the number is higher. You have 46 chromosomes I guess. You cannot state "# contigs" and report scaffolds/chromosomes. Have a separate line for that or something (or just state it somewhere).

[R] Indeed, N50 and N90 are classical assembly metrics that represent the minimum length of sequences that cover a cumulative 50% or 90% of the entire assembled genome. We clarified these definitions in Table 1. As mentioned in a previous answer, we also revised the table structure. In addition, we replaced the term “# contigs” with “# sequences” to encompass contigs, scaffolds and chromosomes.

Figure 2: This figure is difficult to interpret. Colours are unclear, symbols are unclear (difficult to distinguish Similar and Opposite orientation). Text is diffuse. The green and red circles are really hard to see.

[R] See previous answers to comments on Figure 1 for details. In the meantime, Figure

2 has also been re-worked for clarity and interpretability. More specifically, we changed the colour palette to distinguish between the two haplomes, increased the size of the chromosomes and corresponding telomeres and pericentromeres glyphs. We also modified the legend to make it easier to understand the different elements of the A panel.

Figure 3: Again, this is hard to see. You need higher resolution. I don't know if this is the fault of the authors or the journal.

[R] See previous answers to comments on Figure 1 for details. In the meantime, Figure 3 has also been re-worked for clarity and readability.

Figure 4: This is really nice. Where are the gaps in these two chromosomes? Does each "arrow" represent a contig?

[R] As indicated in the figure legend, Figure 4 shows an extract of the whole-genome alignment between the paternal and maternal haplotypes, focusing on chromosome 7. Only alignment blocks that are unique to this chromosome in both haplotypes are displayed. The apparent gaps, such as those at the right end of the maternal chromosome 7, correspond to highly repetitive regions (as reflected in the repeat profile below) that align to other parts of the genome and are therefore not shown here. Each arrow represents an alignment block generated by minimap2. All these clarifications have been added to Figure 4 legend.

Line 207: I understand what you want to say, but the T2T era is a consequence of better/longer reads, and not something that in itself has changed genome assembly.

[R] We agree that the T2T era is a consequence rather than a cause. We have revised the text, accordingly, see Line 225:

“Recent advances in long-read sequencing technologies have made it increasingly possible to reconstruct near-complete or telomere-to-telomere chromosomes across an increasing range of species.”

Line 209: "among the most challenging" just depends on what you compare to, and what kind of tools you have available. In my experience, codfishes are awful to assemble with PacBio HiFi, but works excellent with ONT. Likewise here, HiFi leads to incomplete assemblies for birds, but ONT have much better results. For other species/groups, ONT might work really poorly and HiFi much better.

[R] We agree that the difficulty of genome assembly is context-dependent and can vary substantially with the sequencing technology and assembly methods used, as well as across taxa. We have revised the text to avoid overgeneralisation, see Line 228:

“Yet birds remain challenging vertebrates to assemble owing to their numerous, small, GC-rich and repeat dense microchromosomes, which have historically been under represented or mis assembled in reference genomes”

Line 212: I have already protested against the use of "first haplotype resolved, chromosome level assemblies for an owl". You have to define this better, that is, define this assembly such that the species mentioned above is not part of this definition.

[R] We thank the reviewer for this comment and have revised the manuscript accordingly, see previous comments for detailed answer and line 53, 231, 236 and 308 for examples of in text modifications.

Line 234: What do you mean by poor mappability here? We have seen good signals for microchromosomes. See for instance Supplementary Figure 1 here: https://www.biorxiv.org/content/10.64898/2026.03.26.714473v1.full

[R] Indeed, in the case of Luscinia s. svecica, Hi-C signals performed well, even along microchromosomes. However, our statement refers to a more general context in which microchromosomes are known to be difficult to map due to their high repeat and GC content.

Powered by Editorial Manager® and ProduXion Manager® from Aries Systems Corporation

	To support this point, we now quantify mappability for each chromosome as the ratio of the cumulative length of mappable regions to the total chromosome length (see M&M for details, Line 678) and included this analysis as Supplementary Figure 5. While some microchromosomes do not exhibit a pronounced decrease in mappability, we nevertheless observed an overall reduction for the ten smallest chromosomes compared to the rest of the karyotype.<br><br>We have revised the text to clarity the underlying causes of this reduced mappability and added the supplementary analysis to support our claim, see Line 255:<br><br>"However, Hi-C proved uninformative for microchromosomes: their short length and high repeat and GC content result in reduced mappability (Supplementary Figure 5), which in turn limits the reliable detection of meaningful contact patterns, a difficulty also encountered in other avian assemblies."<br><br>Line 244: Or you could just generate enough ONT reads and skip HiFi altogether. See the paper linked above. While long is maybe good, you could just create a "normal" library with a high N50 read length (40 kb+), and 50-60x coverage and get almost T2T from only ONT.<br><br>[R] We acknowledge the strong potential of ONT sequencing for resolving genome assemblies. However, our recommendation to use multiple HiFi libraries was motived by the practical difficulty of obtaining sufficient amounts of high-molecular-weight DNA required for long-read ONT sequencing. In our experience, generating an ONT library with an N50 of ~38 kbp required 40 µg of input DNA, which may not be feasible for all samples or laboratories, both in terms of time and cost.<br><br>Line 246: I guess you go into this in more detail in the methods, but as it is, I don't understand how and what you are filtering. Are you removing sequences from the assembly? Which you don't know for certain are not biological part of the genome?<br><br>[R] We indeed filter sequences from the alignment. As detailed in the Materials and Methods section, the filtered regions correspond to specific repetitive sequences. While these regions are biologically part of the genome, as supported by telomeric and centromeric FISH signals, we considered them difficult to assemble reliably without dedicated approaches. Therefore, they were excluded to ensure robustness. We have clarified this point in the manuscript, see Line 266.<br><br>"Filtering of sequences containing high density of internal repeats, such as pericentromeric or centromeric reads, is also essential to prevent assembly artefacts. Although these regions are genuine components of the genome, as shown by FISH signals (Figure 2B), they are difficult to assemble reliably without dedicated approaches and can therefore introduce biases into the reconstruction of the rest of the genome."<br><br>Line 250: I don't think this list is complete. You seem to be lacking https://www.biorxiv.org/content/10.1101/2025.10.14.682431v1 at least, and the paper linked above. Maybe many others also.<br><br>[R] We thank the reviewer for this comment. We acknowledge that additional relevant works may exist. While this list is not intended to be exhaustive, we have added the two referenced papers.<br><br>Lines 281-282: I am skeptical about your claim "more broadly, for the order Strigiformes" as mentioned a couple of times above. I guess it also depends on the definitions of "complete" and "accurate".<br><br>[R] We agree that the level of completeness of an assembly is subject to interpretation. For this reason, we revised the text, accordingly, see Line 308:<br><br>"Together, our phased, chromosome level assemblies provide a high-quality genomic resources genomic resource for the barn owl and contribute to the growing set of genomic resources available for the order Strigiformes"

Powered by Editorial Manager® and ProduXion Manager® from Aries Systems Corporation

Line 323: Which chemistry was used? R10 yields superior results compared to R9 usually.

[R] Indeed, R10 was used for the ONT sequencing. We specified it in the manuscript, see Line 359:

“Sequencing was performed on PromethION flow cells R10 (FLO-PRO114M)”

Line 347: What is the definition of "extreme repeats"? I have not seen this approach earlier, and wonder about the benefits of doing it. Did this mean that you did not try to assemble the centromere?

[R] This comment relates to their previous points on Line 323. In this context, “extreme repeats” refers to reads composed almost entirely of highly repetitive sequences: this definition has now been clarified in the revised manuscript, see Line 414:

“To reduce assembly artefacts from extreme repeats, i.e., reads composed almost entirely of highly repetitive sequences, we first counted 64 mers in HiFi reads with Jellyfish v2.3.0”

Due to their repetitive nature, these reads can produce misleading but apparently valid pairwise alignments. For example, truncated versions of the same centromeric read (missing sequence at the 5’ and/or 3’ ends) can align with high similarity, as illustrated in the attached incorrectAlignment.txt example, where two 4 kbp truncations of a 19 kbp read result in an apparent 10 kbp perfect match. More generally, determining whether distinct reads should be assembled into a contig is highly unreliable in such regions (see the attached incompatibleReads.txt example), as it would require exhaustive comparisons and multiple sequence alignments beyond the capabilities of standard assemblers relying on relatively short k-mers.

Given the high risk of generating incorrect assemblies, we excluded these reads from the assembly process and did not attempt to automatically assemble centromeric regions. Our observations indicate that barn owl centromeric repeats require specialized approaches, which will be addressed in a dedicated future study.

Line 382: Multiple assemblers, such as Verkko and hifiasm, do this internally. It is not clear to me why you do this manually.

[R] We did attempt to use the trio-based capabilities of hifiasm. However, this approach resulted in imbalances between haplomes, and BUSCO analyses showed more than 10% duplicated gene content. We therefore implemented the manual strategy described in the manuscript, which yielded more balanced haplotypes and substantially reduced duplication levels according to BUSCO.

Line 413: There is a lot of information in reads shorter than 25k.

[R] While shorter ONT reads can be informative, their lower accuracy compared to HiFi reads led us to apply a length threshold. Given that our HiFi reads already reached an average length of ~ 25 kbp (Supplementary Table 1), we aimed to avoid incorporating ONT reads of similar length but lower quality. Instead, we retained only longer ONT reads, which provide additional long-range information that compensate for their higher error rate.

Line 440: Maybe I misunderstand something here. You orient the chromosomes, so they start with p arm? (As recommended by EBP: https://www.earthbiogenome.org/report-on-assembly-standards [point l]). If you then add the centromeres (which can be of varying size), you will change all coordinates downstream, which means most of the chromosome since the q arm is longer. Is that correct? Since the centromere is not a point, you will most likely change the coordinates anyhow. I don't understand why you removed centromeric reads…

[R] In barn owl, all chromosomes except the Z chromosome are acrocentric. Using the convention where T denotes a telomere, t its reverse complement, and @ the

centromere, a full chromosome sequenced can theoretically be represented as either:

(a) ttt---------------@-TTT or (b) ttt-@---------------TTT

Because we excluded centromeric reads (@), which are highly repetitive and cannot be reliably assembled (see answers to previous comments), we obtained truncated representations:

(a') ttt--------------- or (b') ---------------TTT

We chose to orient all chromosomes according to (a'), i.e. starting from the telomeric end of the p arm. This orientation allows centromeric and downstream sequences to be added in the future without altering the existing coordinate system.

Line 446: How many contigs did you end up with? In table 1 it looks like you have just 46 contigs, but that's the number of chromosomes. You list the number of Ns in these assemblies also, so the amount of contigs is most likely many more than 46. You could mention how large your gaps are.

[R] The number of contigs is now reported in the "Prior to scaffolding [contigs]" column, for each haplome. Regarding the gaps, they have been inserted during the scaffolding (see Line 543 and 563 of M&Ms), from contigs to chromosomes, and their total size is reported in Table 1 for each haplome.

Line 501: You are replicating behaviour implemented in Merqury (https://github.com/marbl/merqury/wiki/3.-Phasing-assessment-with-hap-mers), which seems a bit strange to me. Meryl was published in the Merqury paper, so you could have seen this.

[R] We agree that Meryl is part of the Merqury toolkit. Our objective, however, was to address a very specific and transparent question: to quantify the extent to which k-mers from one parent are misassigned to the other haplome. To this end, we chose to implement a simple and explicit approach (14 lines of code, available in the manuscript GitHub repository under 4_PhasingEvaluation).

This choice allowed us to fully control and document each step of the procedure, rather than relying on an additional tool whose internal behaviour may not be immediately transparent without in-depth inspection. Overall, we acknowledge that similar functionality is implemented in Merqury but opted for this lightweight implementation to ensure clarity and reproducibility tailored to our specific question.

Reviewer #2: In this manuscript, the authors presented a chromosome-level, highly contiguous haplotype-resolved genome assembly of the barn owl. The manuscript is very well written and very detailed. I was, however, a bit confused by the duplicated manuscript in the file provided to me for review. My comments are based on the first version (pages 1-39 in the PDF).

[R] We thank the reviewer for their positive feedback on our manuscript and constructive comments. Please find below a detailed answer for each of the comments and suggestions you raised.

General comments:

Throughout the manuscript "-" are not shown but instead.

[R] We carefully checked the manuscript and did not identify any missing hyphens ("-"). This issue may have arisen from formatting during the bioRxiv submission or file conversion prior to transfer to GigaScience, which could also explain the presence of different versions too.

Abstract: L42: Calling microchromosomes "elements" seems very unspecific. I would

maybe call them "DNA molecules"

[R] We agree and modified the abstract, opting for “DNA molecules” when referring to microchromosomes, as suggested by the reviewer, see Line 42 of the Tracked Changes version of the manuscript:

“However, avian genomes remain challenging due to numerous microchromosomes, small, typically < 20Mb, DNA molecules that are gene-, GC-, and repeat-rich.”

Introduction:

L96: "70 "pseudo-haplotypes”- pseudo haploid scaffolds or what does this exactly refer to?

[R] We have revised the manuscript to define more clearly what is meant by “pseudo-haplotypes” in this context and added a precise explanation, see Line 99:

“The current assembly generated using PacBio long reads and Bionano optical mapping consists of 70 “pseudo-haplotypes”, i.e., scaffolds representing a mosaic combination of the two haplotypes in which phase information is not preserved (N50 = 36.03 Mb)”

Results:

L130: Why was a homogametic male chosen and not a heterogametic female? I know sometimes this is the only sample available but the authors went a great length to produce a very high-quality genome assembly but it is incomplete with the W chromosome missing.

[R] The assembly was generated using material from the same individual as in Machado et al. (2021), namely a male, for which sequencing data were already available. We also chose not to tackle the W chromosome, as its highly repetitive structure makes it particularly challenging to assemble with comparable contiguity and completeness as the rest of the karyotype. Achieving this would have required substantial additional effort and could be addressed in a dedicated future study.

L161: It is not clear to the reader here where this W chromosome now comes from and what FISH signals are. As this is the first mention of it, please have a short explanation of what FISH means in this context.

[R] We have revised the manuscript accordingly by clarifying the origin of the W chromosome data and by providing a brief description of the FISH approach, see Line 173:

“To investigate the proportion and types of repeats on the W chromosome, which is absent from the focal male assembly, we performed Fluorescence In Situ Hybridisation (FISH) on a female individual (Figure 2B). This technique uses fluorescent probes to localise specific DNA sequences on chromosomes, here targeting telomeric and centromeric repeats (see Material and Method section for details). The W chromosome exhibits strong signals for both probe types, suggesting a higher proportion of repeats in this chromosome compared to the rest of the barn owl genome. ”

Figures and Tables:

Figure 1: Very nice design but the component and especially the text are too small to read without zooming in. Also, in my version the resolution is poor, making it even more difficult to read and look for details in the contact maps.

[R] We acknowledge that the resolution of the figures in the submitted PDF was suboptimal. The initial submission was made via bioRxiv and subsequently transferred to GigaScience, without the possibility to include additional high-resolution figure files. We expect this issue to be resolved in the resubmitted version. In the meantime, we have reworked the figures to further improve their clarity. The updated figures are provided as attachments.

Figure 2: Also, too small and low resolution in the review copy. Please make sure the text is readable, and the figures are large enough and high-resolution.

[R] See the previous answer for details.

Figure 3: Text is also too small I cannot read the axis labels in my A4 printed version, especially in D and E.

[R] See the previous answers for details.

Table 1: Tables should have a title on top, as it is a scientific standard, not a caption below.

[R] We added a title to Table 1 accordingly.

Material and Methods:

L295: processed

[R] Fixed.

L508 and following: I am missing more detailed QC steps. Like blobplots to identify potential contamination, Merqury QV and completeness values etc.

[R] We thank the reviewer for this comment. As described in the dedicated M&Ms section, we already assessed genome completeness using BUSCO and evaluated its contiguity with QUAST, with results reported in Table 1. For clarity, we have revised and expanded the corresponding M&Ms section. In addition, we had already assessed the phasing accuracy of the two haplotypes, as described in a dedicated M&M section.

Following the reviewer's recommendation, we have now added an explicit assessment of potential contamination. We performed blobplot analyses at the contig level (i.e., prior to scaffolding) for both the maternal and paternal haplome. The methodological details have been added to the Genome quality control section of the M&Ms, see Line 618.

"Genome contamination was also evaluated at the contig level for the parental assemblies using BlobToolKit v4.5.3 [55]. HiFi reads were aligned to the maternal and paternal assembly FASTA sequences using minimap2 (v2.24-r1122) to generate BAM files for coverage estimation. Taxonomic assignments were obtained by aligning assembly FASTA sequences against the NCBI nucleotide (nt) database (July 2021 release) using BLASTN (BLAST+ v2.14.0; parameters: -outfmt '6 qseqid staxids bitscore std' -max_target_seqs 10 -max_hsps 1 -evalue 1e-25). Blob plots were generated using species-level taxonomic assignments (bestsumorder_species) and sequence coverage information. The resulting plots were visually inspected for contigs with taxonomic assignments inconsistent with birds, which could indicate potential contamination."

Briefly, most BLAST hits were assigned to Tyto alba, while a minor portion (tiny contigs) corresponded to other avian species in both haplomes. We did not interpret these minor assignments as evidence of significant contamination, but rather as expected due to phylogenetic proximity. These results are now reported in the revised manuscript, see Line 152:

"To assess the amount of potential contamination, we ran a blobplot analyses at the contig level (i.e., prior to scaffolding) for both the maternal and paternal haplome (Supplementary Figure 3 and 4). Both did not exhibit strong evidence of contamination: most BLAST hits were assigned to Tyto alba, while a minor portion (tiniest contigs) corresponded to other avian species in both haplomes, as expected due to phylogenetic proximity."

L511: Why was Busco 6 used but the old aves_odb10 not odb12?

Powered by Editorial Manager® and ProduXion Manager® from Aries Systems Corporation

	[R] We agree with the reviewer's comment, and we re-ran the BUSCO analysis using the aves_odb12 dataset instead of aves_odb10. We've updated the Results and Materials and Methods sections accordingly. Notably, the overall completeness metrics remained highly consistent with our previous results, and no substantial differences were observed.<br><br>L572 and 585: subheaders are difficult to identify as they are. Typesetting with likely makes them more visible, but I would at least add an empty line between sections for the next submission.<br><br>[R] We added empty lines between every section throughout the entire manuscript to improve readability.
**Additional Information:**	
**Question**	**Response**
Are you submitting this manuscript to a special series or article collection?	No
**Experimental design and statistics**<br><br>Full details of the experimental design and statistical methods used should be given in the Methods section, as detailed in our Minimum Standards Reporting Checklist. Information essential to interpreting the data presented should be made available in the figure legends.<br><br>Have you included all the information requested in your manuscript?	Yes
**Resources**<br><br>A description of all resources used, including antibodies, cell lines, animals and software tools, with enough information to allow them to be uniquely identified, should be included in the Methods section. Authors are strongly encouraged to cite Research Resource Identifiers (RRIDs) for antibodies, model organisms and tools, where possible.<br><br>Have you included the information requested as detailed in our Minimum Standards Reporting Checklist?	Yes
**Availability of data and materials**	Yes

All datasets and code on which the conclusions of the paper rely must be either included in your submission or deposited in publicly available repositories (where available and ethically appropriate), referencing such data using a unique identifier in the references and in the "Availability of Data and Materials" section of your manuscript.<br><br>Have you have met the above requirement as detailed in our Minimum Standards Reporting Checklist?	
GigaScience has policies and guidelines in place for the use of generative AI-writing tools such as ChatGPT. If you have used such writing tools to assist with writing the manuscript this must be declared and cited in the text. Authors should not list AI-writing tools and other AI-assisted technologies as an author or co-author and should acknowledge that they are fully responsible for text generated or refined by AI-writing tools.<p><br><br>A summary of use (particularly in the introduction or among methods) needs to be included at the end of the paper, and the outputs should also be included as a supplementary file hosted in GigaDB or other open repositories. Please read our guidelines for more information. <p><br><br>By submitting to GigaScience, you are aware of the journal's AI-writing tools policy, and if you have declared use of such tools below, you have acknowledged this where appropriate in your manuscript and have made a summary of use and outputs available. <p> <b>AI-assisted writing tools have been used in the preparation of this manuscript?	No

Manuscript

Click here to access/download;Manuscript;NewRefGenome_GigaScience_v

A chromosome-level, haplotype-resolved genome assembly for the barn owl, *Tyto alba*

Hugo Corval[1,2*], Anne-Lyse Ducrest[1], Marianne Bachmann Salvy[1,2], Allison Burns[3,4], Alexandros Topaloudis[1,2], Céline Simon[1], Elisa Cora[5], Daniel Cavaleri[5], Bettina Almasi[6], Alexandre Roulin[1], Christian Iseli[3,4], Nicolas Guex[3,4+], Tristan Cumer[1,2*], Jérôme Goudet[1,2+]

* corresponding authors. Contact details: Department of Ecology and Evolution, Biophore Building, University of Lausanne, CH-1015 Lausanne, Switzerland. Email: hugo.corval@unil.ch; tristan.cumer@unil.ch

+ co-senior authors. Contact details: nicolas.guex@unil.ch; jerome.goudet@unil.ch

1 - Department of Ecology and Evolution, University of Lausanne, Lausanne, Switzerland

2 - Swiss Institute of Bioinformatics, Lausanne, Switzerland

3 - Bioinformatics Competence Center (BICC), École Polytechnique Fédérale de Lausanne, Lausanne, Switzerland.

4 - Bioinformatics Competence Center (BICC), University of Lausanne, Lausanne, Switzerland.

5 - Gene Expression Core Facility (GECF), School of Life Sciences, École Polytechnique Fédérale de Lausanne, Lausanne, Switzerland

6 - Swiss Ornithological Institute, Seerose 1, Sempach, Switzerland

Orcid

Hugo Corval - 0009-0008-1842-7754

Anne-Lyse Ducrest - 0000-0001-6412-2769

Marianne Bachmann Salvy - 0009-0006-1528-8168

Allison Burns - 0000-0001-7153-3160

Alexandros Topaloudis - 0000-0002-0909-8695

Céline Simon

Elisa Cora - 0000-0002-5065-362X

Daniel Cavaleri - 0009-0009-2113-0510

Bettina Almassi - 0000-0003-4962-8117

Alexandre Roulin - 0000-0003-1940-6927

Christian Iseli - 0000-0002-2296-2863

Nicolas Guex - 0000-0001-6023-0519

Tristan Cumer - 0000-0002-0276-7462

Jérôme Goudet - 0000-0002-5318-7601

Abstract

Recent advances in long-read sequencing have enabled near telomere-to-telomere (T2T) assemblies across diverse taxa. However, avian genomes remain challenging due to numerous microchromosomes, small, typically < 20Mb, DNA molecules that are gene-, GC-, and repeat-rich. As a consequence, microchromosomes are often missing from genome assemblies. Here, we present a chromosome-level, haplotype-resolved genome assembly for the Western barn owl (*Tyto alba*). Using a trio-binning strategy with Illumina parental reads combined with PacBio HiFi and Oxford Nanopore Technologies data, we generated two phased contig sets. These were scaffolded into 40 linkage groups using a linkage map. Comparative analyses identified unplaced HiFi scaffolds corresponding to microchromosomes, which we integrated into six additional microchromosomes using long reads information. The two assemblies present 46 chromosomes, matching the karyotype of the species. They exhibit strong synteny between parental haplotypes, except for a ~38 Mb complex region on chromosome 7 containing nested inversions. This high-quality reference provides a haplotype-resolved and chromosome-level genome for *Strigiformes*, enabling fine-scale studies of structural variation and avian genome evolution.

Keywords

Tyto alba, Strigiformes, chromosome-level, haplotype-resolved, genome assembly, microchromosomes, chromosomal inversion

Background

In recent years, genomics has entered the telomere-to-telomere (T2T) era. This shift began with the first complete human genome (T2T-CHM13) [1] and quickly expanded to other species, from crops [2,3] to livestock [4] and primates [5]. These milestones were achieved thanks to the advent of long-read sequencing technologies, mostly led by Pacific Biosciences (PacBio) and Oxford Nanopore Technologies (ONT), combined with chromosome conformation contact Hi-C sequencing [6]. With this resolution, assemblies now expose complex regions, such as the major histocompatibility complex (MHC) or the Killer-cell immunoglobulin receptors (KIRs) locus, that were previously poorly characterised due to extreme polymorphism and repetitive structure [7–11].

Although these recent advances have transformed genome assembly, significant challenges remain. One difficulty of genome assembly is reconstructing both parental haplotypes in diploid species rather than a mosaic consensus. Common strategies include trio binning with parental short reads [12,13], Hi-C phasing coupled to long, accurate reads [14,15], and Strand-seq for chromosome length phasing [16,17]. When successful, haplotype-resolved assemblies can improve the discovery of complex structural variants [12,18–20]. Still, phasing can be limited by repeat- and GC-rich regions that promote switches or mis-joins [21], by genomes with large size, high heterozygosity or polyploidy (common in plants) [22], and by practical constraints such as the need for parental samples (trio binning) and the cost/complexity of specialised libraries. Consequently, fully phased references are still less common than haploid assembly in many taxa [11,22].

The genome reconstruction also varies widely across taxa. In vertebrates, bird genomes are difficult to assemble due to the presence of numerous microchromosomes [23,24]. These microchromosomes share features that hamper their reconstruction: they are small, gene-rich, GC-rich and contain a high proportion of repetitive elements [24,25]. Because of these properties, many avian genome assemblies contain fewer chromosomes than the actual karyotype, with microchromosomes frequently missing or not fully resolved and left as

unplaced scaffolds [24]. Recently, the introduction of Oxford Nanopore ultra-long reads has improved their recovery, leading to more complete assemblies in species such as chicken [26], bustard [27], and mallard [25,28]. However, the adoption of these approaches across avian genomes has been constrained by practical challenges, including difficulties in obtaining high-quality samples, extracting enough ultra-high molecular DNA and the cost of sequencing. As a result, achieving full and accurate resolution of avian microchromosomes remains a major challenge.

Among owls (*Strigiformes)*, genome assembly poses extra difficulties. Their karyotypes feature many microchromosomes and rearrangements [29], making synteny with well-assembled model species less informative [30]. The Common barn owl (*Tyto alba* - NCBI:txid56313) illustrates this difficulty. The species has 46 pairs of chromosomes [31] and a complete genome assembly has yet to be achieved. The current assembly generated using PacBio long reads and Bionano optical mapping consists of 70 “pseudo-haplotypes”, i.e., scaffolds representing a mosaic combination of the two haplotypes in which phase information is not conserved (N50 = 36.03 Mb) [32]. This was a major improvement from the previous version, which contained 21,509 contigs with a N50 of 4.62 Mb [33]. Nevertheless, additional work is needed to reach a chromosome-level assembly that matches the 46 pairs observed in the barn owl karyotype.

In this study, we aim to produce a chromosome-level, haplotype-resolved reference genome for the barn owl (*Tyto alba*). We achieve accurate chromosome phasing through trio-binning using Illumina parental reads. We construct unitigs from HiFi PacBio and ONT data, which we then scaffold into chromosomes using linkage map information [34]. Finally, we leverage the gene-rich nature of microchromosomes to manually curate unplaced unitigs, based on the presence of genes typically found on microchromosomes in other species. With this integrated approach, we deliver a high-quality genomic resource, add a chromosome-level reference of an owl, and resolve previously uncharacterised complex structures.

Results

Chromosome-scale, haplotype resolved assemblies

Using PacBio HiFi reads (150.8 Gb, ~coverage of 116x for a genome of 1.3Gb, Supplementary Table 1), Oxford Nanopore reads (40.2 Gb, ~coverage of 31x, Supplementary Table 1), and linkage information, we generated two chromosome-level, haplotype-resolved assemblies of the barn owl (*Tyto alba*). Each haplotype comprised 46 chromosomes, matching the expected karyotype (Figure 1; Table 1; Supplementary Table 2). The paternal assembly spanned 1.27 Gbp (N50 = 36.7 Mb; N90 = 15.5 Mb, Figure 1A), and the maternal assembly spanned 1.29 Gbp (N50 = 38.3 Mb; N90 = 15.1 Mb, Figure 1B). Both haplomes showed a slight increase in size compared to previous assembly (GCF_018691265.1: 1.25 Gbp, Table 1). Hi-C contact maps showed strong, chromosome-scale diagonals with no spurious trans-chromosomal signals, consistent with high contiguity and correct scaffolding (Figure 1C-D).

We quantified phasing accuracy by leveraging k- mers unique to each parental read set. For each haplotype assembly, we computed the switch error rate as the proportion of parental- specific k- mers incorrectly assigned to the opposite haplotype relative to the total number of parental- specific k- mers detected across both assemblies. This analysis indicated that 7.8% of paternal- specific k- mers were misplaced into the maternal haplotype, while 7.5% of maternal- specific k- mers were incorrectly assigned to the paternal haplotype.

As the assembly was constructed from a homogametic male (ZZ), identification of the sexual chromosome could not be based on identification of an heterochromatide unique in each assembly. Instead, to identify the Z chromosome, we used a protein-based homology with other avian species. Species such as chicken (*Gallus gallus*) and zebra finch (*Taeniopygia guttata*) showed that proteins encoded on their Z chromosome mapped predominantly to our largest assembled chromosome (Supplementary Figure 1 and 2). This largest chromosome was also the only metacentric chromosome in the assembly, while all remaining chromosomes were acrocentric (Figure 2B).

Relative to prior references, gaps were greatly reduced: 110,000 Ns (0.01%) in the paternal and 600,000 Ns (0.05%) in the maternal haplotype, compared with 51,418,967 Ns (4.11%) in T.alba_DEE_v4.0 (GCF_018691265.1, Table 1). GC content was similar across haplotypes (41.9% each) and only slightly higher than in earlier assemblies (0.1% of increase compared to T.alba_DEE_v4.0). GC profiles were broadly homogeneous between chromosomes on average with a modest increase toward smaller chromosomes (Figure 1A, Figure 1B, Table 1, Supplementary Table 2).

BUSCO assessments indicated high completeness for both haplotypes (paternal: C = 99.4%, F = 0.10%, D = 0.18%, M = 0.61%; maternal: C = 99.4%, F = 0.08%, D = 0.21%, M = 0.62%), representing ~1% improvement over T.alba_DEE_v4.0 (C = 98.5%).

To assess the amount of potential contamination, we ran blobplot analyses [35] at the contig level (i.e., prior to scaffolding) for both the maternal and paternal haplome (Supplementary Figure 3 and 4). Both exhibited no evidence of contamination: most BLAST hits were assigned to *Tyto alba*, while a minor portion (tiniest contigs) corresponded to other avian species in both haplomes, as expected due to phylogenetic proximity [36].

To evaluate chromosome continuity, we quantified terminal features. In the paternal haplotype, 31 chromosomes contained telomeric sequence in 5p and 21 contained pericentromeric sequence. In the maternal haplotype, 34 and 18 chromosomes contained telomeric and pericentromeric sequence, respectively. Across haplotypes, 24 chromosomes were resolved from telomere to pericentromere, 18 contained only telomeric sequence, 2 centromeric sequence only, and 2 contained neither across haplomes (Figure 2A, Supplementary Table 3).

Repeat and gene annotation

Custom repeat annotation revealed that repeats constituted 13.68% (paternal) and 14.83% (maternal) of assembled sequence. LINEs were the predominant class (47.81% paternal and 47.07% maternal of total repeats), followed by LTR elements (32.23% paternal and 32.45%

maternal, Supplementary Figure 5). Repeats were enriched toward chromosomal ends and in the six smaller chromosomes. On the metacentric Z chromosome, repeat density peaked around the centromere (Figure 2A).

To investigate the proportion and types of repeats on the W chromosome, which is absent from the focal male assembly, we performed Fluorescence In Situ Hybridisation (FISH) on a female individual (Figure 2B). This technique uses fluorescent probes to localise specific DNA sequences on chromosomes, here targeting telomeric and centromeric repeats (see Material and Method section for details). The W chromosome exhibits strong signals for both probe types, suggesting a higher proportion of repeats in this chromosome compared to the rest of the barn owl genome.

Using the NCBI EGAPx workflow with multi-tissue RNA-seq support [33], we annotated 19,720 genes in the paternal haplotype and 20,039 in the maternal haplotype. These haplotype annotations were compared to the previous reference genome (T.alba_DEE_v4.0 - GCF_018691265.1) by mapping back Coding DNA Sequences (CDS) to quantify newly annotated CDS. Newly annotated CDS totalled 908 in the paternal haplotype and 948 in the maternal haplotype and were distributed over the genome, with an enrichment towards the smallest chromosomes (Figure 3D, Figure 3E).

Recovery and comparison of microchromosomes

To recover microchromosomes that were missing from the long-reads-based unitigs, we used MicroFinder [24]. This strategy allowed us to re-assign unplaced long-reads-based unitigs to specific microchromosomes and integrate them into the final haplotype assemblies.

We screened 116 unplaced unitigs in the paternal assembly and 119 in the maternal assembly. Based on conserved gene content, we identified 14 paternal and 18 maternal candidates using MicroFinder. We further assembled them in 6 new microchromosomes, that were absent from previous versions (RefSeq: GCF_902150015.1 and GCF_018691265.1).

In contrast to chromosomes already present in the previous assembly, the six newly recovered chromosomes are characterized by distinct genomic properties and separate clearly from all

remaining chromosomes in the PCA built from chromosomal features (Supplementary Figure 6). The recovered microchromosomes showed elevated GC content and most were extremely repeat-rich relative to larger chromosomes (Figure 3A, Figure 3B). They contained few genes in absolute terms and remained gene-sparse even after accounting for their size (Figure 3C, Figure 3E). Despite their sparse gene content, most of their annotated genes were absent from the prior assembly (GCF_018691265.1, Figure 3D, Figure 3E).

Haplotype synteny and a complex rearrangement on chromosome 7

Whole-genome alignment between haplotypes indicated extensive collinearity punctuated by a single, large and complex rearrangement on chromosome 7 (Figure 2A). Nearly the entire paternal chromosome 7 aligned in the opposite orientation to the maternal chromosome 7, with nested structure: a large (~20 Mb) inversion interleaved with two shorter inverted segments and separated by two collinear blocks, all translocated relative to each other (Figure 4A). Repeat density increased near inferred breakpoints in both haplotypes, supporting the potential evolution of successive rearrangements (Figure 4A) [37].

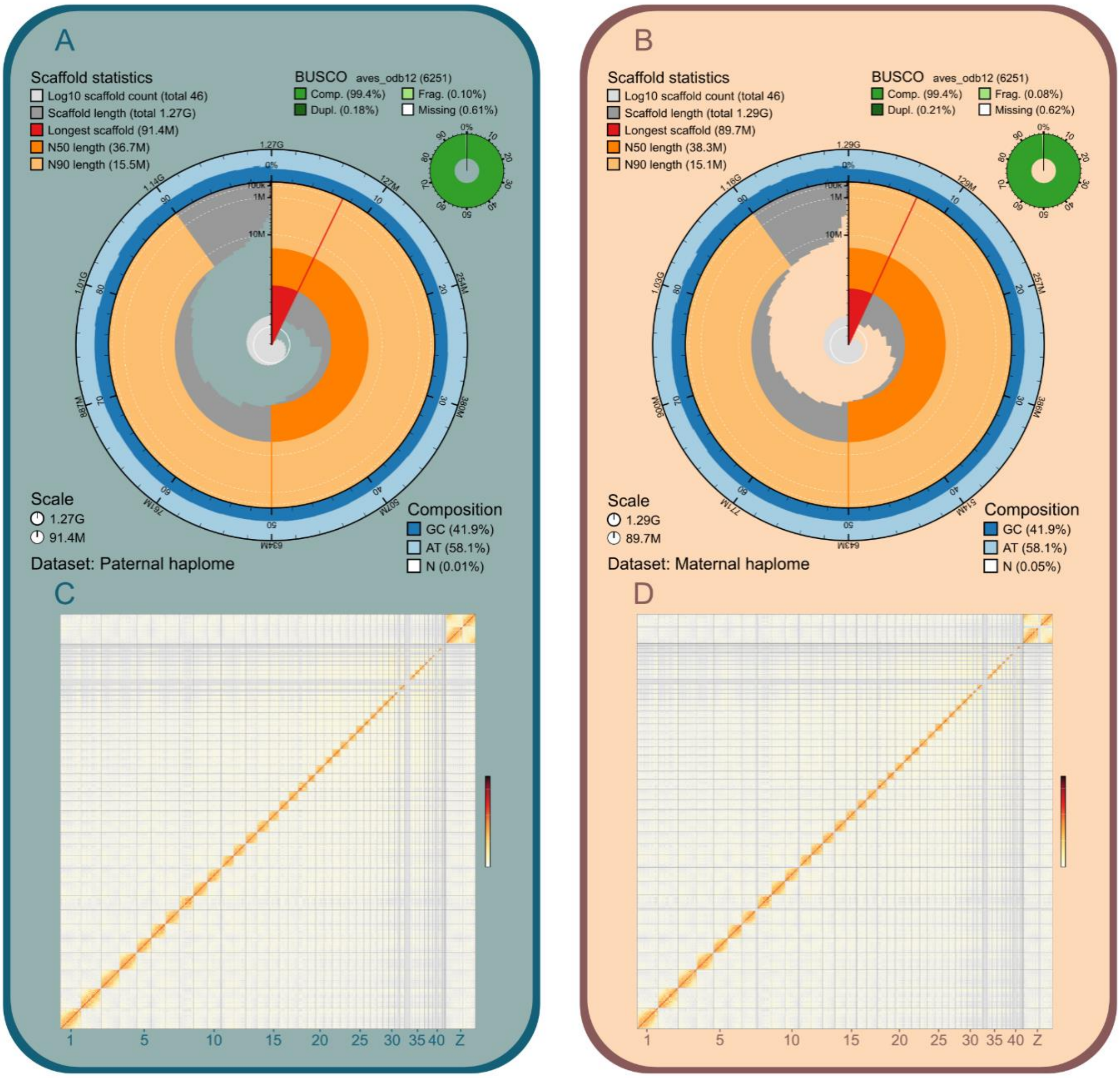

Figure 1 – Contiguity, completeness, and chromatin interaction landscape of the paternal and maternal haplotype assemblies. (A-B) Snail plots display the overall structure and composition of the (A) paternal and (B) maternal assemblies. Each circumferential axis is divided into 1,000 bins, each representing 0.1% of the corresponding assembly. Scaffold length distribution is shown in dark grey, with the plot radius scaled to the longest scaffold (highlighted in red). N50 and N90 scaffold length thresholds are indicated in orange and pale orange, respectively. Nucleotide composition is displayed as dark blue for GC, light blue for AT, and white for ambiguous bases (N). A BUSCO completeness summary (Complete, Fragmented, Duplicated, Missing) is provided in the upper-right corner of each panel. (C-D) HiC contact maps for the (C) paternal and (D) maternal assemblies illustrate the genome-wide pattern of spatial interactions. Chromosomes are arranged by size from left to right and from top to bottom, with the Z chromosome positioned last. The red main diagonal represents high-frequency intra-chromosomal contacts, while off-diagonal signals correspond to inter-chromosomal interactions. Interaction frequencies are displayed on a logarithmic scale. Heatmaps were generated using HiContacts.

Table 1 – Comparison of general assembly statistics across barn owl (*Tyto alba*) references.

	**genome_assembly_l500**	**T.alba_DEE_v4.0**	**Paternal**		**Maternal**	
	GCF_902150015.1	GCF_018691265.1	Prior to scaffolding [contigs]	After scaffolding [chromosomes]	Prior to scaffolding [contigs]	After scaffolding [chromosomes]
**Assembly size**	1,219,191,878	1,249,867,532	1,317,996,626	1,267,676,513	1,313,309,923	1,285,637,069
**# sequences**	21509	70	287	46	331	46
**N50**	4,615,526	36,032,128	26,240,345	36,723,072	24,830,383	38,271,680
**N90**	556,444	15,349,174	5,753,860	15,527,340	4,456,682	15,107,001
**% GC**	41.5	41.5	41.9	41.9	41.9	41.9
**Total Ns**	9,406,517	51,418,967	0	110,000	0	600,000
**% BUSCO**	98.4	98.5	-	99.4	-	99.4

Genomes compared include genome_assembly_l500 from Ducrest et al. (2020) and T.alba_DEE_v4.0 from Machado et al. (2021). These reference genomes are shown alongside the two parental haplomes assembled here. Haplomes are each shown twice: a first column for the unscaffolded contigs, raw output from hifiasm, and a second column for the chromosome-scale assembly after filtering, scaffolding and curation. Metrics include total assembly size, counts of sequences, N50/N90 (minimum length of sequences that cover a cumulative 50% or 90% of the entire assembled genome), percentage of GC, total Ns and BUSCO completeness.

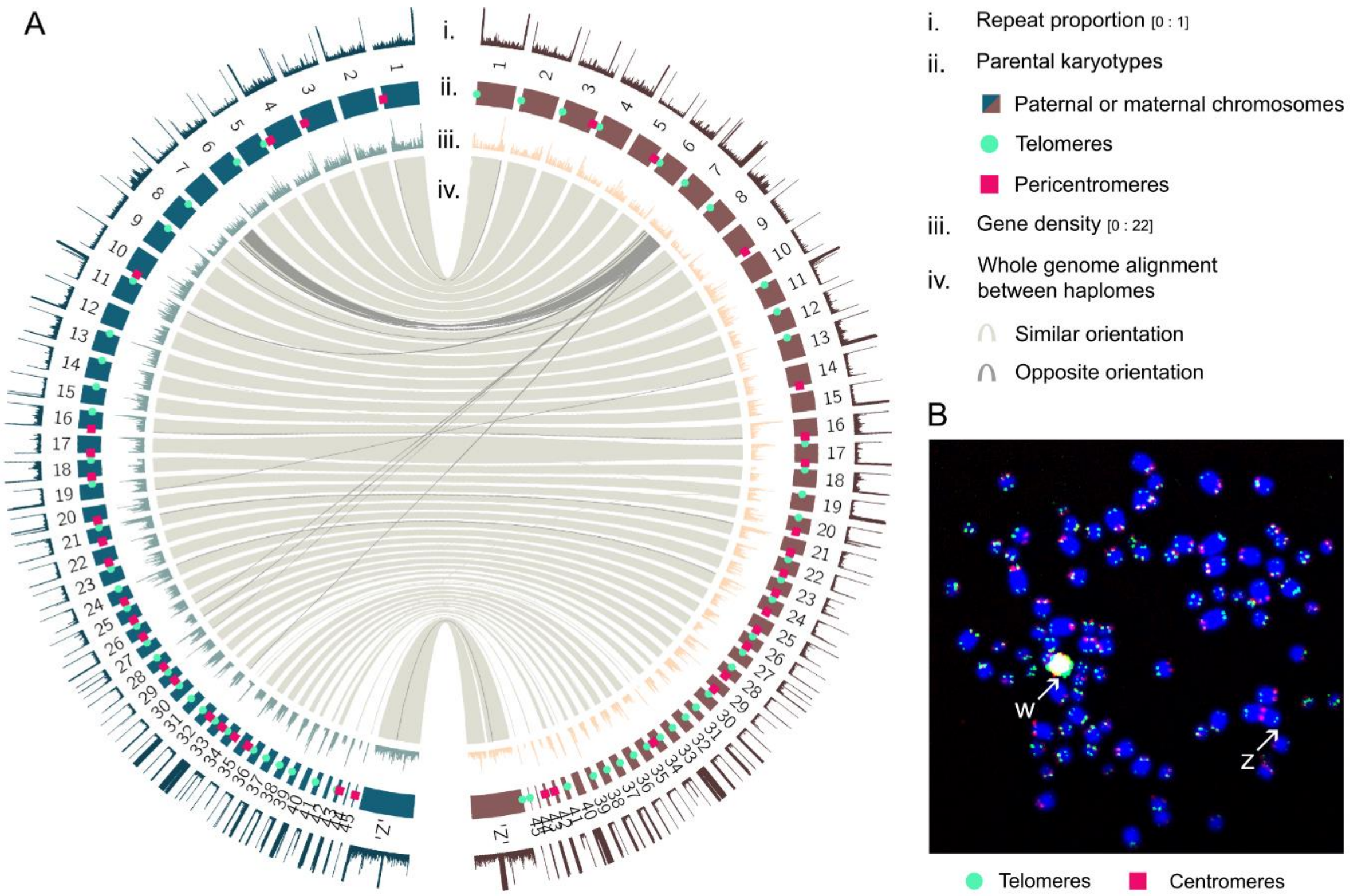

Figure 2 - Overview of the paternal and maternal haplomes and their genome features. (A) The Circos plot provides a comparative summary of the genomic characteristics of the paternal haplome (left, dark blue) and maternal haplome (right, burgundy). From the outer to the inner track, the plots show the repeat proportion calculated in 100-kbp windows; the 46 chromosomes of each haplome, with regions matching telomeric and pericentromeric sequences indicated by green circles and red squares, respectively; the distribution of annotated genes, summarised in 100-kbp windows; and the whole-genome alignment between the two haplotypes. Because repetitive elements strongly affect alignment, both haplomes were hard-masked with RepeatMasker prior to alignment. Regions aligning in the same orientation are shown in light grey, and regions aligning in opposite orientation are shown in dark grey. (B) Fluorescence in situ hybridisation (FISH) visualisation of the *Tyto alba* karyotype of a female individual. Chromosomes are counterstained with DAPI (blue). Centromeric regions are highlighted in magenta and telomeric regions in cyan using specific DNA probes (see Material and methods section for details). Sexual chromosomes Z and W are indicated by white arrows. FISH image in higher resolution can be found in Supplementary Figure 7.

215

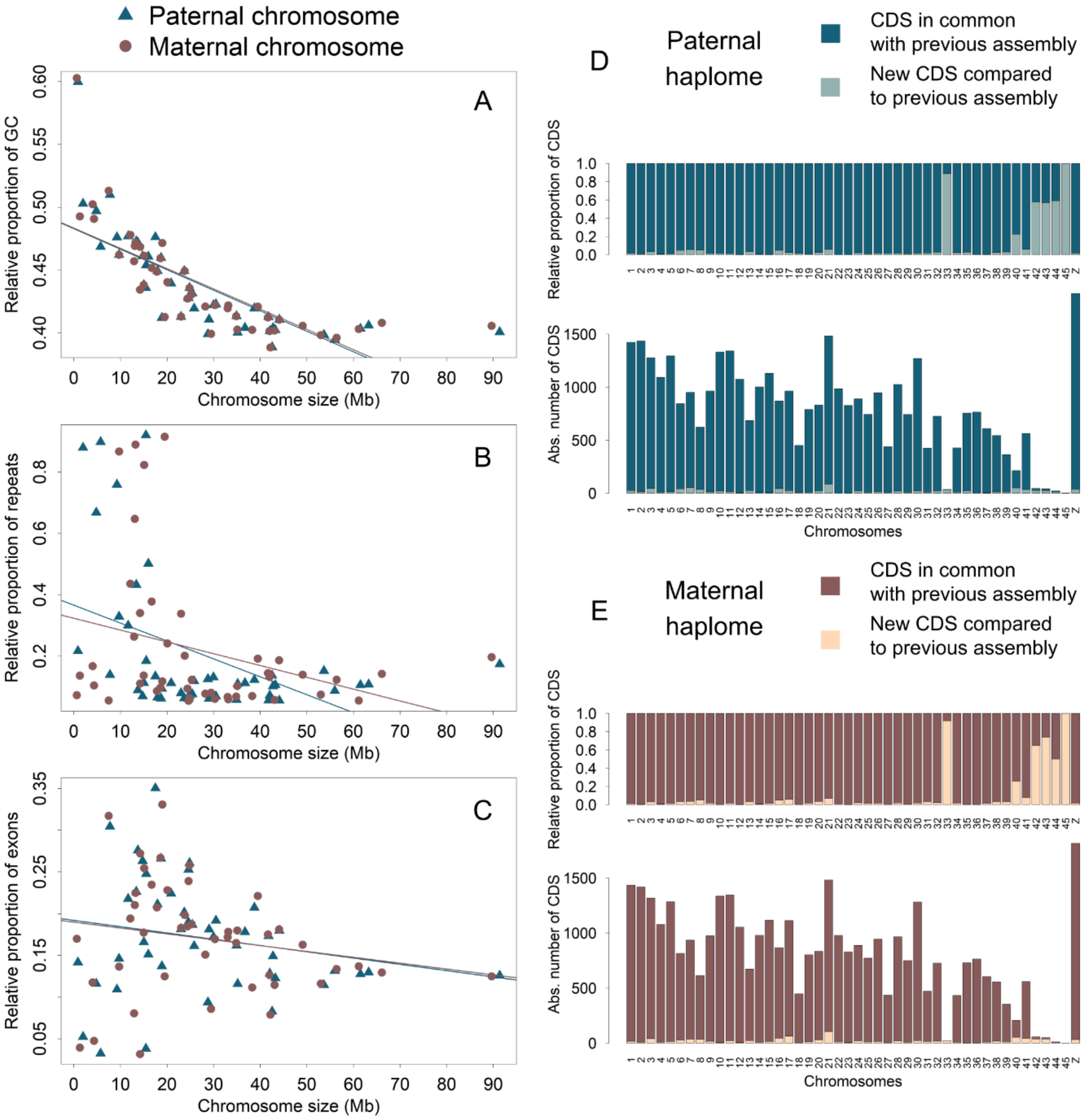

Figure 3 - Dot chromosomes and their genomic characteristics. (A-C) Chromosome-wide distributions of GC content (A), repetitive elements (B), and exonic sequence (C) are shown for the paternal (dark blue) and the maternal haplome (burgundy). (D-E) Bar plots illustrate the absolute (lower panels) and relative proportion (upper panels) of newly annotated genes on each chromosome of the paternal (D) and maternal (E) haplomes. Newly annotated genes compared with the previous assembly are depicted in lighter colours.

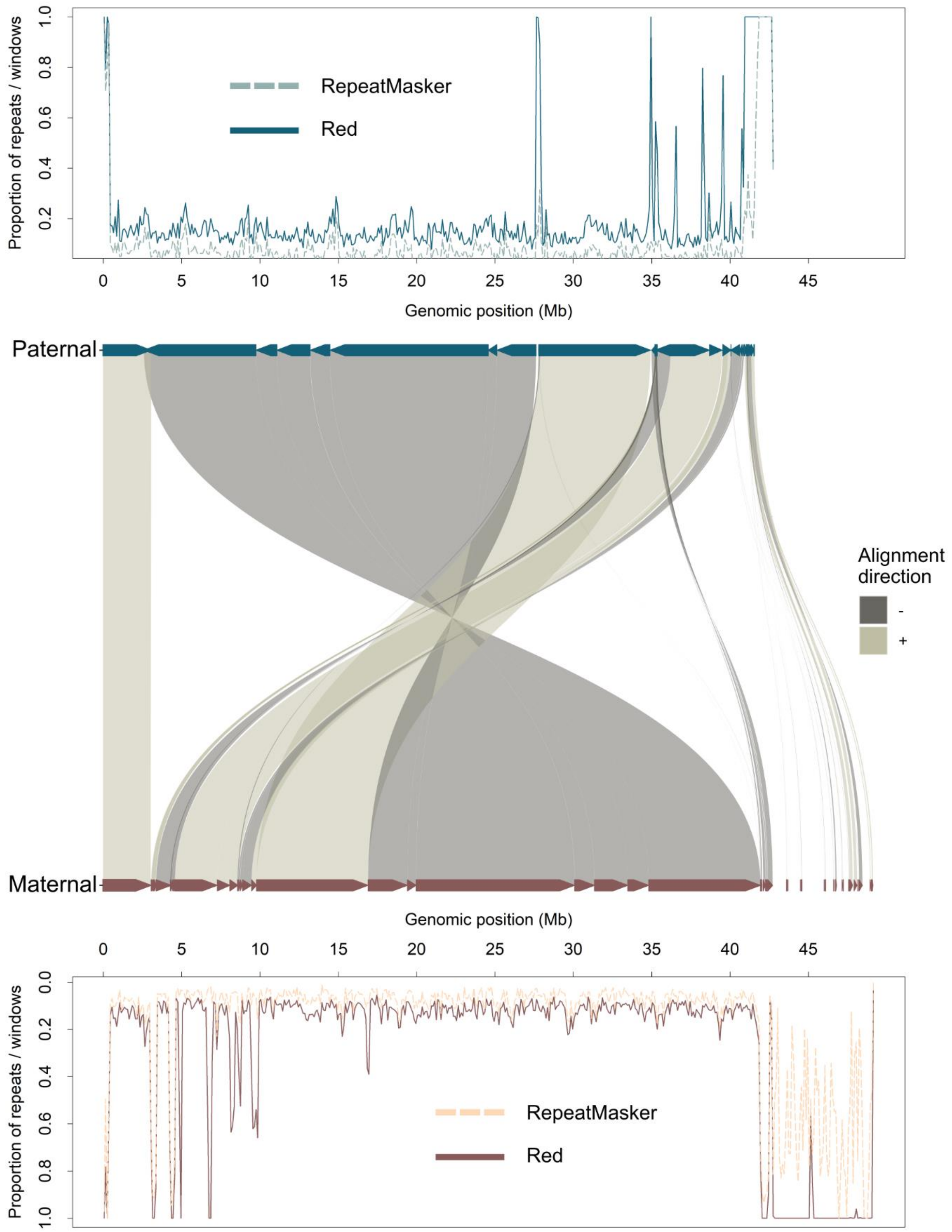

Figure 4 - Large complex inversion on chromosome 7. Alignment between the paternal (top, dark blue) and maternal (bottom, burgundy) chromosome 7 highlights a major chromosomal inversion. The figure shows an extract from the whole-genome alignment, restricted to alignment blocks (dark blue and burgundy arrows) that are exclusive to chromosome 7 in both haplomes. Apparent gaps, e.g., at the right end of the maternal chromosome 7, correspond to highly repetitive regions (as reflected in the repeat profile below) that align to other parts of the genome and are therefore not shown here. Regions aligning in the same orientation are displayed in light grey, whereas regions aligned in the opposite orientation are shown in dark grey. Repeat proportion, calculated in 100-kbp windows, is plotted along the top and bottom. Two repeat detection methods are shown: RepeatMasker, represented in a lighter colour with a dashed line, and Red, represented in a dark colour with a solid line.

Discussion

Recent advances in long-read sequencing technologies have made it increasingly possible to reconstruct near-complete, telomere-to-telomere chromosomes across an increasing range of species [1–5,8,11]. Yet birds remain challenging vertebrates to assemble owing to their numerous, small, GC-rich and repeat-dense microchromosomes, which have historically been under-represented or mis-assembled in reference genomes [7,23,24]. Here, we present high-quality haplotype-resolved, chromosome-level assemblies for the Western barn owl (*Tyto alba*), each comprising all 46 chromosomes expected from the karyotype [31,38] and substantially improving upon previous barn owl reference genomes [32,33]. These two haplotype-resolved assemblies exhibit high contiguity and completeness (with very few gaps and BUSCO scores above 99%) and recover six small chromosomes that were absent from earlier assemblies, providing a near-complete representation of the species' chromosomal assembly.

A multi-layered assembly strategy

No single sequencing technology was sufficient to resolve the barn owl genome. Instead, the assembly succeeded because multiple complementary resources were integrated, with each compensating for the others' blind spots. First, we phased the haplomes using Illumina reads from the parents, partitioning offspring long reads with trio aware *k*-mers before assembly to reduce switches and produce cleaner haplotype-specific contigs for downstream scaffolding. Then, HiFi contigs formed the structural backbone of the assembly, providing the accuracy and read length necessary to produce long, high-confidence unitigs. However, Hi-Fi reads alone cannot reliably span extremely GC-rich or repeat-dense regions, features abundant on avian micro- and dot chromosomes [39]. ONT ultra-long reads filled this gap, traversing repetitive sequences and bridging discontinuities between HiFi contigs, consistent with earlier reports demonstrating their unique value for difficult regions in bird genomes [25,26].

For chromosomes represented in the linkage map [34], population-based marker order provided a scaffold for organising HiFi / ONT contigs into linkage groups, after which Hi-C contact data confirmed chromosome-scale structure for the larger elements. However, Hi-C proved uninformative for microchromosomes: their short length and high repeat and GC content result in reduced mappability (Supplementary Figure 8), which in turn limits the reliable detection of meaningful contact patterns, a difficulty also encountered in other avian assemblies [24]. To identify and assemble these highly challenging chromosomes, we instead relied on conserved microchromosome gene-set signature [24]. This allowed to pinpoint unplaced unitigs enriched for microchromosome-specific genes. These fragments were then manually curated and stitched together, guided by ONT long-read alignments that provided the only long-range continuity through repetitive landscapes.

Altogether, the assembly demonstrates that the integration of all the available information was critical to construct a robust, near-complete chromosomal assembly. Our recommendation to facilitate good future assemblies would be to collect enough material to generate several HiFi libraries of different fragment sizes and collect enough ONT sequences, preferably as long as possible, before starting the assembly. Filtering of sequences containing high density of internal repeats, such as pericentromeric or centromeric reads, is also essential to prevent assembly artefacts. Although these regions are genuine components of the genome, as shown by FISH signals (Figure 2B), they are difficult to assemble reliably without dedicated approaches and can therefore introduce biases into the reconstruction of the rest of the genome.

Recovery and characteristics of the missing chromosomes

Bird genomes remain difficult to assemble fully, and only a handful of avian references currently include the entire set of microchromosomes [23,25,26,28,40,41]. However, synteny-based scaffolding is often insufficient for microchromosomes because they show rapid evolution and poor conservation of gene order across species [23,24].

We assembled six previously missing chromosomes for the barn owl, displaying hallmark features of micro / dot chromosomes (elevated GC content and enriched repeats) consistent with other avian genomes [25,26,28]. However, in contrast with the micro-chromosomes present in the previous assembly, they remain gene-sparse relative to expectations based on the classic view of microchromosomes as strongly gene-dense [23,24]. This pattern may reflect genuine biological variation in the barn owl lineage but also residual annotation challenges in small repeat-rich elements now referred to as dot chromosomes [42].

Based on our feature- based analysis, the distinction between macro- , micro- , and dot- chromosomes is not straightforward, and no single variable (e.g., length, GC content, or gene content) provides clear boundaries between these chromosomal categories. Future work should examine the functional significance of genes located on these newly identified chromosomes and explore whether particular gene families (such as immunity- or sensory- related genes) exhibit micro- / dot- chromosome–specific organization in owls.

Phased assemblies reveal complex structural variation

Fully phased haplotypes allow the precise characterisation of complex structural variation. Structural variants (SVs), such as large inversions, are increasingly recognised as important drivers of adaptation [43], phenotypic evolution [44], and recombination landscape heterogeneity [45], but their detection and interpretation require high-quality, often phased reference genomes [7,8,37,46]. In barn owls, previous indirect evidence suggested the presence of a large inversion linked to climate [47], but its structure and extent could not be determined with pseudo-haploid or fragmented references [32,33]. Our phased assemblies reveal a ~38 Mb complex inversion on chromosome 7, comprising multiple nested inverted and collinear segments. Yet the evolutionary history and potential phenotypic consequences of this inversion remain unknown. An important next step will be to examine its population frequency, heterozygosity patterns, and possible associations with traits such as coloration, dispersal, or behaviour traits that have shown geographic structure in barn owls [32].

Conclusions and perspectives

Together, our phased, chromosome-level assemblies provide a high-quality genomic resource for the barn owl and contribute to the growing set of genomic resources available for the order Strigiformes [30,32,33]. They reveal previously unknown chromosomes, expose the architecture of a complex chromosomal inversion, and shed new light on microchromosome characteristics. These resources create a strong foundation for investigating structural variation, recombination dynamics, and the genetic basis of phenotypic divergence in barn owls. When combined with long-term ecological and population-genomic datasets available for this species, they will enable new levels of biological inference and contribute to a broader understanding of avian genome evolution.

Material and methods

Sampling

To build our genome assemblies, we used the same reference individual as in Machado et al. (2022) [32], a Swiss male barn owl (M040663), and its two parents (Father ring ID: M032330; Mother ring ID: M028977). On the 9th of September 2019, we collected brachial-vein blood from each bird at a nestbox near Estavayer-le-Lac, Switzerland (46.85 N, 6.86 E). Blood samples were either kept at 4°C and processed rapidly for High Molecular Weight (HMW) DNA extraction with agarose plugs (see below) or snap-frozen in liquid nitrogen and transferred to −80 °C freezer for long term preservation.

High Molecular Weight DNA extraction of the reference individual

HMW DNA for the reference individual was extracted using an agarose plug method based on Zhang et al. [48] and previously described in Machado et al. (2021): fresh phosphate-buffered saline (PBS)-washed red blood cells were embedded in low-melt agarose plugs, digested with proteinase K, washed in EDTA, then in 10 mM Tris pH 8.0, 1 mM EDTA), melted, and digested with β-agarase to release the DNA. The resulting HMW DNA was dialysed in 10 mM Tris pH 8.0, kept at 4 °C, gently homogenised, quantified with Qubit (Thermo Scientific), and sized on Femto Pulse and pulsed-field gel electrophoresis. Mean fragment DNA size was around 100 to 150 kbp. These DNA was used for PacBio HiFi SMRTbell Express 2.0 and for one ONT library.

To increase DNA yield, we extracted new HMW DNA from the -80°C frozen blood using MagAttract kit (Qiagen, elution repeated up to 4x to get enough DNA) for future PacBio library preparation. Frozen blood was also used to prepare HMW DNA with the Monarch® HMW DNA Extraction for Tissue kit (New England Biolabs) for future ONT libraries.

Library preparation and sequencing

PacBio HiFi

The PacBio HiFi SMRTbell libraries were prepared and sequenced at the Lausanne Genome Technologies Facility (GTF, University of Lausanne). Briefly, HMW DNA was sheared (Megaruptor; Diagenode, Denville, NJ, USA) to ~20 kbp fragments and checked on a Fragment Analyser (Advanced Analytical Technologies, Ames, IA, USA). We prepared three SMRTbell libraries, two with SMRTBell Express 3.0 (10 µg DNA each) and one with Express 2.0 (5 µg), in accordance with the manufacturer's recommendations. After BluePippin size selection (>15 kbp; Sage Science, Inc., Beverly, MA, USA), libraries were sequenced on one and two SMRT 8M cells, respectively, using v2.2/v2.0 chemistry on a PacBio Sequel II instrument (Pacific Biosciences, Menlo Park, CA, USA) for a film run time of 30 hours and a pre-extension time of 120 minutes.

Oxford Nanopore Technology

Libraries were prepared from Monarch-HMW and plug-derived DNA using the ligation sequencing kit (SQK-LSK114). Sequencing was performed on PromethION flow cells R10 (FLO-PRO114M) using a PromethION 2 Solo instrument (Oxford Nanopore Technologies, Oxford, UK) at the Gene Expression Core Facility (GECF, EPFL, Switzerland).

For Monarch-HMW DNA, sequencing and base calling were carried out using MinKNOW software (v23.07.5) with the integrated Guppy base caller (v7.0.9) employing the super-accuracy (SUP) model. Plug-derived DNA libraries were sequenced using MinKNOW software (v25.03.9), and base calling was performed with Dorado (v1.0.0) using the SUP model, including duplex read analysis.

Hi-C crosslinking

Without thawing, we diluted 10 µL of -80°C frozen blood from the focal individual in 926.9 µL of PBS and mixed gently. We then added 63.1 µL of 10% formaldehyde and incubated 20 minutes at room temperature (mixing periodically). We quenched with glycine (final concentration of 125 mM), followed by a 15-minute incubation at room temperature. We stored

the sample at −80 °C and sent frozen to Phase Genomics (United States) for Hi-C library preparation. Sequencing was performed at the GTF (University of Lausanne, Switzerland).

DNA extraction and sequencing of the parents

Following Cumer et al. (2025) [49], we extracted genomic DNA from whole blood with the DNeasy Blood & Tissue kit (Qiagen, Hilden, Germany). We prepared Nextera DNA libraries (Illumina) and sequenced multiplexed libraries on a NovaSeq 6000 (PE high throughput) at the GTF (University of Lausanne, Switzerland). This sequencing of the parents yielded to a total of 172,731,951 and 93,360,939 read pairs for the father and the mother respectively.

Initial trio assembly

Taking advantage of trio information, we used hifiasm v0.19.8-r603 in trio mode to generate phased haplotype assemblies. Parental k-mers were first computed using yak (version 0.1-r93-dirty). Because parental sequencing depth was uneven (~38× for the father and ~21× for the mother, for an estimated genome size of ~1.3 Gb), we followed developer recommendations and relaxed the lower count threshold for the maternal dataset (yak option -l2) to retain informative low-frequency k-mers.

We then ran hifiasm using all PacBio HiFi reads, together with Oxford Nanopore long reads (>25 kbp), and combined with the two parental k-mer databases. To improve the resolution of repetitive regions, we increased the k-mer frequency cutoff parameter to -D 10. This more permissive threshold prevents over-filtering of moderately repetitive but informative sequences and improving graph connectivity during assembly.

This procedure resulted in a highly unbalanced phased assembly, with a paternal assembly of 1,755,937,883 bp (GC = 46.3%) and a maternal assembly of 1,207,411,275 bp (GC = 42.7%).

Unequal haplotype representation in hifiasm assemblies is a known issue that can arise when the homozygous coverage is incorrectly inferred, in which case the --hom-cov parameter can be adjusted to better separate homozygous and heterozygous k-mer peaks. However, in our

dataset, the inferred homozygous coverage (~119×) closely matches the expected value based on the k-mer spectrum, with a clear and well-separated heterozygous peak (~58×), indicating that coverage misestimation is unlikely to explain the observed imbalance.

We therefore hypothesised that the imbalance instead originates from an excess of highly repetitive reads, which can bias overlap selection and graph construction, ultimately leading to uneven haplotype resolution. To address this, we implemented an additional filtering step in which reads were stratified based on repetitiveness and highly repetitive reads were selectively removed prior to reassembly.

Identification of repeated sequences and HiFi read classification

To reduce assembly artefacts from extreme repeats, i.e., reads composed almost entirely of highly repetitive sequences, we first counted 64- mers in HiFi reads with Jellyfish v2.3.0 (-m 64 -C -c 3 -t 32 -L 50 -s 9,717,986). There were 1,793,627 64- mers occurring >1,000x. These 64-mers were offsets of the same patterns and carried several instances of smaller sub-motifs. For example, the six most frequent k- mers (each, >1.5 M) carried several instances of the sub- motif GTCACGTGGTC. The next four (each > 1.3 M) carried TTAGGGTTAGGGTTAGGG (further categorised as telomeric repeats). We further inspected reads containing highly repeated motifs and noticed some were covered by multiple instances of identical sub-motifs, which we decided to filter prior to the assembly. Based on these observations, we placed HiFi reads into five categories based on the presence of the following motifs scanned in forward and reverse complement orientation:

- *Centromere-like*, characterised by at least 1 occurrence of the sequence CAGCACTGTTTCCTCCAAACTACCATGCTGCGCATAGCAATCCCTGTCTAGCTG CTTCCTTTCACTGGGAACACA, and labelled as *centromeric-like* due to the high conservation of a 162pb long repeat, compatible with a centromeric sequence [50]. This classification was further confirmed by the FISH experiment, see the *Karyotyping and FISH of telomeric and centromeric sequences* section below,

- *Kinetochore-like / pericentromeric repeat,* characterised by at least 3 occurrences of the sequence of the motif GTCACGTGGTC, labelled as such since these repeats are frequently found at extremities of reads composed of centromere-like repeats.
- *Telomere-like,* characterised by at least 3 occurrences of the motif TTAGGGTTAGGGTTAGGG, composed of multiple repeats of the vertebrate telomeric motif (TTAGGG) [51],
- *Microsatellite*, characterised by at least 1 occurrence of the sequence ACCTTCCCCCCACCCCTCCCTTCTTCCCGGGCTCAGCTCTGCTCCCGATATCTC TACCTCCTCC and labelled as *microsatellite* since this sequence has been previously described and was used to genotype microsatellite markers in the barn owl [52],
- Other (none of the above).

We excluded centromere-like and kinetochore-like reads since they contained repeated motifs spanning entire reads of length over 20 kbp but retained telomere-like (since they are at the end of chromosomes and less likely to confuse the assembler), and microsatellite-like whose repeated motif was present only in parts of the reads. This yielded a total of 7,826,051 retained HiFi reads, that were subsequently sorted by parental allele (see next section).

Trio assembly excluding centromeric-like and kinetochore-like sequences

Based on this classification, we performed a second assembly using hifiasm v0.19.8-r603 in trio mode with the same parameters as described above in "*Initial trio assembly*", but excluding reads classified as centromeric-like and kinetochore-like. The assembly was conducted using the filtered set of HiFi reads , filtered Oxford Nanopore long reads (>25 kbp) and parental k-mer databases.

This procedure resulted in a smaller yet still unbalanced phased assembly, with a paternal assembly of 1,474,597,196 bp (GC = 41.7%) and a maternal assembly of 1,196,923,645 bp (GC = 42.1%). Notably, the GC content between haplotypes became more consistent, suggesting that the removal of highly repetitive reads reduced sequence composition biases associated with repeat-rich regions.

In hifiasm trio mode, reads are internally partitioned into parental haplotypes based on k-mer matches derived from parental sequencing data. While this approach is generally effective, its accuracy may remain limited in moderately repetitive sequence and low-divergence allelic regions that can still share k-mers between haplotypes, reducing the discriminative power of k-mer–based assignment.

In addition, the unequal sequencing depth between parental datasets (~38× vs ~21×) may lead to asymmetries in parental k-mer representation, potentially biasing read assignment toward the more deeply sequenced parent despite the use of relaxed filtering parameters.

Thus, given the persistence of haplotype imbalance after repeat filtering, we hypothesised that residual misassignment or ambiguity in read partitioning affect assembly quality. To further improve haplotype resolution, we therefore explicitly partitioned HiFi reads by parental origin prior to assembly and performed independent assemblies for each haplotype.

Allele sorting of HiFi reads

With the final intention to build haplome specific assembly, we sorted HiFi reads according to the parent of origin using a k-mer based approach that identifies parent-specific kmers in each read.

We first build parent-specific sets of k-mers from parental Illumina reads. To balance uneven parental sequencing depths (Father 172’731’951 reads vs Mother 93’360’939 reads) and sampling bias of kmers during sequencing, we randomly drew non-overlapping subsets of 46,680,469 Illumina paired-end reads, 3 for the Father and 2 for the Mother. In each of these five sets, we counted 49-mers (length chosen to avoid palindromes and reduce homopolymer errors; Supplementary Table 4), free of Ns. Finally, we defined nine parent specific k-mer libraries containing 49-mers present ≥ 2x within a parental subset and absent from pairs of subsets of the other parent (see Supplementary Table 5). These parent specific k-mer libraries allowed to account for sampling bias and statistical testing during the read classification procedure described in the next paragraph. This comparison between parental sets was done using a modified jellyfish v2.3.0 to emit k-mer “bin” files unique to one library and absent from

a given set of other libraries (patch provided on the GitHub, see Availability of Source Code and Requirements section for details.

We sorted the HiFi reads of the reference individual into two parental sets. Each HiFi read was scanned with a sliding window of 49- mers, using a 7- nt step to keep the computational burden manageable. For each read, we counted the occurrences of parent- specific k- mers. This counting procedure was performed for every pairwise combination of the parent- specific k- mer libraries, generating a distribution of k- mer counts for each parent for each read. Based on these distributions of counts, we assigned reads to parental origin using paired t- tests with Benjamini–Hochberg FDR control implemented in R. Reads with FDR ≤ 0.001 were assigned to the parent with the highest number of matching k- mers. Unassigned reads were rescanned using a 1- nt step sliding window, and the assignment test was repeated using an FDR threshold of ≤ 0.05. FDR control was performed separately within each read class (telomere- like, microsatellite- like, other) to account for compositional biases associated with these sequence categories (Supplementary Table 6).

ONT reads filtering, classification and allele sorting

For ONT reads, we selected all reads generated by the ONT caller that were 25 kbp nucleotides or longer. ONT reads were then attributed to the five categories described earlier (*Centromere-like, Kinetochore-like / pericentromeric repeat, Telomere-like, Microsatellite, other*) using the motifs identified with HiFi reads. The allelic sorting process explained in the previous section was applied to the ONT run 1 and 2 (≥ 25-kbp long) using a sliding window offset of 7nt and an adjusted p-value ≤ 0.05 to assign predicted parental origin.. Detailed results after the filtering, classification and sorting are presented in Supplementary Table 7.

Haplotype-specific assembly and scaffolding

HiFi-based assemblies

We ran hifiasm 0.19.8-r603 to build separate maternal and paternal haplotype using: (i) HiFi reads assigned to that parent, (ii) all HiFi reads from uncertain origins and (iii) ONT from run

1 (ONT run 2 not available at that stage). After the initial assemblies and to detect regions with abnormally high parental coverage, we realigned HiFi reads to the draft assemblies with minimap2 2.26-r1175. After realigning the reads to both parental haplotypes, we assigned each read to the parent with the highest alignment score. After that step we identified regions where the coverage was <=20% and >=80% to the expected coverage. Reads contributing to these imbalances were identified, sorted by increasing adjusted p-value from the read sorting step (see above for details) and the bottom half of them (i.e. worse adjusted p-values) re-assigned to the other parental allele, implementing the idea that the increased coverage is due to wrongly assigned reads. After this final reclassification of 14,717 reads, we generated two final haplotype assemblies using hifiasm 0.19.8-r603. The final drafts comprised 287 paternal and 331 maternal unitigs.

ONT assemblies

Using hifiasm 0.25.0-r726, we independently assembled ONT reads into parental haplotype (inputs: ONT runs 1 and 2 assigned to that parent + uncertain-origin ONT reads from runs 1 and 2), yielding 1030 paternal and 985 maternal unitigs

Final scaffolding with linkage groups and ONT-based assemblies

We first scaffolded the HiFi-based contigs into 40 linkage groups using the linkage map produced by Topaloudis et al. [34]: we extracted ±100 bp around each marker from the old assembly to create pseudo-reads and mapped them to the new haplotype assemblies, thus ordering unitigs by marker order and coordinates.

Then, we mapped all HiFi reads to their HiFi unitigs to flag low coverage regions of interest (ROI). Using ad-hoc scripts and aligners (minimap2, LASTZ, MAFFT), we stitched HiFi and ONT unitigs across ROIs and unitig ends guided by the linkage map, to produce the final scaffolds for both haplotypes. Stitched scaffolds were spaced by stretches of 10’000 Ns. Because owl chromosomes are acrocentric (Figure 2B) [31,53], and we excluded centromere-

rich reads, we oriented all scaffolds with a 5’ telomere (e.g.,5p-CCCTAA-3p repeat) at the p arm so that adding centromeres later will not alter the coordinate system.

Recovery of micro-chromosomes

Identification of the microchromosomes

To recover the unscaffolded microchromosomes in both assemblies, we used MicroFinder [24]. This tool compares the gene content of unplaced scaffolds against a curated set of genes known to be enriched on microchromosomes across avian species, flagging candidates showing significant enrichment. Following the authors' recommendations, we set a maximum scaffold size cutoff of 5 Mb to focus the analysis on short, genuinely unplaced sequences and retrieved 14 and 18 micro-chromosome contigs for the paternal and the maternal assemblies, respectively.

Assembly of the microchromosomes

Candidate unitigs identified in the *Recovery of microchromosomes* section were then manually assembled into six additional microchromosomes. This manual curation was done using ad-hoc scripts and aligners (minimap2, LASTZ, MAFFT) and supported by sequence alignment comparisons between the maternal and paternal haplotypes as well as using the unitigs produced by HiFi and ONT assemblies. Stitched unitigs were spaced by stretches of at least 10 kbp of Ns.

Detection of terminal features of the chromosomes

We assessed whether chromosomes were assembled completely (i.e., spanning telomere- to- centromere for autosomes or telomere- to- telomere for the Z chromosome). Because centromeric and pericentromeric reads were excluded during assembly, we realigned the raw long-read data to both the maternal and paternal assemblies to identify reads mapping to chromosome ends and containing telomeric or centromeric/pericentromeric

repeats. Both PacBio HiFi (Sequel IIe, chemistry M64) and Oxford Nanopore Technologies long reads were mapped against each reference. Each read was then assigned to a genomic feature class (telomere, centromere, kinetochore, microsatellite repeat, or "other") based on the presence of class- specific sequence motifs (see Identification of repeated sequences and HiFi reads classification). Only primary alignments were retained (filtering on tp:A:P), and a per- read mapping proportion was computed as the number of matched bases divided by the total read length.

Reads mapping near chromosome termini (where telomeric and centromeric repeats are expected) were identified by selecting those with alignment start positions within 10 kbp of the chromosome beginning or alignment end positions within 10 kbp of the chromosome end. Terminal- proximal reads were further filtered to retain only those with mapping quality >10 and mapping proportion >5%. Finally, reads not classified as telomeric, centromeric, or kinetochore-like were removed.

Finally, reads were grouped by parental haplotype, chromosome, terminus (start or end), and feature class. Clusters supported by fewer than 15 reads were discarded. Because all scaffolds were oriented based on the presence of telomere- specific repeats (e.g., 5′- CCCTAA- 3′), we then examined the distribution of read clusters along each chromosome. Specifically, we inspected autosomes for clusters of telomeric reads at their chromosome starts, and the Z chromosome for telomeric clusters at both termini. Conversely, we assessed chromosome ends for the presence of centromeric or pericentromeric read clusters, consistent with expectations for scaffold boundaries corresponding to centromeric regions.

Protein-based synteny across avian species

To confirm the identification of the Z chromosome, we aligned chicken (GCF_016699485.2) and zebra finch (GCF_048771995.1) proteins to both haplotype assemblies using miniprot (v0.18-r281) [54]. We kept alignments with ≥ 70% sequence identity and ≥ 90% target coverage, then merged pairwise mapping formats (PAFs) with reference feature tables to

count matches per reference chromosome vs barn owl chromosome. Sankey diagrams summarised chromosome-level correspondences (Supplementary Figure 1 and 2).

Evaluation of the phasing of the assemblies

To avoid biases caused by differences in parental sequencing depth, we down-sampled parental Illumina reads to 80,000,000 per parent using seqtk (v1.4) with a fixed seed (-s 100) for reproducibility and counted 49-mers with meryl *count* (v1.4.1). We derived parental-private k-mers (meryl *difference*), counted 49-mers in each assembly, and compute a phasing error as: the number of misplaced parental-private k-mer in the wrong haplotype divided by the total number of parental-private k-mers across both haplotypes.

Genome quality control

Quality and completeness were evaluated at two stages of the assembly process: the contig level (prior to scaffolding) and at the chromosome-level of the final parental assemblies. At both stages, we assessed completeness with BUSCO v6.0.0 (aves_odb12) [55] and used Quast v5.3.0 [56] to compute the general assembly statistics (sequences counts, N50/N90, GC, gaps). Assembly completeness and contiguity were visualised with BlobTools2 snailplots [35].

Genome contamination was also evaluated at the contig level for the parental assemblies using BlobToolKit v4.5.3 [35]. HiFi reads were aligned to the maternal and paternal assembly FASTA sequences using minimap2 [57] (v2.24-r1122) to generate BAM files for coverage estimation. Taxonomic assignments were obtained by aligning assembly FASTA sequences against the NCBI nucleotide (nt) database (July 2021 release) using BLASTN [58] (v2.14.0; parameters: -outfmt '6 qseqid staxids bitscore std' -max_target_seqs 10 -max_hsps 1 -evalue 1e-25). Blob plots were generated using species-level taxonomic assignments (bestsumorder_species) and sequence coverage information. The resulting plots were visually inspected for contigs with taxonomic assignments inconsistent with birds, which could indicate

potential contamination. Finally, whole-genome synteny was assessed by aligning the maternal haplome to the paternal haplome using minimap2 (-x asm10) [57].

Repeat annotation

We identified the repeats with two complementary approaches: (i) Red (de novo, library free) [59] and (ii) RepeatModeler v2.0.4 [60] with default parameters to build a custom repeat library, followed by RepeatMasker v4.1.5 [61] with default parameters for classification and masking of the repeats.

Gene annotation

We annotated both haplomes with NCBI Eukaryotic Genome Annotation Pipeline (EGAPx) [62]. In brief, EGAPx takes a genome assembly in FASTA format, a taxonomy ID, and RNA-seq data as input. First, it uses protein homology by aligning protein sequences from closely related species with prosplign. Second, it uses transcriptome information from the focal species. We included RNA-seq short reads from six tissues (liver, heart, kidney, testis, growing back feathers and thalamus) previously published in Ducrest et al. [33], which EGAPx aligns with STAR [63] and minimap2 [57]. Third, EGAPx runs Gnomon to generate *ab-initio* predictions [64]. These different hits are then integrated by Gnomon to produce a final consensus annotation.

We realigned Coding DNA Sequence (CDS) from the EGAPx annotation to their respective haplome with minimap2 in split mode [57] and kept only full-length mapping, MAPQ = 60. These quality-filtered CDS were then mapped back to the previous reference genome (T.alba_DEE_v4.0 - GCF_018691265.1) to classify CDS as previously present (full-length, MAPQ = 60) or new. For multi-mapped CDS, we retained the alignment with the longest aligned span and summarised counts per chromosome.

Chromosome characteristics and classification

To distinguish macro-, micro-, and dot chromosomes, we ran a PCA on z-scored, chromosome-level features: (i) length, (ii) total exonic length (relative), (iii) GC, (iv) gene density, (v) exon density, (vi) mean exon length; (vii) fraction of simple/low complexity repeats and (viii) fraction of other RepeatMasker classes (resolving overlaps by the larger element's class).

Genome mappability masking

Genome-wide mappability was quantified using the seqbility/SNPable pipeline (see Availability of Source Code and Requirements section for details). The reference genome was first decomposed into all possible k-mers using the splitfa program, with the k-mer size of 150bp, matching common read length. These k-mers were then aligned back to the reference using BWA in aln mode [65]. To match the SNPable specification, we used several non- default BWA parameters to allow permissive and sensitive mapping: the seed length was reduced (-l 32), up to two seed mismatches were permitted (-k 2), and both the gap- open (-O 3) and gap- extension (-E 3) penalties were increased relative to standard BWA defaults.

The resulting SAM file was processed with gen_raw_mask.pl, which extracts, for each k-mer, the number of perfect and near-perfect alignments it produces across the genome. This raw mask was then summarised into position-wise mappability tracks using the gen_mask script. For this step several non-default stringency thresholds were applied (-r 0.50, 0.90, 0.95, and 0.99). These thresholds represent the minimum fraction of overlapping k-mers that must map uniquely for a genomic position to be considered mappable. Lower thresholds generate more permissive callable regions, whereas higher thresholds identify only the most confidently unique regions.

Finally, we quantified the proportion of mappable regions per chromosome at different stringency thresholds (0.50, 0.90, 0.95, and 0.99), calculated as the ratio of the cumulative length of mappable regions to the total chromosome length.

Recombination map

We lifted the coordinates of the linkage markers from [34] by extracting 200-bp around each SNP with bedtools *getfasta* (v2.x) and mapping these sequences to each haplome (bwa-mem v0.7.x, sorted with samtools v1.x). We kept only SNPs with a unique and perfect alignments (MAPQ = 60), and for each combined the new physical coordinates with the original linkage-map metadata (linkage group and genetic position) to lift the recombination maps coordinates in each haplotype coordinate system.

Hi-C contact maps

We sequenced 338,385,433 Hi-C pairs. After enzyme-site filtering for GATC at R1 ends and R2 starts, 112,239,344 paired reads (33.2%) remained. We aligned reads with FetchGWI [66], converted alignments to genomic pairs, and built .cool files with cooler v0.9.3 (cooler *cload pairs* c1 1 -p1 2 -c2 3 -p2 4), followed by cooler *zoomify*. Genome-wide maps were generated at 16-kbp wide bins.

Karyotyping and FISH of telomeric and centromeric sequences

Cell culture and chromosome preparation

On the 10th of July 2025, we collected three growing breast feathers from a 28-day-old female nestling (M046151). After surface sterilisation (70% ethanol) and PBS rinses, we dissected the feather basal parts, scraped inner tissues using tweezers, added it to a 24-well plate containing Dulbecco's Modified Eagle's Medium high glucose (DMEM, Avantor) with 20% foetal bovine serum (FBS, Sigma), 1 % penicillin-streptomycin (Thermo Scientific), 5% AmnioMax II (Gibco, Thermo Scientific) and the MycoGenie MycoPlasma Elimination Kit (1:2000; Assay Genie, Chemie Brunschwig). Cultures were maintained at 37°C with 5% CO_2 for ≥ 3 weeks to obtain enough cells.

To arrest mitosis, we treated cells with KaryoMax Colcemid (0.5 µg/ml, 1h; Thermo Scientific), followed by 0.075 M KCl hypotonic shock (20 minutes, 37°C) and gentle fixation in cold freshly

prepared methanol/acetic acid (3:1) with 3-4 wash/fix cycles. The metaphase chromosome suspensions were stored at -20°C.

Hybridisation

We spread 10 µl of metaphase suspension per slide. After aging (1h, 65°C) and a 2x SSC wash (10 minutes, 300 mM sodium chloride, 30 mM sodium citrate, pH 7.0) at room temperature, slides were incubated with 100 µg/ml RNAse A (Qiagen) in 2x SSC for 45 minutes at 37°C. The slides were then washed in 2x SSC for 5 minutes at room temperature and treated with 0.005% pepsin for 3 minutes in 0.01 N HCl heated to 37°C. We then washed in PBS with 50 mM $MgCl_2$, denatured them in 1% paraformaldehyde (Thermo Scientific) for 10 minutes at room temperature, washed with 2x SSC, and dehydrated using a series of 70%, 90%, and 100% ethanol and let them dry for 20 minutes at room temperature. We hybridised the slides with 1 pmol of CEN3.2_ATTO590 centromeric probe (5'-TTTCCTCCAAACTACCATGCTGCG-3' labelled with ATTO590 at the 5' end, see the *Centromere-like* section above for the identification of this sequence) in 20 µl containing the PNA telomere hybridisation buffer with 10 µl of Telomere PNA FITC probe (Telomere PNA Kit/FITC for Flow Cytometry, PNA FISH Kit/FITC, Agilent Technologies). Coverslips were sealed on the slides, the slides denatured (92°C, 3 minutes) and hybridised overnight (37°C). The slides were then washed twice in 2x SSC + 0.1% Tween 20 (60°C, 15 minutes), and once in 0.2x SSC (room temperature), then mounted with Vectashield mounting medium + DAPI (500 ng/ml; Sigma). Imaging used a Leica Stellaris5 confocal microscope equipped with a 63xPlanApo oil immersion objective (CIF platform, University of Lausanne). All acquisitions were produced by superimposing focal planes. Post-processing, including pseudo colouring, was performed using Fiji [67].

Authors contribution

H.C.: conceptualisation, data curation, formal analysis, validation, investigation, visualisation, methodology, writing (original draft; review & editing). A.L.D.: conceptualisation, resources, formal analysis, investigation, visualisation, methodology, writing (review & editing). M.B.S.: data curation, formal analysis, visualisation, writing (review & editing). A.B.: data curation, formal analysis, visualisation, writing (review & editing). A.T.: data curation, writing (review & editing). C.S.: Resources, writing (final revision). E.C.: Resources, writing (final revision). D.C.: Resources, writing (final revision). B.A.: Resources, funding acquisition, writing (final revision). A.R.: Resources, funding acquisition, writing (final revision). C.I.: conceptualisation, data curation, software, formal analysis, validation, visualisation, methodology, writing (review & editing). N.G.: conceptualisation, data curation, software, formal analysis, validation, investigation, visualisation, methodology, writing (review & editing). T.C.: conceptualisation, data curation, formal analysis, supervision, validation, investigation, visualisation, methodology, writing (original draft; review & editing). J.G.: conceptualisation, supervision, funding acquisition, methodology, writing (review & editing)

Funding

This work was supported by the Swiss National Science Foundation with grants 310030_215709 to J.G.

Ethics Approval

Samples were collected under Swiss veterinary permit 35712 (VD3884) for the long-term monitoring of the Swiss barn owl (*Tyto alba*) population. All sampling procedures were performed in accordance with the conditions specified in the permit.

Data availability

Assemblies are available under the Umbrella BioProject PRJNA1444161 (Paternal assembly: PRJNA1440222, Maternal assembly: PRJNA1440227). The data underlying this article are available under the BioProject PRJNA1444114. FASTA sequences, repeat annotations, gene annotations, recombination maps, and mappability masks for both haplotypes are available on Zenodo [68]. The source data underlying Figures 1-4 and Supplementary Figures S1-S8 have also been deposited on Zenodo [69].

List of Abbreviations

b: bases; BAM: Binary Alignment Map; bp: base pair; BLAST: Basic Local Alignment Search Tool; BUSCO: Benchmarking Universal Single-Copy Orthologs; CDS: Coding DNA Sequences; Gb: gigabases; FDR: False Discovery Rate; FISH: Fluorescence In Situ Hybridisation; Hi-C: high-throughput chromosome conformation capture; HiFi: high-fidelity; HMW: High Molecular Weight; k: kilo; LINE: long interspersed nuclear element; LTR: long terminal repeat transposable element; NCBI EGAP: National Center for Biotechnology Information Eukaryotic Genome Annotation Pipeline; ONT: Oxford Nanopore Technologies; PCA: Principal Component Analysis; T2T: telomere-to-telomere

Competing interests

The authors declare that they have no competing interests.

Acknowledgement

We thank the entire field team of the groups of Alexandre Roulin and Bettina Almasi for their contribution in collecting samples. In particular, we are grateful to Roman Bühler, Nicolas Sironi, Kim Schalcher, Nathan Külling, Alexandre Roland, Nathan Joye, and Ilou Lejeune and

previous field assistants for collecting the blood and feather samples used in this study. We also acknowledge the Genome Technologies Facility (GTF) at the University of Lausanne and the Gene Expression Core Facility (GECF) at EPFL for their technical guidance and expertise during sequencing.

Availability of Source Code and Requirements

Project name: TytoAlba_NewReferenceGenome

Project home page: https://github.com/hugocrvl/TytoAlba_NewReferenceGenome

License: GNU GPL 3 license

Operating system(s): Linux

Programming language: Perl, Shell, R, Jupyter Notebook, Python.

References

1. Nurk S, Koren S, Rhie A, Rautiainen M, Bzikadze AV, Mikheenko A, et al.. The complete sequence of a human genome. *Science*. 2022; doi: 10.1126/science.abj6987.

2. Chen J, Wang Z, Tan K, Huang W, Shi J, Li T, et al.. A complete telomere-to-telomere assembly of the maize genome. *Nat Genet*. 2023; doi: 10.1038/s41588-023-01419-6.

3. Shang L, He W, Wang T, Yang Y, Xu Q, Zhao X, et al.. A complete assembly of the rice Nipponbare reference genome. *Molecular Plant*. 2023; doi: 10.1016/j.molp.2023.08.003.

4. Wu H, Luo L-Y, Zhang Y-H, Zhang C-Y, Huang J-H, Mo D-X, et al.. Telomere-to-telomere genome assembly of a male goat reveals variants associated with cashmere traits. *Nat Commun*. 2024; doi: 10.1038/s41467-024-54188-z.

5. Yoo D, Rhie A, Hebbar P, Antonacci F, Logsdon GA, Solar SJ, et al.. Complete sequencing of ape genomes. *Nature*. 2025; doi: 10.1038/s41586-025-08816-3.

6. Belton J-M, McCord RP, Gibcus JH, Naumova N, Zhan Y, Dekker J. Hi-C: a comprehensive technique to capture the conformation of genomes. *Methods*. 2012; doi: 10.1016/j.ymeth.2012.05.001.

7. Zhang T, Zhou J, Gao W, Jia Y, Wei Y, Wang G. Complex genome assembly based on long-read sequencing. *Briefings in Bioinformatics*. 2022; doi: 10.1093/bib/bbac305.

8. Li H, Durbin R. Genome assembly in the telomere-to-telomere era. *Nat Rev Genet*. 2024; doi: 10.1038/s41576-024-00718-w.

9. Logsdon GA, Ebert P, Audano PA, Loftus M, Porubsky D, Ebler J, et al.. Complex genetic variation in nearly complete human genomes. *Nature*. 2025; doi: 10.1038/s41586-025-09140-6.

10. Mosbruger TL, Dinou A, Duke JL, Ferriola D, Li Y, Hayeck TJ, et al.. Haplotypic resolution of the challenging genomic regions of MHC and KIR using a combination of targeted sequencing and a novel assembly pipeline. *Nucleic Acids Research*. 2025; doi: 10.1093/nar/gkaf441.

11. Yang Y, Du W, Li Y, Lei J, Pan W. Recent advances and challenges in *de novo* genome assembly. *GCOMM*. 2025; doi: 10.48130/gcomm-0025-0015.

12. Koren S, Rhie A, Walenz BP, Dilthey AT, Bickhart DM, Kingan SB, et al.. De novo assembly of haplotype-resolved genomes with trio binning. *Nat Biotechnol*. 2018; doi: 10.1038/nbt.4277.

13. Cheng H, Concepcion GT, Feng X, Zhang H, Li H. Haplotype-resolved de novo assembly using phased assembly graphs with hifiasm. *Nat Methods*. 2021; doi: 10.1038/s41592-020-01056-5.

14. Selvaraj S, R Dixon J, Bansal V, Ren B. Whole-genome haplotype reconstruction using proximity-ligation and shotgun sequencing. *Nat Biotechnol*. 2013; doi: 10.1038/nbt.2728.

15. Cheng H, Jarvis ED, Fedrigo O, Koepfli K-P, Urban L, Gemmell NJ, et al.. Haplotype-resolved assembly of diploid genomes without parental data. *Nat Biotechnol*. 2022; doi: 10.1038/s41587-022-01261-x.

16. Falconer E, Lansdorp PM. Strand-seq: A unifying tool for studies of chromosome segregation. *Seminars in Cell & Developmental Biology*. 2013; doi: 10.1016/j.semcdb.2013.04.005.

17. Human Genome Structural Variation Consortium, Porubsky D, Ebert P, Audano PA, Vollger MR, Harvey WT, et al.. Fully phased human genome assembly without parental data using single-cell strand sequencing and long reads. *Nat Biotechnol*. 2021; doi: 10.1038/s41587-020-0719-5.

18. Chin C-S, Peluso P, Sedlazeck FJ, Nattestad M, Concepcion GT, Clum A, et al.. Phased diploid genome assembly with single-molecule real-time sequencing. *Nat Methods*. 2016; doi: 10.1038/nmeth.4035.

19. Low WY, Tearle R, Liu R, Koren S, Rhie A, Bickhart DM, et al.. Haplotype-resolved genomes provide insights into structural variation and gene content in Angus and Brahman cattle. *Nat Commun*. 2020; doi: 10.1038/s41467-020-15848-y.

20. Ebert P, Audano PA, Zhu Q, Rodriguez-Martin B, Porubsky D, Bonder MJ, et al.. Haplotype-resolved diverse human genomes and integrated analysis of structural variation. *Science*. 2021; doi: 10.1126/science.abf7117.

21. Rhie A, McCarthy SA, Fedrigo O, Damas J, Formenti G, Koren S, et al.. Towards complete and error-free genome assemblies of all vertebrate species. *Nature*. 2021; doi: 10.1038/s41586-021-03451-0.

22. Kong W, Wang Y, Zhang S, Yu J, Zhang X. Recent Advances in Assembly of Complex Plant Genomes. *Genomics, Proteomics & Bioinformatics*. 2023; doi: 10.1016/j.gpb.2023.04.004.

23. Peona V, Weissensteiner MH, Suh A. How complete are “complete” genome assemblies?—An avian perspective. *Molecular Ecology Resources*. 2018; doi: 10.1111/1755-0998.12933.

24. Mathers TC, Paulini M, Sotero-Caio CG, Wood JMD. MicroFinder: Conserved gene-set mapping and assembly ordering for manual curation of bird microchromosomes. GigaScience. 2026; doi: 10.1093/gigascience/giag036

25. Zhao Q, Yin Z, Hou Z. Near telomere-to-telomere genome assemblies of Silkie Gallus gallus and Mallard Anas platyrhynchos restored the structure of chromosomes and “missing” genes in birds. *J Animal Sci Biotechnol*. 2025; doi: 10.1186/s40104-024-01141-1.

26. Huang Z, Xu Z, Bai H, Huang Y, Kang N, Ding X, et al.. Evolutionary analysis of a complete chicken genome. *Proc Natl Acad Sci USA*. 2023; doi: 10.1073/pnas.2216641120.

27. Luo H, Jiang X, Li B, Wu J, Shen J, Xu Z, et al.. A high-quality genome assembly highlights the evolutionary history of the great bustard (Otis tarda, Otidiformes). *Commun Biol*. 2023; doi: 10.1038/s42003-023-05137-x.

28. Hu J, Song L, Ning M, Niu X, Han M, Gao C, et al.. A new chromosome-scale duck genome shows a major histocompatibility complex with several expanded multigene families. *BMC Biol*. 2024; doi: 10.1186/s12915-024-01817-0.

29. Degrandi TM, Barcellos SA, Costa AL, Garnero ADV, Hass I, Gunski RJ. Introducing the Bird Chromosome Database: An Overview of Cytogenetic Studies in Birds. *Cytogenet Genome Res*. 2020; doi: 10.1159/000507768.

30. Baalsrud HT, Garmann-Aarhus B, Enevoldsen ELG, Krabberød AK, Fischer, Dominik, et al.. Centromeric satellite expansion drives genome evolution in the snowy owl. *Genome Biol*. 2026; doi: 10.1186/s13059-026-04175-y.

31. Belterman RHR, De Boer LEM. A karyological study of 55 species of birds, including karyotypes of 39 species new to cytology. *Genetica*. 1984; doi: 10.1007/BF00056765.

32. Machado AP, Cumer T, Iseli C, Beaudoing E, Ducrest A, Dupasquier M, et al.. Unexpected post-glacial colonisation route explains the white colour of barn owls (*Tyto alba*) from the British Isles. *Mol Ecol*. 2021; doi: 10.1111/mec.16250.

33. Ducrest A, Neuenschwander S, Schmid-Siegert E, Pagni M, Train C, Dylus D, et al.. New genome assembly of the barn owl (*Tyto alba alba*). *Ecology and Evolution*. 2020; doi: 10.1002/ece3.5991.

34. Topaloudis A, Cumer T, Lavanchy E, Ducrest A-L, Simon C, Machado AP, et al.. The recombination landscape of the barn owl, from families to populations. Wright S, editor. *GENETICS*. 2025; doi: 10.1093/genetics/iyae190.

35. Challis R, Richards E, Rajan J, Cochrane G, Blaxter M. BlobToolKit – Interactive Quality Assessment of Genome Assemblies. *G3 Genes|Genomes|Genetics*. 2020; doi: 10.1534/g3.119.400908.

36. Smith SA, Pease JB. Heterogeneous molecular processes among the causes of how sequence similarity scores can fail to recapitulate phylogeny. *Brief Bioinform*. 2017; doi: 10.1093/bib/bbw034.

37. Gozashti L, Harringmeyer OS, Hoekstra HE. How repeats rearrange chromosomes: The molecular basis of chromosomal inversions in deer mice. *Cell Reports*. 2025; doi: 10.1016/j.celrep.2025.115644.

38. Renzoni A, Vegni-Talluri M. The karyograms of some Falconiformes and Strigiformes. *Chromosoma*. 1966; doi: 10.1007/BF00335203.

39. Lang D, Zhang S, Ren P, Liang F, Sun Z, Meng G, et al.. Comparison of the two up-to-date sequencing technologies for genome assembly: HiFi reads of Pacific Biosciences Sequel II system and ultralong reads of Oxford Nanopore. *GigaScience*. 2020; doi: 10.1093/gigascience/giaa123.

40. Strand MA, Enevoldsen ELG, Tørresen OK, Skage M, Ferrari G, Tooming-Klunderud A, et al.. A haplotype-resolved bluethroat (*Luscinia s. svecica*) genome assembly uncovers the complex MHC region. *Journal of Heredity*. 2026; doi: 10.1093/jhered/esag058

41. Formenti G, Jain N, Medico JA, Sollitto M, Antipov D, Barcellos S, et al.. The complete genome of a songbird. *Cell.* 2026; doi: 10.1016/j.cell.2026.07.018

42. Hron T, Miklík D, Pačes J, Pajer P, Pečenka V, Hejnar J, et al.. Decoding the avian missing gene mystery: dot chromosomes unmask extensive gene loss and novel genetic instability. *Genome Biology and Evolution*. 2026; doi: 10.1093/gbe/evag038

43. Lundberg M, Liedvogel M, Larson K, Sigeman H, Grahn M, Wright A, et al.. Genetic differences between willow warbler migratory phenotypes are few and cluster in large haplotype blocks. *Evolution Letters*. 2017; doi: 10.1002/evl3.15.

44. Küpper C, Stocks M, Risse JE, Dos Remedios N, Farrell LL, McRae SB, et al.. A supergene determines highly divergent male reproductive morphs in the ruff. *Nat Genet*. 2016; doi: 10.1038/ng.3443.

45. Faria R, Johannesson K, Butlin RK, Westram AM. Evolving Inversions. *Trends in Ecology & Evolution*. 2019; doi: 10.1016/j.tree.2018.12.005.

46. Mahmoud M, Gobet N, Cruz-Dávalos DI, Mounier N, Dessimoz C, Sedlazeck FJ. Structural variant calling: the long and the short of it. *Genome Biol*. 2019; doi: 10.1186/s13059-019-1828-7.

47. Corval H, Cumer T, Topaloudis A, Collart F, Guisan A, Roulin A, et al.. Genetically diverse populations hold the keys to climatic adaptation in the Western barn owl (Tyto alba). *Commun Biol*. 2026; doi: 10.1038/s42003-026-10385-8.

48. Zhang M, Zhang Y, Scheuring CF, Wu C-C, Dong JJ, Zhang H-B. Preparation of megabase-sized DNA from a variety of organisms using the nuclei method for advanced genomics research. *Nat Protoc*. 2012; doi: 10.1038/nprot.2011.455.

49. Cumer T, Topaloudis A, Lavanchy E, Ducrest A-L, Cabello SS, Hewett A, et al.. Genomic bases of short-term evolution in the wild revealed by long-term monitoring and population-scale sequencing. bioRxiv. 2025; doi: 10.1101/2025.10.01.679793

50. Melters DP, Bradnam KR, Young HA, Telis N, May MR, Ruby JG, et al.. Comparative analysis of tandem repeats from hundreds of species reveals unique insights into centromere evolution. *Genome Biol*. 2013; doi: 10.1186/gb-2013-14-1-r10.

51. Meyne J, Ratliff RL, Moyzis RK. Conservation of the human telomere sequence (TTAGGG)n among vertebrates. *Proc Natl Acad Sci USA*. 1989; doi: 10.1073/pnas.86.18.7049.

52. Burri R, Antoniazza S, Siverio F, Klein Á, Roulin A, Fumagalli L. Isolation and characterization of 21 microsatellite markers in the barn owl (*Tyto alba*). *Molecular Ecology Resources*. 2008; doi: 10.1111/j.1755-0998.2008.02121.x.

53. Rebholz WER, Boer LEMD, Sasaki M, Belterman RHR, Nishida-Umehara C. The Chromosomal Phylogeny of Owls (Strigiformes) and New Karyotypes of Seven Species. *cytologia*. 1993; doi: 10.1508/cytologia.58.403.

54. Li H. Protein-to-genome alignment with miniprot. Valencia A, editor. *Bioinformatics*. 2023; doi: 10.1093/bioinformatics/btad014.

55. Simão FA, Waterhouse RM, Ioannidis P, Kriventseva EV, Zdobnov EM. BUSCO: assessing genome assembly and annotation completeness with single-copy orthologs. *Bioinformatics*. 2015; doi: 10.1093/bioinformatics/btv351.

56. Gurevich A, Saveliev V, Vyahhi N, Tesler G. QUAST: quality assessment tool for genome assemblies. *Bioinformatics*. 2013; doi: 10.1093/bioinformatics/btt086.

57. Li H. Minimap2: pairwise alignment for nucleotide sequences. *Bioinformatics*. 2018; doi: 10.1093/bioinformatics/bty191.

58. Camacho C, Coulouris G, Avagyan V, Ma N, Papadopoulos J, Bealer K, et al.. BLAST+: architecture and applications. *BMC Bioinformatics*. 2009; doi: 10.1186/1471-2105-10-421.

59. Girgis HZ. Red: an intelligent, rapid, accurate tool for detecting repeats de-novo on the genomic scale. *BMC Bioinformatics*. 2015; doi: 10.1186/s12859-015-0654-5.

60. Flynn JM, Hubley R, Goubert C, Rosen J, Clark AG, Feschotte C, et al.. RepeatModeler2 for automated genomic discovery of transposable element families. *Proc Natl Acad Sci USA*. 2020; doi: 10.1073/pnas.1921046117.

61. Smit AFA, Hubley R. RepeatModeler Open-1.0. 2008. http://www.repeatmasker.org

62. NCBI. EGAPx: Eukaryotic Genome Annotation Pipeline. GitHub. https://github.com/ncbi/egapx

63. Dobin A, Davis CA, Schlesinger F, Drenkow J, Zaleski C, Jha S, et al.. STAR: ultrafast universal RNA-seq aligner. *Bioinformatics*. 2013; doi: 10.1093/bioinformatics/bts635.

64. Souvorov A, Kapustin Y, Kiryutin B, Chetvernin V, Tatusova T, Lipman D. Gnomon – NCBI eukaryotic gene prediction tool. National Center for Biotechnology Information. 2010. https://www.ncbi.nlm.nih.gov/core/assets/genome/files/Gnomondescription.pdf

65. Li H. Aligning sequence reads, clone sequences and assembly contigs with BWA-MEM. 2013. arXiv; doi: 10.48550/arXiv.1303.3997

66. Iseli C, Ambrosini G, Bucher P, Jongeneel CV. Indexing Strategies for Rapid Searches of Short Words in Genome Sequences. Wu X, editor. *PLoS ONE*. 2007; doi: 10.1371/journal.pone.0000579.

67. Schindelin J, Arganda-Carreras I, Frise E, Kaynig V, Longair M, Pietzsch T, et al.. Fiji: an open-source platform for biological-image analysis. *Nat Methods*. 2012; doi: 10.1038/nmeth.2019.

68. Corval H, Cumer T. Chromosome-level, haplotype resolved assembly for the barn owl (Tyto alba). Zenodo. https://doi.org/10.5281/zenodo.19335597

69. Corval H, Guex N, Cumer T, Goudet J, Ducrest Roulin anne-lyse, Bachmann Salvy M, et al.. A Chromosome-level, haplotype resolved assembly for the barn owl (Tyto alba) - Numerical Data. Zenodo. https://zenodo.org/records/21887020

Figure 1

Click here to access/download;Figure;Figure_1.pdf

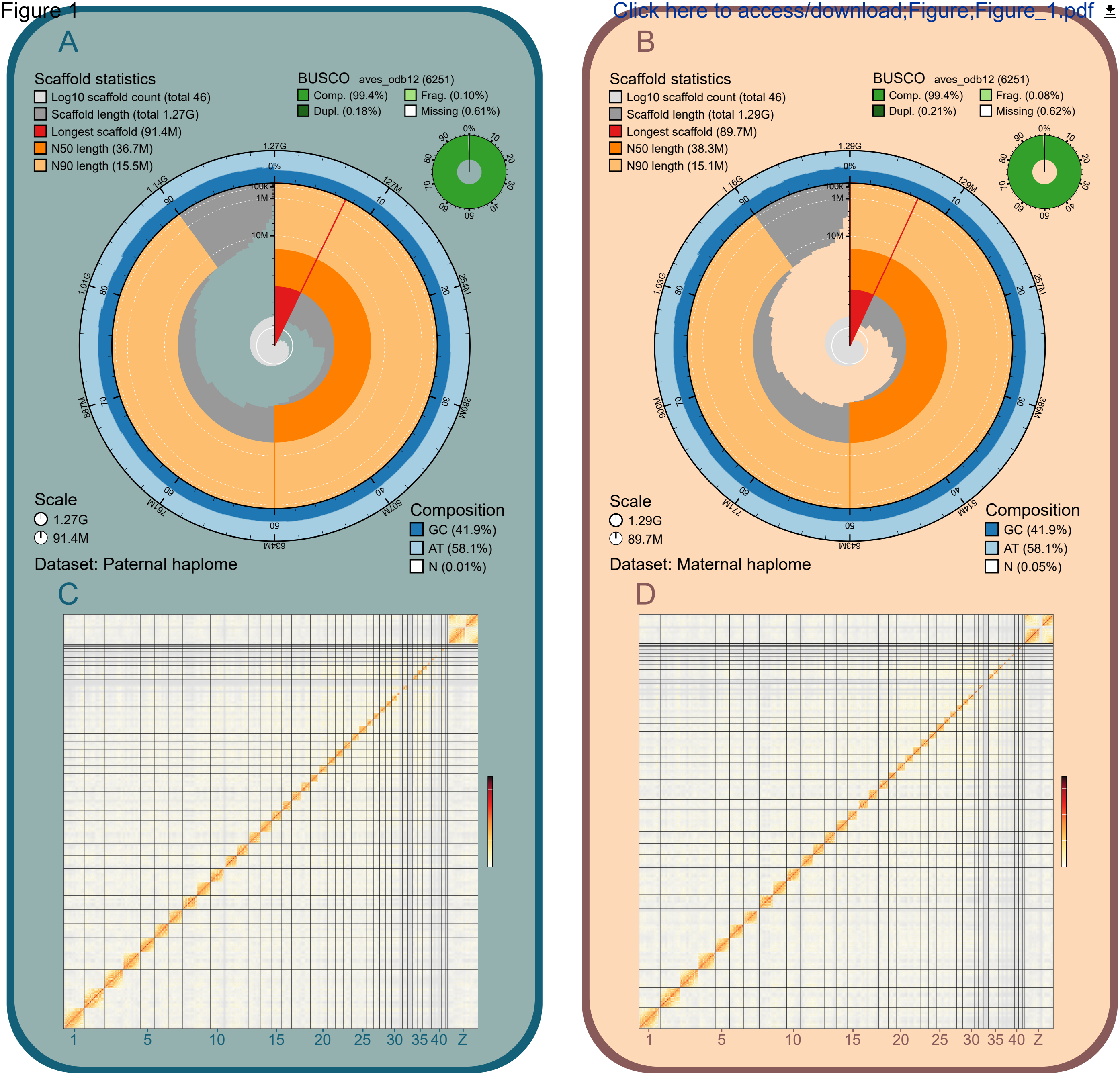

Figure 2

Click here to access/download;Figure;Figure_2.pdf

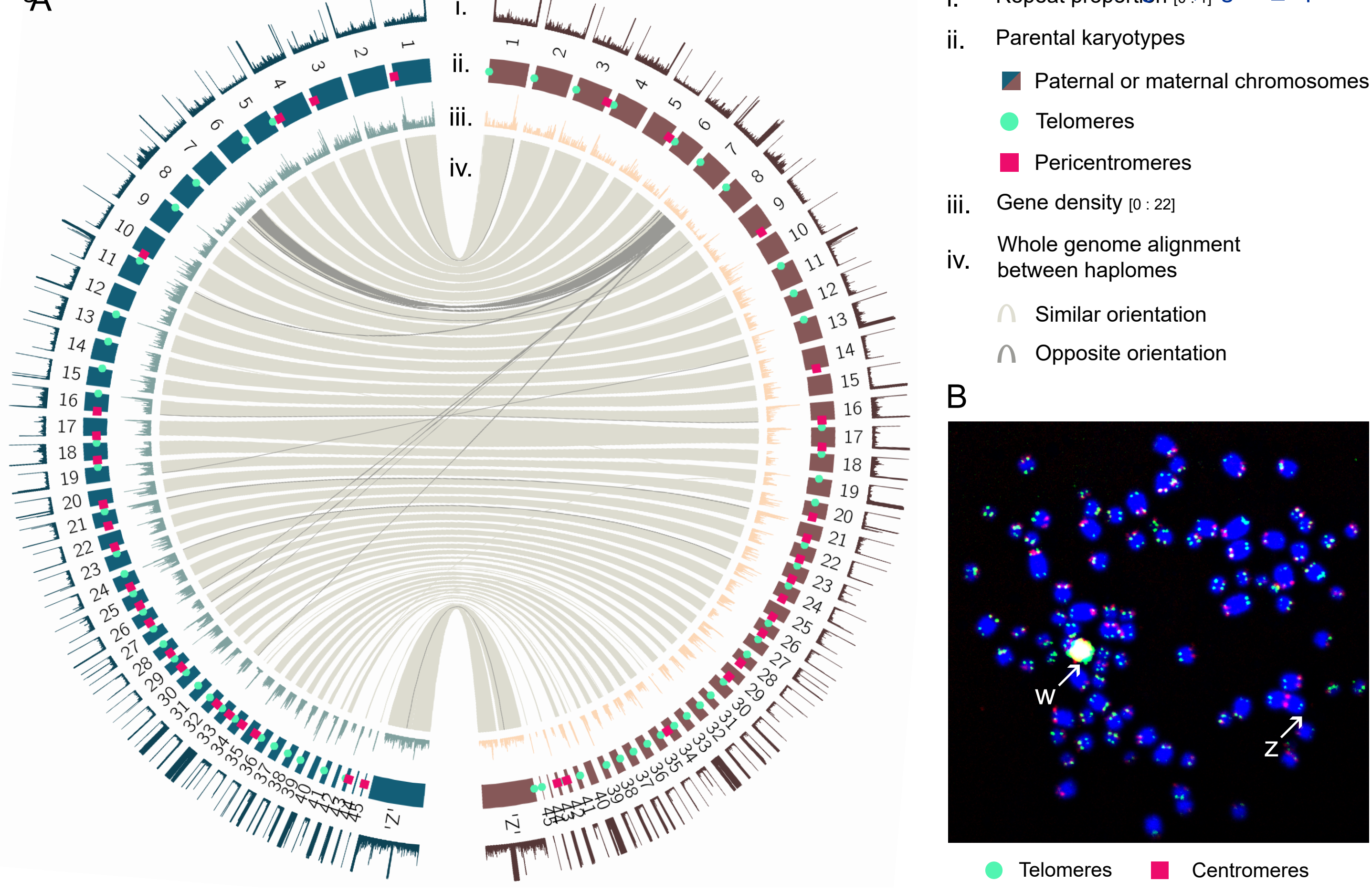

Figure 3

Click here to access/download;Figure;Figure_3.pdf

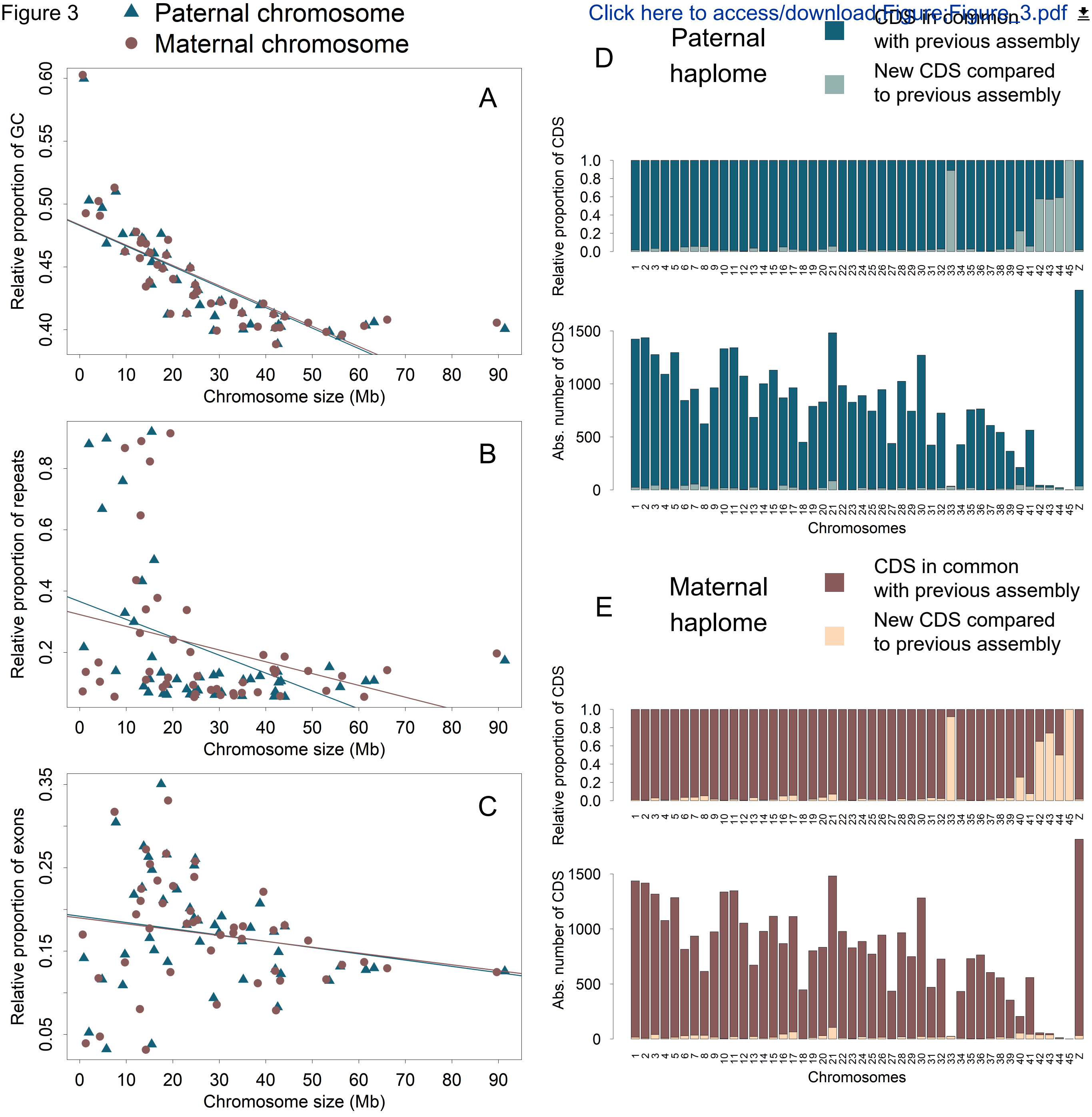

Figure 4

Click here to access/download;Figure;Figure_4.pdf

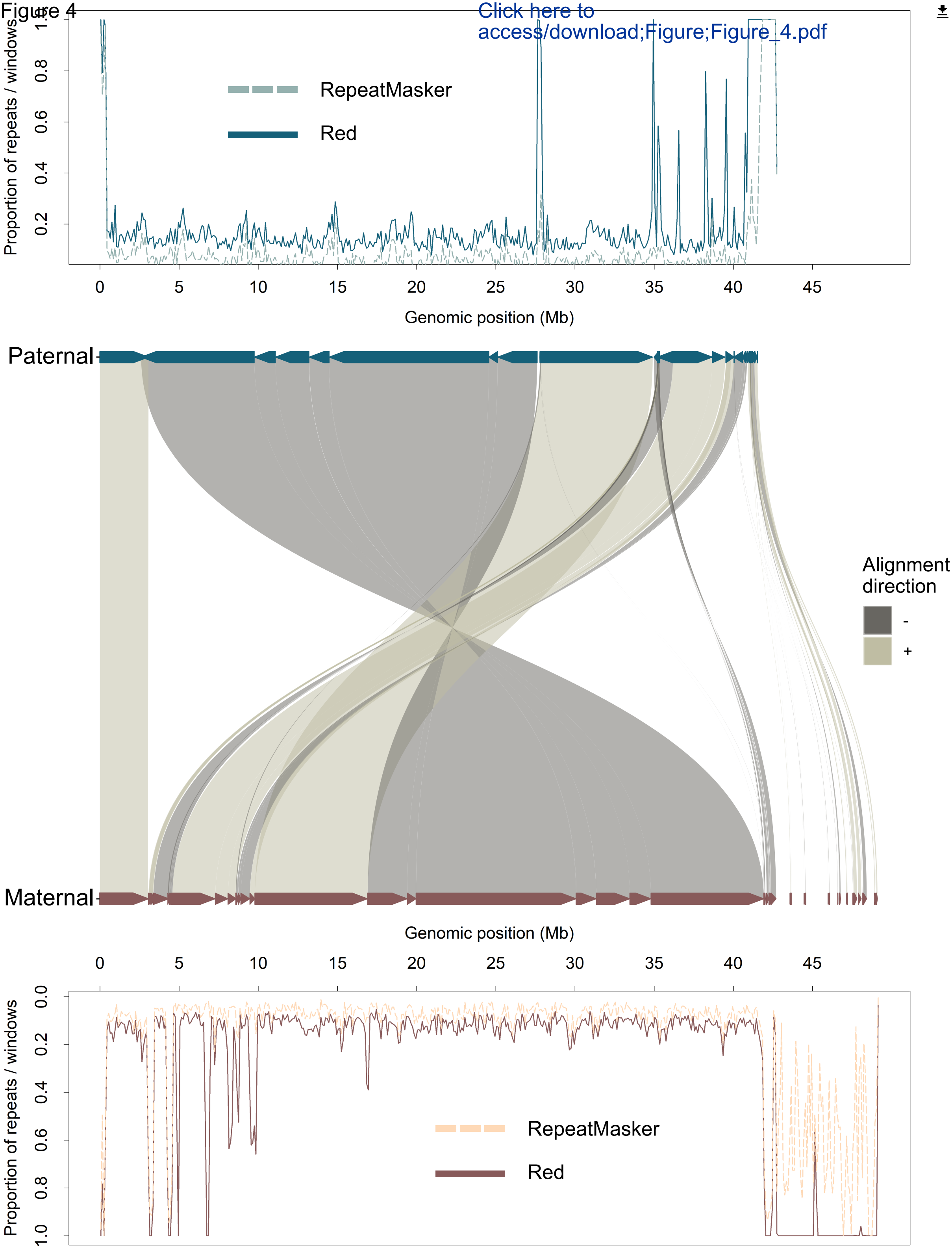

Supplementary Material table 2

Click here to access/download
Supplementary Material
SupplementaryTable_ChromosomeFeatures.xlsx

HiFiasm log file for reviewers

Click here to access/download
Supplementary Material
hifiasm.log

Incompatible reads for reviewers

Click here to access/download
Supplementary Material
incompatibleReads.txt

Incorrect Alignement for reviewers

Click here to access/download
Supplementary Material
incorrectAlignment.txt

Manuscript with tracked changes

Click here to access/download
Supplementary Material
NewRefGenome_GigaScience_v2_MainText_TrackedChanges.docx

Click here to access/download
Supplementary Material
NewRefGenome_GigaScience_v2_SuppMat (1).docx

Tristan CUMER
Department of Ecology and Evolution, Biophore
13 Building, University of Lausanne,
CH-1015 Lausanne, Switzerland.
Tristan.cumer@unil.ch

Dear Editors,

We are pleased to resubmit our manuscript GIGA-D-26-00095 entitled “A chromosome-level, haplotype-resolved genome assembly for the barn owl, *Tyto alba*” for publication in GigaScience.

In this new version, we’ve addressed all the points raised by the reviewers and we provide below a detailed answer to each.
We very much look forward to hearing whether this new version is suitable for publication in GigaScience.

with our best regards,
On the behalf of all co-authors,

Tristan Cumer
