# Supplementary material for: A chromosome-level, haplotype-resolved genome assembly for the barn owl, Tyto alba
Source: Gigascience. 2026 Sep 8;15:giag092. doi: 10.1093/gigascience/giag092 (PMC13615541; doi:10.1093/gigascience/giag092)
Supplement: Supplementary file 4 [file giag092_reviewer_1_report_original_submission.pdf]

Reviewer Report

Title: A chromosome-level, haplotype-resolved genome assembly for the barn owl, Tyto alba

Version: Original Submission **Date:** 4/9/2026

Reviewer name: Reviewer 1

Reviewer Comments to Author:

General comments:
It is always good that high quality genome assemblies are created and made available to the community. However, I have some comments below.
The authors have used a complicated assembly without justifying why this was done. One of the consequences of this was that the reads coming from the centromeres were excluded from the assembly, in addition to other reads. Instead of assembling the (HiFi) reads with hifiasm (or Verkko) and supplying k-mers from parents as is usually done for trio-assemblies, the authors separated reads before assembly. Both hifiasm versions used by the authors have had the ability to utilize HiFi and ONT reads at the same time (https://github.com/chhylp123/hifiasm?tab=readme-ov-file#ultra-long-ont-integration, the last example is directly applicable here). Why that approach, which has been used multiple times in the literature, was not chosen, and a custom approach was used instead is not clear to me. The authors should at least have demonstrated that the custom approach was superior to the "standard" approach, thereby justifying why it was done.
The manuscript does not report contig statistics. As far as I can see, all references to N50 are to scaffolds, and not contigs.The only ones referring to N50 contigs are to an old assembly. Knowing the N50 contig at different stages in the complicated assembly process would help us to understand how the process worked and how much it (potentially) improved the assemblies.
Several places in the manuscript it is stated that this is the "first haplotype-resolved and chromosome-level genome for Strigiformes". This is not justified well enough, and I point to several other assemblies that might fit this definition.
Specific comments:
Line 53: I find it a bit strange that you state that this is "first haplotype-resolved and chromosome-level genome for Strigiformes". Searching at GoaT (https://goat.genomehubs.org/) lists 5 Strigiformes with chromosome level assemblies (Athene noctua, Bubo scandiacus, Strix aluco, Strix nebulosa and Strix uralensis), where Athene noctua, Bubo scandiacus, and Strix aluco are all haplotype resolved. You even refer to the publication of one of these (Baalsrud HT et al 2025). Strix aluco has been on NCBI since September 2023, Athene noctua since February 2025 and Bubo scandiacus since March 2025. I guess you imply that these assemblies are only pseudo-haplotypes, but it is not clear to me.
Line 86: It is important to note that the sequence in the microchromosomes might be in the assembly, but it is difficult or impossible to distinguish from so-called shrapnel (unplaced sequence, often repeats).
Line 89: I don't understand your comment here "...adoption of these approaches has been uneven across birds…" Firstly, getting the correct data require proper handling of samples (flash-frozen ideally, etc), and successful DNA isolation before sequencing. These steps might be expensive, and not all

labs/projects might afford this. You write this as it is a conscious choice to not use ONT data, and I think it is mostly cost and other restrictions (maybe PacBio might be the only choice they have).
Figure 1: The resolution of this was a bit low, so hard to read the numbers.
Table 1: The N50 and N90 here, what do these refer to? It looks like scaffold metrics, but good to be explicit especially when "# contigs" is right above. How many contigs do you have in the final assemblies? It is written as 46 here, but you also have gaps, so I guess the number is higher. You have 46 chromosomes I guess.You cannot state "# contigs" and report scaffolds/chromosomes. Have a separate line for that or something (or just state it somewhere).
Figure 2: This figure is difficult to interpret. Colours are unclear, symbols are unclear (difficult to distinguish Similar and Opposite orientation). Text is diffuse. The green and red circles are really hard to see.
Figure 3: Again, this is hard to see. You need higher resolution. I don't know if this is the fault of the authors or the journal.
Figure 4: This is really nice. Where are the gaps in these two chromosomes? Does each "arrow" represent a contig?
Line 207: I understand what you want to say, but the T2T era is a consequence of better/longer reads, and not something that in itself has changed genome assembly.
Line 209: "among the most challenging" just depends on what you compare to, and what kind of tools you have available. In my experience, codfishes are awful to assemble with PacBio HiFi, but works excellent with ONT. Likewise here, HiFi leads to incomplete assemblies for birds, but ONT have much better results. For other species/groups, ONT might work really poorly and HiFi much better.
Line 212: I have already protested against the use of "first haplotype resolved, chromosome level assemblies for an owl". You have to define this better, that is, define this assembly such that the species mentioned above is not part of this definition.
Line 234: What do you mean by poor mappability here? We have seen good signals for microchromosomes. See for instance Supplementary Figure 1 here:
https://www.biorxiv.org/content/10.64898/2026.03.26.714473v1.full
Line 244: Or you could just generate enough ONT reads and skip HiFi altogether. See the paper linked above. While long is maybe good, you could just create a "normal" library with a high N50 read length (40 kb+), and 50-60x coverage and get almost T2T from only ONT.
Line 246: I guess you go into this in more detail in the methods, but as it is, I don't understand how and what you are filtering. Are you removing sequences from the assembly? Which you don't know for certain are not biological part of the genome?
Line 250: I don't think this list is complete. You seem to be lacking
https://www.biorxiv.org/content/10.1101/2025.10.14.682431v1 at least, and the paper linked above. Maybe many others also.
Lines 281-282: I am skeptical about your claim "more broadly, for the order
Strigiformes" as mentioned a couple of times above. I guess it also depends on the definitions of "complete" and "accurate".
Line 323: Which chemistry was used? R10 yields superior results compared to R9 usually.
Line 347: What is the definition of "extreme repeats"? I have not seen this approach earlier, and wonder about the benefits of doing it. Did this mean that you did not try to assemble the centromere?

Line 382: Multiple assemblers, such as Verkko and hifiasm, do this internally. It is not clear to me why you do this manually.
Line 413: There is a lot of information in reads shorter than 25k.
Line 440: Maybe I misunderstand something here. You orient the chromosomes so they start with p arm? (As recommended by EBP: https://www.earthbiogenome.org/report-on-assembly-standards [point l]). If you then add the centromeres (which can be of varying size), you will change all coordinates downstream, which means most of the chromosome since the q arm is longer. Is that correct? Since the centromere is not a point, you will most likely change the coordinates anyhow. I don't understand why you removed centromeric reads…
Line 446: How many contigs did you end up with? In table 1 it looks like you have just 46 contigs, but that's the number of chromosomes. You list the number of Ns in these assemblies also, so the amount of contigs is most likely many more than 46. You could mention how large your gaps are.
Line 501: You are replicating behaviour implemented in Merqury (https://github.com/marbl/merqury/wiki/3.-Phasing-assessment-with-hap-mers), which seems a bit strange to me. Meryl was published in the Merqury paper, so you could have seen this.

Methods

Are the methods appropriate to the aims of the study, are they well described, and are necessary controls included? Choose an item.

Conclusions

Are the conclusions adequately supported by the data shown? Choose an item.

Reporting Standards

Does the manuscript adhere to the journal's guidelines on minimum standards of reporting? Choose an item.

Choose an item.

Statistics

Are you able to assess all statistics in the manuscript, including the appropriateness of statistical tests used? Choose an item.

Quality of Written English

Please indicate the quality of language in the manuscript: Choose an item.

Declaration of Competing Interests

Please complete a declaration of competing interests, considering the following questions:

- Have you in the past five years received reimbursements, fees, funding, or salary from an organisation that may in any way gain or lose financially from the publication of this manuscript, either now or in the future?
- Do you hold any stocks or shares in an organisation that may in any way gain or lose financially from the publication of this manuscript, either now or in the future?
- Do you hold or are you currently applying for any patents relating to the content of the manuscript?
- Have you received reimbursements, fees, funding, or salary from an organization that holds or has applied for patents relating to the content of the manuscript?
- Do you have any other financial competing interests?
- Do you have any non-financial competing interests in relation to this paper?

If you can answer no to all of the above, write 'I declare that I have no competing interests' below. If your reply is yes to any, please give details below.

I declare that I have no competing interests

I agree to the open peer review policy of the journal. I understand that my name will be included on my report to the authors and, if the manuscript is accepted for publication, my named report including any attachments I upload will be posted on the website along with the authors' responses. I agree for my report to be made available under an Open Access Creative Commons CC-BY license (http://creativecommons.org/licenses/by/4.0/). I understand that any comments which I do not wish to be included in my named report can be included as confidential comments to the editors, which will not be published.

I agree to the open peer review policy of the journal

To further support our reviewers, we have joined with Publons, where you can gain additional credit to further highlight your hard work (see: https://publons.com/journal/530/gigascience). On publication of this paper, your review will be automatically added to Publons, you can then choose whether or not to claim your Publons credit. I understand this statement.

Yes Choose an item.
