# Supplementary material for: A chromosome-level, haplotype-resolved genome assembly for the barn owl, Tyto alba
Source: Gigascience. 2026 Sep 8;15:giag092. doi: 10.1093/gigascience/giag092 (PMC13615541; doi:10.1093/gigascience/giag092)
Supplement: Supplementary file 6 [file giag092_reviewer_2_report_original_submission.pdf]

Reviewer Report

Title: A chromosome-level, haplotype-resolved genome assembly for the barn owl, Tyto alba

Version: Original Submission **Date:** 5/4/2026

Reviewer name: Reviewer 2

Reviewer Comments to Author:

In this manuscript, the authors presented a chromosome-level, highly contiguous haplotype-resolved genome assembly of the barn owl. The manuscript is very well written and very detailed. I was, however, a bit confused by the duplicated manuscript in the file provided to me for review. My comments are based on the first version (pages 1-39 in the PDF).
General comments: Throughout the manuscript "-" are not shown but â€€€ instead.
Abstract:
L42: Calling microchromosomes "elements" seems very unspecific. I would maybe call them "DNA molecules"
Introduction:
L96: "70 "pseudo-haplotypes"" - pseudo haploid scaffolds or what does this exactly refer to?
Results:
L130: Why was a homogametic male chosen and not a heterogametic female? I know sometimes this is the only sample available but the authors went a great length to produce a very high-quality genome assembly but it is incomplete with the W chromosome missing.
L161: It is not clear to the reader here where this W chromosome now comes from and what FISH signals are. As this is the first mention of it, please have a short explanation of what FISH means in this context.
Figures and Tables:
Figure 1: Very nice design but the component and especially the text is too small to read without zooming in. Also in my version the resolution is poor making it even more difficult to read and look for details in the contact maps.
Figure 2: Also too small and low resolution in the review copy. Please make sure the text is readable, and the figures are large enough and high-resolution.
Figure 3: Text also too small I cannot reads the axis labels in my A4 printed version, especially in D and E.
Table 1: Tables should have a title on top, as it is a scientific standard, not a caption below.
Material and Methods:
L295: processed
L508 and following: I am missing more detailed QC steps. Like blobplots to identify potential contamination, Merqury QV and completeness values etc.
L511: Why was Busco 6 used but the old aves_odb10 not odb12?
L572 and 585: subheaders are difficult to identify as they are. Typesetting with likely makes them more visible, but I would at least add an empty line between sections for the next submission.

Methods

Are the methods appropriate to the aims of the study, are they well described, and are necessary controls included? Choose an item.

Conclusions

Are the conclusions adequately supported by the data shown? Choose an item.

Reporting Standards

Does the manuscript adhere to the journal's guidelines on minimum standards of reporting? Choose an item.

Choose an item.

Statistics

Are you able to assess all statistics in the manuscript, including the appropriateness of statistical tests used? Choose an item.

Quality of Written English

Please indicate the quality of language in the manuscript: Choose an item.

Declaration of Competing Interests

Please complete a declaration of competing interests, considering the following questions:

- Have you in the past five years received reimbursements, fees, funding, or salary from an organisation that may in any way gain or lose financially from the publication of this manuscript, either now or in the future?
- Do you hold any stocks or shares in an organisation that may in any way gain or lose financially from the publication of this manuscript, either now or in the future?
- Do you hold or are you currently applying for any patents relating to the content of the manuscript?
- Have you received reimbursements, fees, funding, or salary from an organization that holds or has applied for patents relating to the content of the manuscript?
- Do you have any other financial competing interests?
- Do you have any non-financial competing interests in relation to this paper?

If you can answer no to all of the above, write 'I declare that I have no competing interests' below. If your reply is yes to any, please give details below.

I declare that I have no competing interests

I agree to the open peer review policy of the journal. I understand that my name will be included on my report to the authors and, if the manuscript is accepted for publication, my named report including any attachments I upload will be posted on the website along with the authors' responses. I agree for my report to be made available under an Open Access Creative Commons CC-BY license (http://creativecommons.org/licenses/by/4.0/). I understand that any comments which I do not wish to be included in my named report can be included as confidential comments to the editors, which will not be published.

I agree to the open peer review policy of the journal

To further support our reviewers, we have joined with Publons, where you can gain additional credit to further highlight your hard work (see: https://publons.com/journal/530/gigascience). On publication of this paper, your review will be automatically added to Publons, you can then choose whether or not to claim your Publons credit. I understand this statement.

Yes Choose an item.
