# Supplementary material for: A chromosome-level, haplotype-resolved genome assembly for the barn owl, Tyto alba
Source: Gigascience. 2026 Sep 8;15:giag092. doi: 10.1093/gigascience/giag092 (PMC13615541; doi:10.1093/gigascience/giag092)
Supplement: Supplementary file 8 [file giag092_supplemental_files.zip › NewRefGenome_GigaScience_v2_SuppMat (1).docx]

**A chromosome-level, haplotype-resolved genome assembly for the barn owl, *Tyto alba* - Supplementary material**

**Supplementary Figures**

[Supplementary Figure 1	3](#_Toc232005117)

[Supplementary Figure 2	4](#_Toc232005118)

[Supplementary Figure 3	5](#_Toc232005119)

[Supplementary Figure 4	6](#_Toc232005120)

[Supplementary Figure 5	7](#_Toc232005121)

[Supplementary Figure 6	8](#_Toc232005122)

[Supplementary Figure 7	9](#_Toc232005123)

**Supplementary Tables**

[Supplementary Table 1	9](#_Toc231819103)

[Supplementary Table 2	10](#_Toc231819104)

[Supplementary Table 3	11](#_Toc231819105)

[Supplementary Table 4	12](#_Toc231819106)

[Supplementary Table 5	13](#_Toc231819107)

[Supplementary Table 6	14](#_Toc231819108)

[Supplementary Table 7	15](#_Toc231819109)

## Supplementary Figures

Supplementary Figure 1

**Miniprot alignments for the paternal proteome.** Protein alignments of the chicken and zebra finch proteome against the paternal haplome (only matches with ≥ 70% sequence identity and ≥ 90% target coverage retained). Each block represents a sequence in the assembly, and connecting lines indicate protein matches.

Supplementary Figure 2

**Miniprot alignments for the maternal proteome.** Protein alignments of the chicken and zebra finch proteome against the maternal haplome (only matches with ≥ 70% sequence identity and ≥ 90% target coverage retained). Each block represents a sequence in the assembly, and connecting lines indicate protein matches.

Supplementary Figure 3

**Contamination analysis of the paternal contigs**. Each circle corresponds to a single paternal contig, plotted according to its GC content (x-axis), and HiFi sequencing coverage (y-axis). Circle size is proportional to the true contig length (bp), with larger circles indicating longer contigs. Colours denote the taxonomic origin of the contig based on the most frequent BLAST hit, as specified in the *species* legend. Contigs labelled as “no-hit” correspond to sequences without significant BLAST matches.

Supplementary Figure 4

**Contamination analysis of the maternal contigs**. Each circle corresponds to a single maternal contig, plotted according to its GC content (x-axis), and HiFi sequencing coverage (y-axis). Circle size is proportional to the true contig length (bp), with larger circles indicating longer contigs. Colours denote the taxonomic origin of the contig based on the most frequent BLAST hit, as specified in the *species* legend. Contigs labelled as “no-hit” correspond to sequences without significant BLAST matches.

Supplementary Figure 5

**Repeat landscape of the paternal and maternal haplomes.** Proportions of repeat classes annotated with RepeatModeler and classified with RepeatMasker are shown for each parental haplome. Percentages are expressed relative to the entire repeat content of the corresponding haplome. It should be noted that the centromeric and pericentromeric repeats were not analysed here

Supplementary Figure 6

**PCA of chromosome level features (z-scored).** Principal component analysis was performed on the following features: (i) length, (ii) total exonic length (relative), (iii) GC content, (iv) gene density, (v) exon density, (vi) mean exon length, (vii) fraction of simple/low complexity repeats, and (viii) fraction of other RepeatMasker classes (overlaps resolved by the larger element’s class). Paternal chromosomes are shown in the left panel and maternal chromosomes in the right panel; chromosomes absent from the previous reference (GCF_018691265.1) are highlighted in lighter colours in both panels.

Supplementary Figure 7

**Fluorescence in situ hybridisation (FISH) visualisation of the *Tyto alba* karyotype of a female individual.** Chromosomes are counterstained with DAPI (blue). Centromeric regions are highlighted in magenta and telomeric regions in cyan using specific DNA probes (see Material and methods section for details). Sexual chromosomes Z and W are indicated by white arrows.

Supplementary Figure 8

**Mappability of the different chromosomes for the two parental haplomes.** Paternal haplome is displayed on the upper panel, the maternal haplome on the lower panel. Different colours correspond to different stringency thresholds used while measuring mappability.

## Supplementary Tables

| **File name** | **Read count** | **Average read length (bp)** |
| --- | --- | --- |
| m64046_200313_184523.hifi_reads.fa.gz | 1633571 | 14023 |
| m64046_200512_231235.hifi_reads.fa.gz | 1884925 | 13882 |
| m64156_220708_235329.hifi_reads.fa.gz | 1636736 | 23046 |
| m64156_220712_124431.hifi_reads.fa.gz | 1639724 | 22774 |
| m64156_230805_122943.hifi_reads.fa.gz | 1289514 | 20680 |
| ON1_25k.fq.gz | 1500988 | 48702 |
| ON2_25k.fq.gz | 755044 | 52234 |

Supplementary Table 1

**Summary of sequencing libraries.** PacBio HiFi and ONT libraries received from the Lausanne Genomic Technologies Facility (GTF) and the Gene Expression Core Facility (GECF), respectively, with the corresponding read count and mean read length (bp) for each library. In this table, the five HiFi libraries are listed first, followed by the two ONT libraries.

Supplementary Table 2

**Chromosome-level features of both haplomes.** Are listed (i) length, (ii) total exonic length (relative), (iii) GC, (iv) gene density, (v) exon density, (vi) mean exon length; (vii) fraction of simple/low complexity repeats and (viii) fraction of other RepeatMasker classes (resolving overlaps by the larger element’s class).

| **Chr** | **Paternal** | | | | **Maternal** | | | |
| --- | --- | --- | --- | --- | --- | --- | --- | --- |
|  | **Telomere** | **Length** | **Pericentromeres** | **Length** | **Telomere** | **Length** | **Pericentromeres** | **Length** |
| 1 |  |  | X | 23 | X | 9851 |  |  |
| 2 |  |  |  |  | X | 604 |  |  |
| 3 |  |  | X | 2132 | X | 5956 | X | 43 |
| 4 |  |  | X | 133 | X | 8967 |  |  |
| 5 | X | 4041 |  |  |  |  | X | 9851 |
| 6 | X | 8905 |  |  | X | 9958 |  |  |
| 7 |  |  |  |  | X | 9042 |  |  |
| 8 | X | 9797 |  |  | X | 9871 |  |  |
| 9 | X | 1712 |  |  |  |  | X | 329 |
| 10 |  |  | X | 358 |  |  |  |  |
| 11 | X | 9941 |  |  | X | 9970 |  |  |
| 12 |  |  |  |  | X | 305 |  |  |
| 13 | X | 3502 |  |  | X | 7406 |  |  |
| 14 | X | 9728 |  |  |  |  | X | 30 |
| 15 | X | 7262 |  |  |  |  |  |  |
| 16 | X | 1234 | X | 13 |  |  | X | 342 |
| 17 |  |  | X | 52 | X | 6892 | X | 23 |
| 18 | X | 9670 | X | 25 | X | 6243 |  |  |
| 19 | X | 9656 |  |  | X | 5730 |  |  |
| 20 |  |  | X | 153 | X | 8895 | X | 160 |
| 21 | X | 7829 | X | 401 |  |  | X | 128 |
| 22 |  |  | X | 4594 | X | 7332 | X | 361 |
| 23 | X | 8689 |  |  | X | 2285 | X | 25 |
| 24 | X | 7844 | X | 9507 | X | 8253 | X | 167 |
| 25 | X | 8348 | X | 357 |  |  | X | 2598 |
| 26 | X | 9287 | X | 116 | X | 8362 | X | 318 |
| 27 | X | 172 |  |  | X | 9201 |  |  |
| 28 | X | 2974 | X | 280 | X | 4290 | X | 75 |
| 29 | X | 5996 | X | 3163 |  |  | X | 2513 |
| 30 | X | 9934 |  |  | X | 7646 |  |  |
| 31 | X | 6758 |  |  | X | 6979 |  |  |
| 32 | X | 1422 | X | 3592 | X | 8103 |  |  |
| 33 | X | 8004 | X | 16764 | X | 7837 |  |  |
| 34 | X | 4780 | X | 4938 | X | 9683 | X | 6450 |
| 35 |  |  | X | 4077 | X | 9814 |  |  |
| 36 | X | 1686 |  |  | X | 9191 |  |  |
| 37 | X | 8261 |  |  | X | 9062 |  |  |
| 38 | X | 6664 |  |  | X | 1080 |  |  |
| 39 | X | 7856 |  |  | X | 824 |  |  |
| 40 |  |  |  |  |  |  |  |  |
| 41 | X | 5455 |  |  | X | 2237 |  |  |
| 42 |  |  |  |  |  |  | X | 344 |
| 43 | X | 9947 | X | 244 | X | 754 | X | 93 |
| 44 |  |  |  |  |  |  |  |  |
| 45 | X | 2784 | X | 9570 | X | 3381 |  |  |
| 46 |  |  |  |  | X | 5252 |  |  |

Supplementary Table 3

**Terminal features per chromosome.** For each parental chromosome, presence/absence of telomeric and pericentromeric sequence and the corresponding span (bp).

| **Set of 49-mers** | **Distinct** | **Unique** | **Total** | **Maximum count** |
| --- | --- | --- | --- | --- |
| father.jf49.L1.Uinf.selection1 | 3594562817 | 2096246897 | 9068873521 | 9273511 |
| father.jf49.L1.Uinf.selection2 | 3594043171 | 2095620581 | 9069098779 | 9290946 |
| father.jf49.L1.Uinf.selection3 | 3561856628 | 2066495849 | 9060796066 | 9632777 |
| mother.jf49.L1.Uinf.selection1 | 3116725701 | 1568256693 | 9079234142 | 5416817 |
| mother.jf49.L1.Uinf.selection2 | 3116301751 | 1567585954 | 9079153908 | 4382415 |

Supplementary Table 4

**Counts and composition of parental 49-mer libraries.** For each parental 49‑mer library (three paternal, two maternal), we report the number of distinct 49‑mers, the number of unique 49‑mers, the total 49‑mer observations, and the maximum per‑49‑mer copy count.

| **Parent specific 49-mers set** | **Distinct** | **Total** | **Maximum count** |
| --- | --- | --- | --- |
| only_father.selection1.NOTmother1and2 | 179277071 | 548680948 | 3026 |
| only_father.selection2.NOTmother1and2 | 179332652 | 548753287 | 3086 |
| only_father.selection3.NOTmother1and2 | 180935695 | 556322694 | 3132 |
| only_mother.selection1.NOTfather1and2 | 188431608 | 622399767 | 728 |
| only_mother.selection1.NOTfather1and3 | 188853699 | 623561440 | 513 |
| only_mother.selection1.NOTfather2and3 | 188821563 | 623729275 | 613 |
| only_mother.selection2.NOTfather1and2 | 188549060 | 622942205 | 797 |
| only_mother.selection2.NOTfather1and3 | 188929355 | 624037708 | 483 |
| only_mother.selection2.NOTfather2and3 | 188912686 | 624182568 | 511 |

Supplementary Table 5

**Parent-private 49-mers.** Paternal and maternal 49mer sets used identify parent-specific sequences. 49mers contain no Ns and are considered parent-private when present ≥ 2× in a subset of one parent and absent from subsets of the other parent.

| **Categories** | **Father** | **Mother** | **Both/Unclassified** |
| --- | --- | --- | --- |
| Centromere-like | 12,575 | 7,450 | 5,411 |
| Kinetochore-like | 187,463 | 37,388 | 8,132 |
| Microsatellite | 116,684 | 117,378 | 16,532 |
| Telomere | 3,273 | 2,876 | 125 |
| Other | 3,714,919 | 3,626,898 | 227,366 |

Supplementary Table 6

**HiFi reads parental assignment by category.** The number of paternal, maternal and unclassified reads for each of the read category determined by their repeat profile (see *Identification of repeated sequences and read filtering* section for details).

| **Categories** | **Father** | **Mother** | **Both/Unclassified** |
| --- | --- | --- | --- |
| Centromere-like | 4,635 | 2,069 | 1,366 |
| Kinetochore-like | 74,039 | 17,016 | 5,305 |
| Microsatellite | 18,940 | 20,744 | 2,405 |
| Telomere | 1,173 | 1,040 | 98 |
| Other | 438,778 | 437,726 | 103,389 |

Supplementary Table 7

**ONT (>= 25 kbp) reads parental assignment by category.** The number of paternal, maternal and unclassified reads for each of the read category determined by their repeat profile (see *Identification of repeated sequences and read filtering* section for details).
